# CircularLRRC7 is a Potential Tumor Suppressor Associated With miR-1281 and PDXP Expression in Glioblastoma

**DOI:** 10.3389/fmolb.2021.743417

**Published:** 2021-11-29

**Authors:** Xue Kong, Ruiting Xu, Wei Wang, Minghui Zeng, Yuan Li, Mengyu Lin, Wenchao Zhou, Xianming Fu, Haibo Wu

**Affiliations:** ^1^ School of Medicine, Shandong University, Jinan, China; ^2^ Department of Neurosurgery, Anhui Provincial Hospital, Shandong University, Hefei, China; ^3^ Department of Pathology, The First Affiliated Hospital of USTC, Division of Life Sciences and Medicine, University of Science and Technology of China, Hefei, China; ^4^ Intelligent Pathology Institute, The First Affiliated Hospital of USTC, Division of Life Sciences and Medicine, University of Science and Technology of China, Hefei, China; ^5^ Department of Neurosurgery, The First Affiliated Hospital of USTC, Division of Life Sciences and Medicine, University of Science and Technology of China, Hefei, China

**Keywords:** circRNA, hsa_circ-0114014, GBM, miRNA, miR-1281, PDXP, tumor suppressor

## Abstract

Circular RNAs (circRNAs) are usually enriched in neural tissues, yet about 80% circRNAs have lower expression in gliomas relative to normal brains, highlighting the importance of circRNAs as tumor suppressors. However, the clinical impact as well as the pathways regulated by the tumor-suppressive circRNAs remain largely unknown in glioblastoma (GBM). Through bioinformatic analysis followed by experimental validation, we found that hsa_circ_0114014 (circLRRC7) was dramatically down-regulated in GBM when compared with normal brain tissues (*p* < 0.0001). GBM patients with a lower circLRRC7 expression had poorer progression-free survival (PFS, *p* < 0.05) and overall survival (OS, *p* < 0.05). Analyses of the predicted target miRNAs of circLRRC7 in CSCD and CRI databases, in combination with the miRNA expression data in GBMs and normal brains from GSE database, revealed miR-1281 as a potential downstream target of circLRRC7. Subsequently, the target genes of hsa-mir-1281 were predicted by TargetScan, miRDB and miRNATAR databases. Intersection analysis and correlation test indicated that PDXP was a potential target of miR-1281. In summary, circLRRC7 may be a tumor suppressor that associated with miR-1281 and PDXP expression in GBM, which may provide novel therapeutic targets for GBM treatment.

## Introduction

GBM (glioblastoma multiforme) is the most malignant brain tumor with the highest mortality rate (5-years survival rate 3–5%) and shortest median survival period (approximately 1.4-years) (Van Meir et al., 2010). Radical surgery combined with concomitant chemoradiation therapy based on temozolomide is still the primary treatment strategy for GBM which has been used for over 2 decades. Although the antiangiogenic drug bevacizumab was approved for patients with recurrent GBM, there’s little or no improvement in patients’ overall survival (Ameratunga et al., 2018). These facts urge the investigation of new therapeutics for GBM treatment.

Circular RNAs (circRNAs) are a group of novel RNA molecules with covalently closed loop that generally exist in eukaryotes (HSU and Coca prados, 1979; Jeck et al., 2013). Highly conserved among species, circRNAs have tissue-specific expression and are especially enriched in neuronal tissues (Song X et al., 2016). This phenomenon might be due to the abundance of coding host genes, splicing factors and RNA binding proteins in the brain (Chen et al., 2016). CircRNAs may function as “miRNA sponge” (Tay et al., 2014), “protein sponge” (Schneider et al., 2016), transcriptional regulators (Li et al., 2015), and even coding sequences for peptides, indicating their potential as drug targets for cancer treatment (Wesselhoeft et al., 2018; Wu et al., 2020). An increasing number of researches have demonstrated the crucial roles of circRNAs in glioma progression. For example, circRNA MMP9 functions as a sponge to absorb miR-124 and thus promotes GBM progression (Wang et al., 2018). Meanwhile, circZNF292 promotes glioma proliferation and cell cycle progression (Yang et al., 2016). Likewise, circ-U2AF1 sponges hsa-miR-7-5p to derepress NOVA2 and thus augments glioma malignancy (Li et al., 2018). Moreover, circRNAs could be potential biomarkers for diagnosis and prognosis of GBM (Zhu et al., 2017; Li et al., 2018; Li et al., 2018). Interestingly, the abundance of most circRNAs is much lower in gliomas than in normal brain tissues (Westholm et al, 2014; Song et al., 2016), indicating that circRNAs may function as tumor suppressors, which has not been elucidated so far.

In this study, using bioinformatic methods, we identified circLRRC7 as the most dramatically downregulated circRNA in GBM and predicted miR-1281 and PDXP as the downstream genes of circLRRC7. The expression of circLRRC7 in GBMs vs. normal brains was validated and the correlation between circLRRC7, miR-1281 and PDXP was confirmed in GBM patients. These findings suggest a novel tumor suppressive circRNA and shed light on new therapeutic strategies for GBM treatment.

## Material and Methods

### Acquisition of Clinical Specimens and qRT-PCR

28 GBM specimens and 23 normal brain tissues were obtained from patients who received surgery at The First Affiliated Hospital of the University of Science and Technology of China (USTC) from 2019 to 2020. As to the normal brain tissues, 3 samples were GBM adjacent surgical margin (paired samples), and the rest 20 samples were from epilepsy patients. This study was approved by the Ethics Committee of The First Affiliated Hospital of USTC. Informed consent was acquired from each patient. The clinical, surgical, imaging and pathological records were reviewed retrospectively. The pathological diagnosis was validated by two pathologists independently. The survival time was calculated from the date of diagnosis of GBM until October 16, 2020. The expression of potential circRNAs, miRNAs and mRNAs in resected tissues was tested through qRT-PCR. The primers used in this study were synthesized by Sangon Biotech.Ltd. (Shanghai, China) (Table 1). QRT-PCR was set at an initial denaturation step of 10 min at 95°C and 95°C for 10 s, 60°C for 20 s, and 72°C for 10 s for a total of 40 cycles. The relative circRNAs expression levels were normalized to GAPDH.

**TABLE 1:** The primers of circular RNAs.

Circular RNA	Forward primer	Reverse primer
hsa_circ_0073237	GCA​GCA​TCA​GAA​CAG​CAA​GT	TCA​CTC​ATT​CGA​CCT​GGT​AAA​T
hsa_circ_0024085	ATC​TTC​TCA​GCC​TGC​CCA​AT	GGAACCCTGGAGCTGTCT
hsa_circ_0009027	GGG​CCT​ACA​CCT​CGA​ATC​C	CTT​GTC​CTT​GTT​GTG​CTG​GC
hsa_circ_0007513	AAC​CAT​GCC​TCC​ATC​ATC​GA	GAT​TCT​TGT​GGA​TGC​TCT​GGG
hsa_circ_0021350	GCA​ACA​GAT​CCA​GGT​ACA​CC	TTC​TGT​TCA​TCA​TGC​GCT​GC
hsa_circ_0131934	CAA​CAA​GCA​ATA​CAA​GAC​CAA​GT	GCT​GTG​CAG​GAA​AAT​AGA​GCT
hsa_circ_0055954	CAT​CTT​CTG​GTT​TCA​TTG​GTT​CA	GAA​GGA​GAG​GGA​GCT​GGG​TA
hsa_circ_0114014	GGG​CAA​AGG​AGT​ATG​GAT​GG	GAC​GGG​GTC​TGA​AGG​ATC​TT
hsa_circ_0078784	CCT​GCT​TGA​CAA​CCA​GTA​CTA	CTC​CAT​GAA​AAC​CGC​TGC​AT
hsa_circ_0092798	GAC​GGA​GAG​AGA​CAG​CCT​TT	TGA​ACC​GAC​AGA​CCA​CGT​AA

### Statistical Analysis

Chi-square test and Fisher’s test and plotting receiver operating characteristic curves (ROCs) was used to analyze relevant factors by SPSS20.0 (SPSS, Inc., Chicago). The Kaplan-Meier method was employed in estimating the survival rate. The Cox proportional hazards regression analysis was used in evaluating multivariate prognosis factors by SPSS20.0. Relation for circRNA-miRNA, miRNA-mRNA and circRNA-mRNA was performed by Person correlation coefficient using Graphpad Prism software (version 9). Results were regarded as statistically significant when *p*-value < 0.05.

### Datasets Collection

The circRNA dataset in this study was obtained from NCBI GEO database (http://www.ncbi.nlm.nih.gov/) GSE147352 (15 normal brain tissues and 18 gliomas). The miRNA expression dataset derived from GEO GSE158284 (12 normal brain tissues and 20 gliomas) and mRNA expression profile were gained from TCGA database (http://cancergenome.nih.gov/), including 5 normal brain tissues and 169 gliomas.

### Differential Expression Analysis of circRNAs, miRNAs and mRNAs

All raw data were normalized and log2-transformed. The ID of the corresponding probe name was converted into gene symbols. We identified differentially expressed RNAs through R statistical software and the limma Bioconductor package. The criteria for screening differentially expressed circRNAs, miRNAs (DEmiRNAs) and mRNAs (DEmRNAs) was *p*-value <0.05 and |log2FC(fold change)|>1.

### Prediction of circRNAs-miRNAs-mRNAs Network

The Cancer-Specific circRNA (CSCD, http://gb.whu.edu.cn/CSCD/) and the circular RNA Interactome (https://circinteractome.nia.nih.gov/) were used to get the overlapping target miRNAs (TmiRNAs) as candidate targets of the selected circRNAs. The common members of TmiRNAs and DEmiRNAs were obtained the potential targeted miRNAs for further analyses. The interactions between miRNAs and mRNAs were predicted through TargetScan (http://www.targetscan.org), miRBD (http://www.mirdb.org/) and miRNATAR (http://microrna.gr/tarbase/). Overlapping mRNAs called TmRNAs were picked up and further intersected with DEmRNAs to obtain targeted mRNAs. The prognostic values of mRNAs in GBM were evaluated by Gliovis database (http://gliovis.bioinfo.cnio.es/) using Kaplan-Meier analysis.

### Function Enrichment Analysis

The differentially expressed circRNAs were annotated with Circbase database (http://circrna.org/) to obtain informations of corresponding gene, then these genes were analyzed with Gene Ontology (GO) analysis. Differentially expressed mRNAs were studied with GO and Kyoto Encyclopedia of Genes and Genomes (KEGG) analysis. The function of circRNAs and mRNAs were presumed according to GO and KEGG analyses by using KOBAS website (http://kobas.cbi.pku.edu.cn/kobas3/).

## Results

### Identification of circLRRC7 as the Most Differentially Expressed circRNA in GBM

**FIGURE 1:**
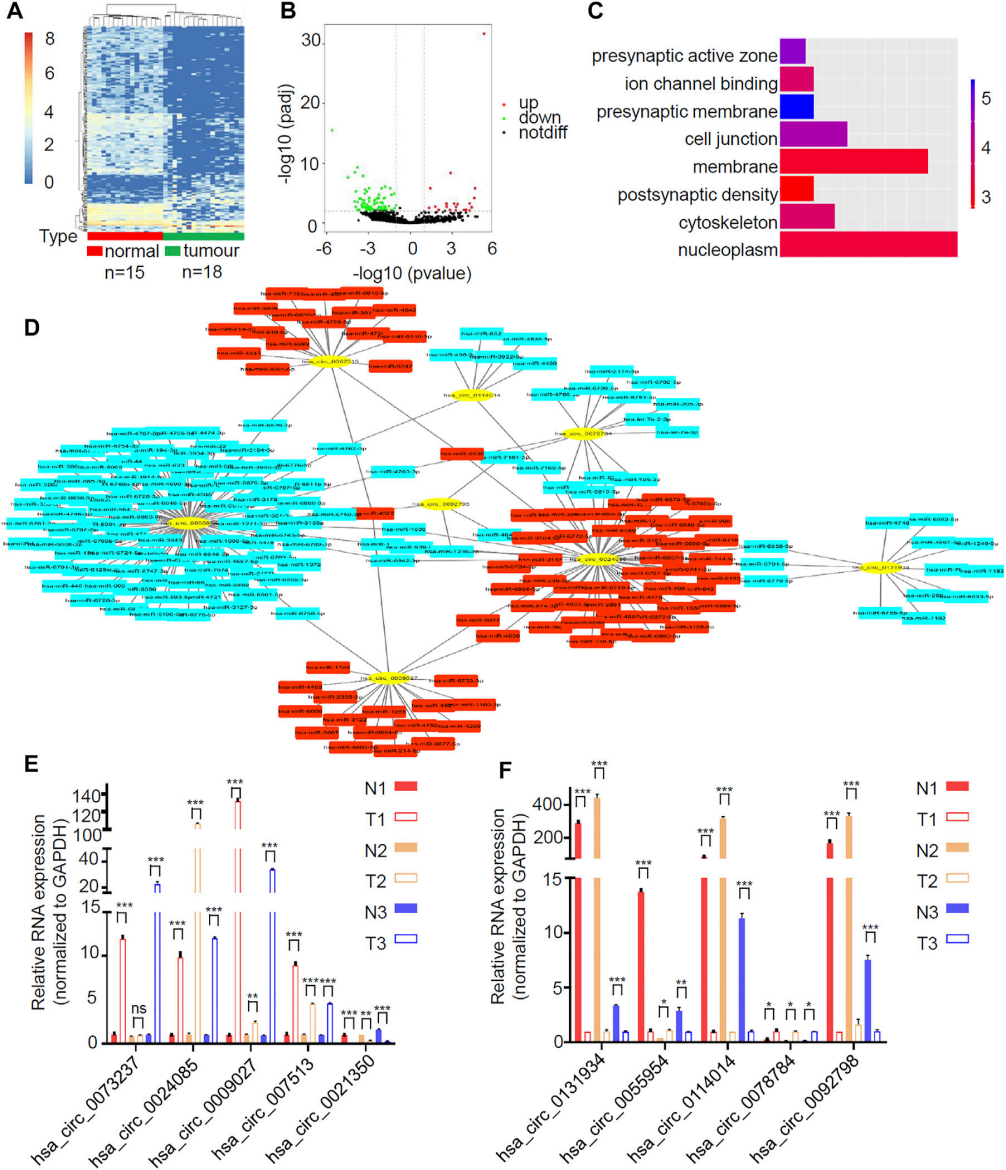
circLRRC7 was the most significantly differentially expressed circRNA in GBM relative to normal brain. **(A)** Clustered heatmap showed tissue-specific circRNAs between 15 normal brain tissues and 18 gliomas. The color scale indicates the log2 value of the ratio of the differential gene expression (red represents up-regulation; blue represents down-regulation). **(B)** Volcano plot of differentially expressed circRNAs. The red dots and green dots represent upregulated circRNAs and downregulated circRNAs with significance (|logFC| > 1, padj<0.05). **(C)** GO analysis of differentially expressed circRNAs. **(D)** circRNA-miRNA network of the top 10 differentially expressed circRNAs. **(E,F)** The relative expression of the top 10 differentially expressed circRNAs in 3 pair of GBM samples and adjacent surgical margin samples with qRT-PCR. (ns, *p* > 0.05; *p < 0.05; **p < 0.01; ***p < 0.001).

To detect the circRNAs specifically expressed in gliomas, we analyzed the GEO dataset (GSE147352) that determined the expression of all circRNAs in 15 normal brain tissues and 18 gliomas through high throughput sequencing. Among the 8,184 detected circRNAs, 38 upregulated circRNAs and 186 downregulated circRNAs were identified in gliomas in accordance with the criteria |log2FC| > 1, padj <0.05 (Figures 1A,B; Supplementary Table S1). GO analysis showed that the differentially expressed circRNAs were significantly enriched in 8 GO categories, including nucleoplasm, cytoskeleton, postsynaptic density, membrane, cell junction, presynaptic membrane, ion channel binding and presynaptic active zone (Figure 1C). We picked up the top 5 up- and down-regulated circRNAs (Table 2) and tried to construct the circRNA-miRNA network through cytoscape software. Finally, we got the predicted circRNA-miRNA network for 8 circRNAs (hsa_circ_0073237, hsa_circ_0024085, hsa_circ_0009027, hsa_circ_007513, hsa_circ_0131934, hsa_circ_0055954, hsa_circ_0114014 and hsa_circ_0078784), whereas the rest 2 circRNAs (hsa_circ_0021350, and hsa_circ_0092798) were not able to be incorporated (Figure 1D). To investigate the expression pattern of these circRNAs in GBM, 3 pairs of samples of GBM and the matched adjacent surgical margins from our hospital were employed for qRT-PCR analyses. Among the 6 validated circRNAs (hsa_circ_0024085, hsa_circ_0009027, hsa_circ_007513, hsa_circ_0131934, hsa_circ_0114014 and hsa_circ_0092798) with consistent expression patterns in qRT-PCR and the RNA-sequencing, hsa_circ_0114014 (circLRRC7) demonstrated the most obvious downregulation in GBM samples (Figures 1E,F), suggesting an important role of this circRNA in GBM.

**TABLE 2:** The differentially expressed circRNAs with significance (|logFC| > 1, *p*-value<0.05) between normal brain and glioma tissues.

circRNA name	*p*-value	B	Log_2_FC
hsa_circ_0073237	1.24E-35	105.0081668	5.284232218
hsa_circ_0024085	1.19E-08	3.615830329	4.63877503
hsa_circ_0009027	1.08E-06	3.750267651	4.576516122
hsa_circ_0007513	0.00011552	1.411585086	4.398741595
hsa_circ_0021350	1.63E-05	1.227686718	4.245986517
hsa_circ_0131934	3.54E-19	5.220663592	−5.616262636
hsa_circ_0055954	1.08E-10	4.342061264	−4.47807931
hsa_circ_0114014	6.58E-06	1.699945287	−3.987167056
hsa_circ_0078784	7.82E-12	5.253227992	−3.96307385
hsa_circ_0092798	9.34E-05	1.603328349	−3.913020024

**FIGURE 2:**
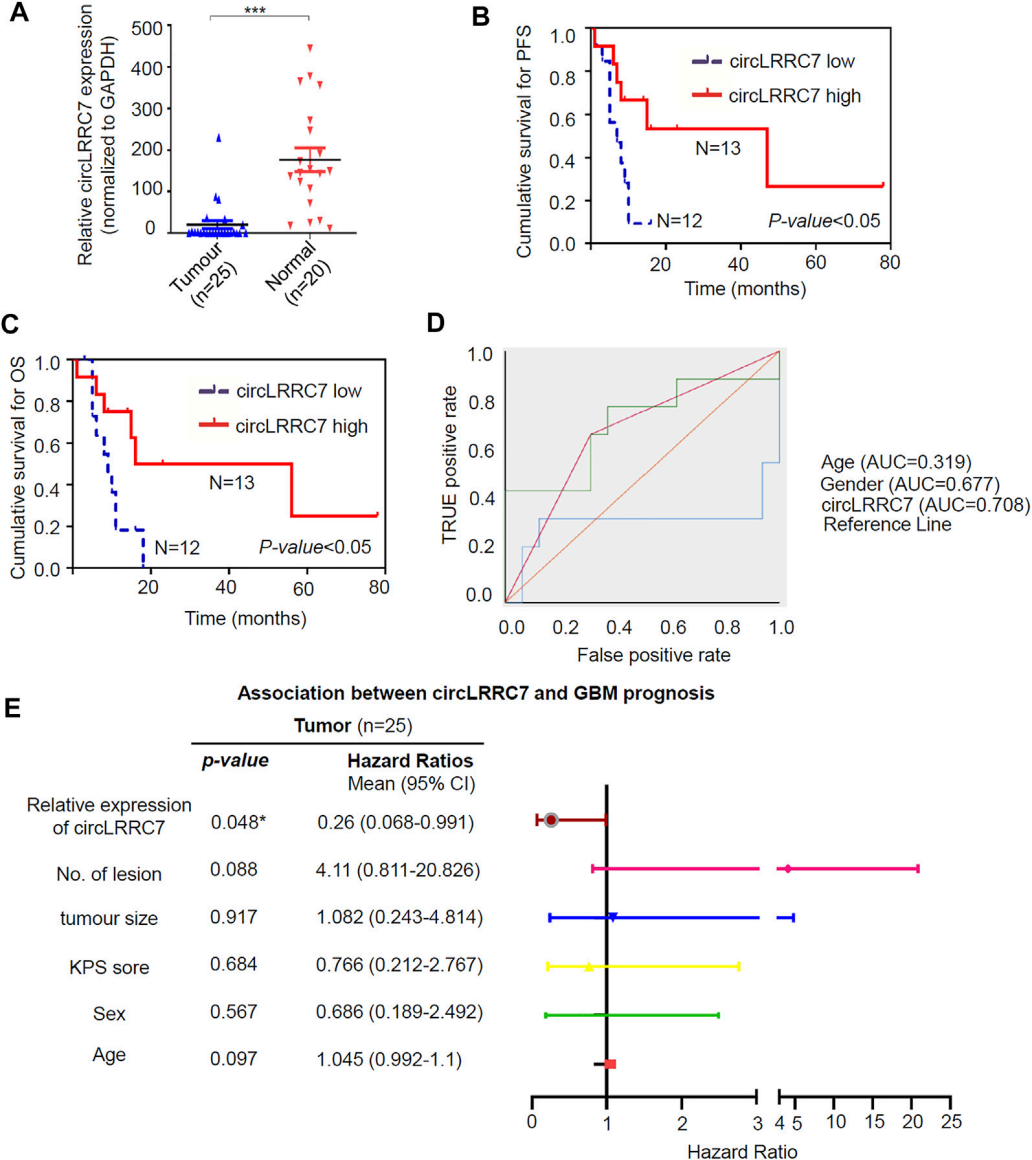
Validation of circLRRC7 as an independent prognostic predictor of GBM. **(A)** The relative expression of circLRRC7 in 20 normal brain tissues and 25 GBM tissues. **(B,C)** Kaplan-Meier PFS **(B)** and OS **(C)** curves of GBM patients in correlation with circLRRC7 expression. **(D)** ROC to predict prognosis based on clinicopathology characters. Only circLRRC7 expression showed diagnostic discriminatory value as the area under the ROC curve (AUC) was 0.708 **(E)** Forest map indicated that confidence interval (CI) of circLRRC7 was <1, but CI of number of lesions, tumour size, KPS score, sex and age stretched across 1. (*p < 0.05; ***p < 0.001).

More samples (25 GBM and 20 normal brain tissue from epilepsy patients) from our hospital were used to examine the expression of circLRRC7 and its association with clinicopathologic characteristics. Indeed, circLRRC7 was strongly downregulated in GBM (relative fold change> 7, *p* < 0.0001) (Figure 2A). When the GBM patients were stratified into the circLRRC7-high and circLRRC7-low groups by using the median circLRRC7 level as the cut-off, no significant relationship was identified between circLRRC7 expression and the age, gender, tumor size, KPS score, tumor location or IDH mutation status of the patients (Table 3). In contrast, higher circLRRC7 expression was strongly associated with better 1-year progressive-free survival (PFS, *p* = 0.011) and 1-year overall survival (OS, *p* = 0.011) (Table 3). Consistently, Kaplan-Meier survival analysis showed that higher circLRRC7 expression was significantly correlated with better PFS (median: high-19.4 m vs low-6.5 m, *p* < 0.05) and OS (median: high-20.9 m vs low-8.5m, *p* < 0.05) of the GBM patients (Figures 2B,C), suggesting that circLRRC7 may function to alleviate GBM progression. To determine the values of different clinicopathological characteristics (age, gender and circLRRC7 expression) on predicting prognosis, the ROC curves were plotted and the area under the ROC curve (AUC) larger than 0.7 was regarded as the threshold of a diagnostic discriminatory value. Only circLRRC7 expression showed and AUC of 0.708, indicating its capacity to predict prognosis of GBM patients (Figure 2D). To further confirm the impact of circLRRC7 on prognosis, the multivariate Meta-analysis was performed and the result indicated that the relative expression of circLRRC7 was a protective factor for the survival of GBM patients, whereas other factors such as number of lesions, tumour size, KPS score, sex and age did not affect prognosis (Figure 2E). In summary, circLRRC7 is the most significantly down-regulated circRNA in GBM with potential prognostic values.

**TABLE 3:** Clinical characteristics of GBM patients with different circLRRC7 expression.

Variable	Relative expression of circLRRC7 [Number (% of total)]		
	High	Low	*p*-value
Age year	—	—	—
<55	6 (24)	5 (20)	0.695
≥55	6 (24)	8 (32)	—
Gender	—	—	—
Male	7 (28)	5 (20)	
Female	5 (12)	8 (32)	0.434
Tumour Size	—	—	—
<5	7 (28)	7 (28)	—
≥5	5 (20)	6 (24)	1.000
KPS score	—	—	—
<80	2 (8)	4 (20)	1.000
≥80	10 (40)	9 (36)	—
Location	—	—	—
Frontal	2 (8)	6 (24)	0.293
Temporal	3 (12)	2 (8)	—
Insula	1 (4)	0 (0)	—
Central region	2 (8)	1 (4)	—
Multifoci	4 (16)	4 (16)	—
IDH	—	—	—
WT	10 (40)	13 (52)	0.220
Mutant	2 (8)	0 (0)	—
PFS	—	—	—
<1 year	5 (20)	12 (48)	0.011*
≥1 year	7 (28)	1 (4)	—
OS	—	—	—
<1 year	5 (20)	12 (48)	0.011*
≥1 year	7 (28)	1 (4)	—

Asterisks indicate statistical significance.

*p ≤ 0.05.

### Characterization of circLRRC7

**FIGURE 3:**
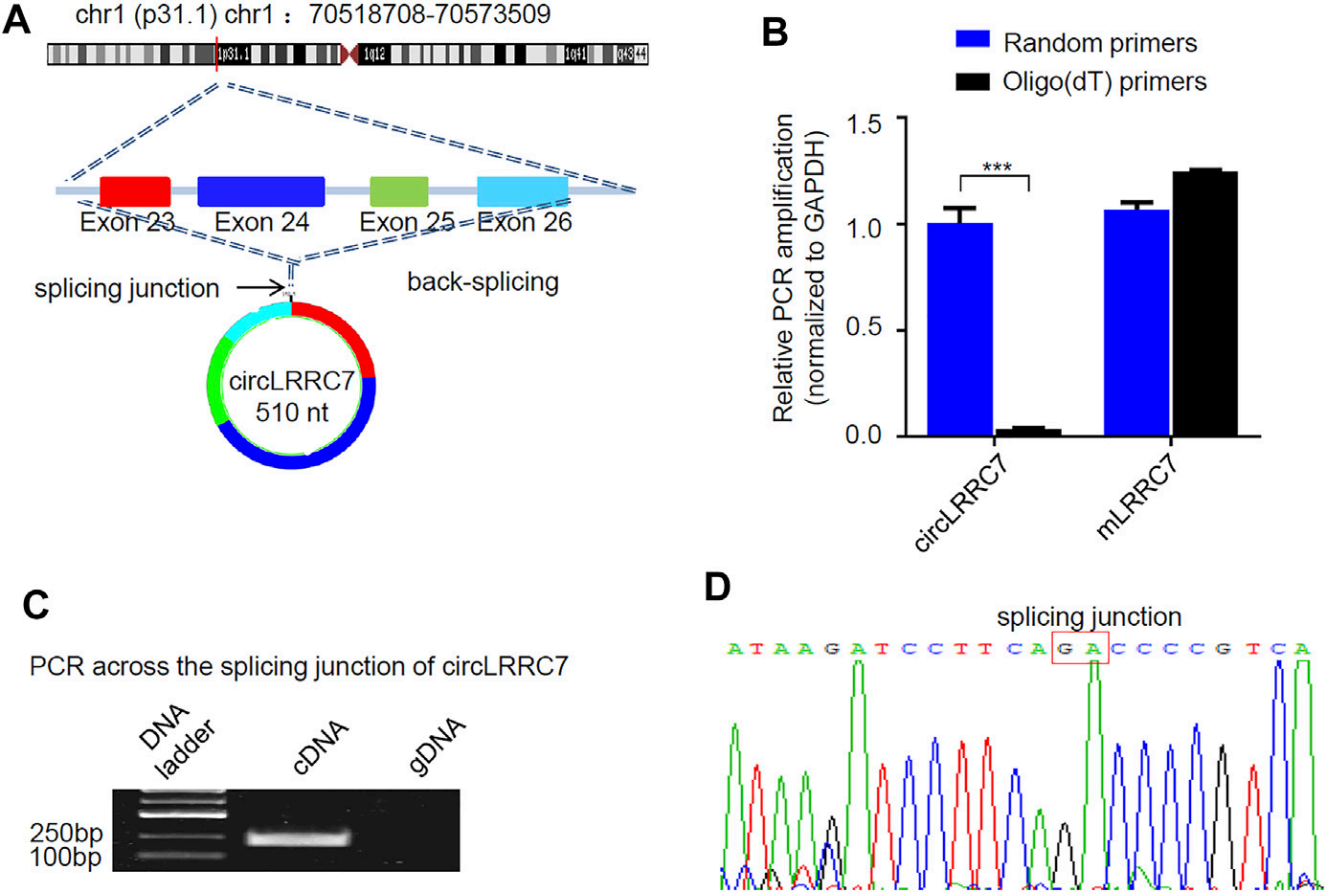
Characterization of circLRRC7. **(A)** The genomic localization of circLRRC7 in the LRRC gene. **(B)** Random hexamer and Oligo (dT) primers were used for the reverse transcription followed by qPCR. Oligo (dT) specifically transcribed mRNA, whereas random hexamer transcribed total RNAs. circLRRC7 was amplified from total RNA template but not mRNA template, whereas the host mLRRC7 was amplified from both total RNA and mRNA templates. **(C)** PCR across the splicing junction of circLRRC7 by using genomic DNA (gDNA) or cDNA as template to amplify circLRRC7. circLRRC7 was amplified from cDNA but not gDNA. **(D)** Sanger sequencing of the circLRRC7 PCR product. Red box represents splicing junction of circLRRC7. (***p < 0.001).

RNA-seq data showed that circLRRC7 is derived from the exons 23–26 of the LRRC7 gene and is transcribed into a 510nt circular RNA transcript (hsa_circ_0114014) in GBM (Figure 3A). We used forward primer for exon 26 and reverse primer for exon 23 of the LRRC7 gene to perform PCR analysis. The band of circLRRC7 was only detected by PCR using cDNA template transcribed with random Hexamer primers but not the oligo (dT) primers (Figure 3B), which confirmed the existence of circLRRC7 as a novel circular RNA. PCR using the genomic DNA (gDNA) as template did not generate the band of circLRRC7 (Figure 3C). Sanger sequencing of the PCR product confirmed the splice junction connecting exon 23 and exon 26 of the LRRC7 gene (Figure 3D). These results confirmed the circLRRC7 as a circular RNA transcribed from the LRRC7 gene in GBM.

### Identification of miR-1281 as a Potential Target of circLRRC7 in GBM

**FIGURE 4:**
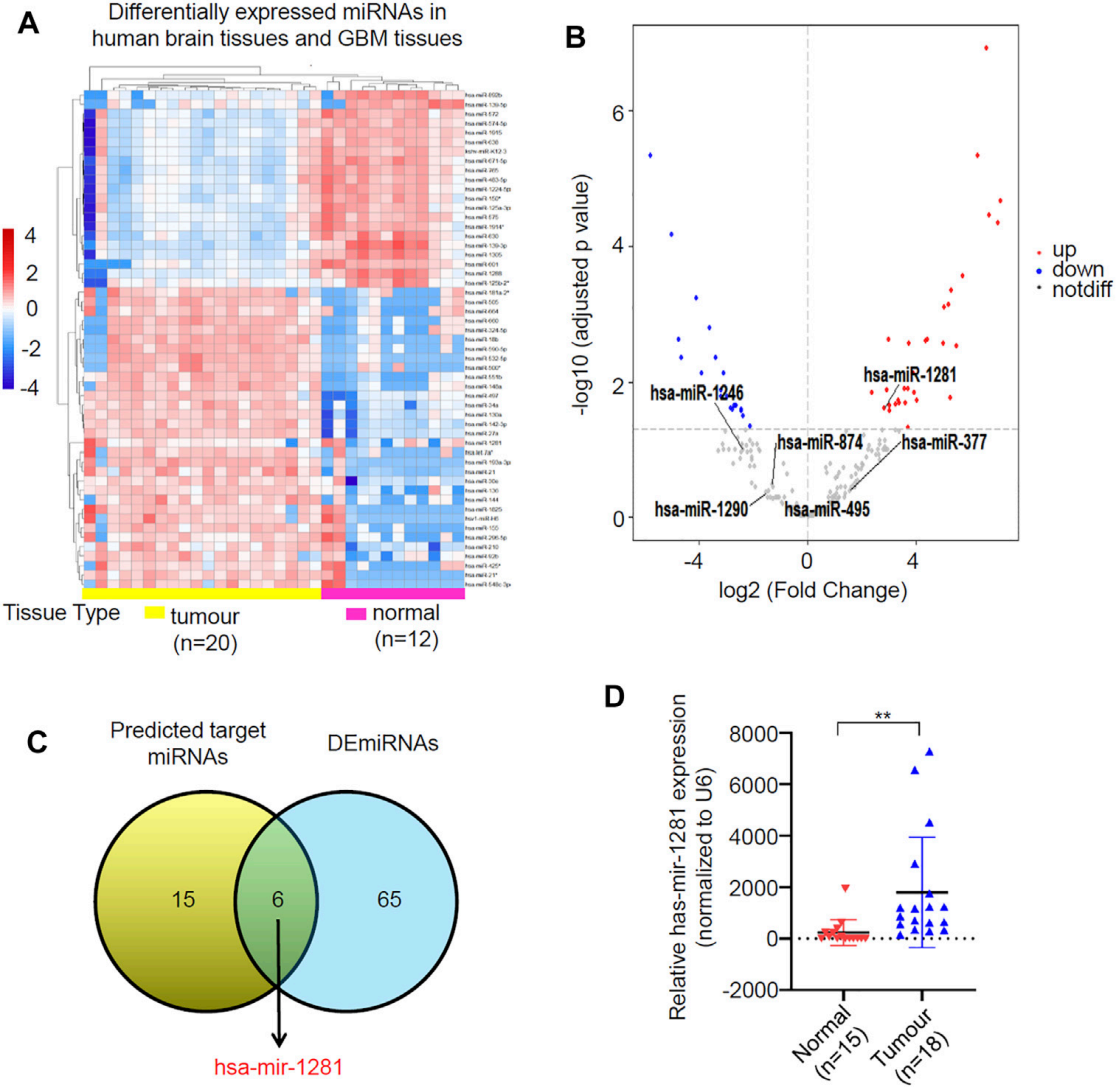
Identification of circRNAs-miRNAs axis. **(A)** Clustered heatmap showed profile of the differential expressed miRNAs in human brain tissues and GBM tissues. The color scale indicates the foldchange of the log2 value of the reads for each gene in tumors vs. normal tissues (red represents up-regulation; blue represents down-regulation). **(B)** Volcano plot of deferentially expressed miRNAs in GBM relative to normal brain tissues. The red dots represent upregulated miRNAs and blue dots represent downregulated miRNAs with statistical significance (|log2FC > 1 and *p*-value <0.05). **(C)** Identification of miR-1281 as the potential target miRNA of circLRRC7. **(D)** qPCR analysis of the expression levels of has-miR-1281 in normal and GBM tissue samples collected in our hospital. (**p < 0.01).

As most circRNAs function as miRNA sponges in tumors, we predicted target miRNAs of circLRRC7 by using the CSCD database and the CircInteractome database, resulting in 21 common miRNAs in the two databases (Supplementary Table S2). After then, we screened for differentially expressed miRNAs (DEmiRNAs) in adult GBM and normal brain tissues by using GEO dataset (GSE158284) that determined the most differentially expressed miRNAs in 20 GBMs and 12 normal brain tissues (Figure 4A), resulting in 41 upregulated and 30 downregulated DEmiRNAs (Figure 4B; Supplementary Table S3). Intersection of the 21 potential target miRNAs of circLRRC7 and the 71 DEmiRNAs revealed hsa-miR-1281 as the only common miRNA with significant upregulation in GBM (Figure 4C). Furthermore, we determined the expression levels of miR-1281 in a batch of normal (*n* = 15) and GBM (*n* = 18) tissues collected in our hospital. The results showed that miR-1281 was highly expressed in tumors relative to normal tissues (Figure 4D). Therefore, hsa-miR-1281 might be the potential target miRNA of circLRRC7 in GBM.

### Analysis of Potential Target Genes of miR-1281 in GBM

**FIGURE 5:**
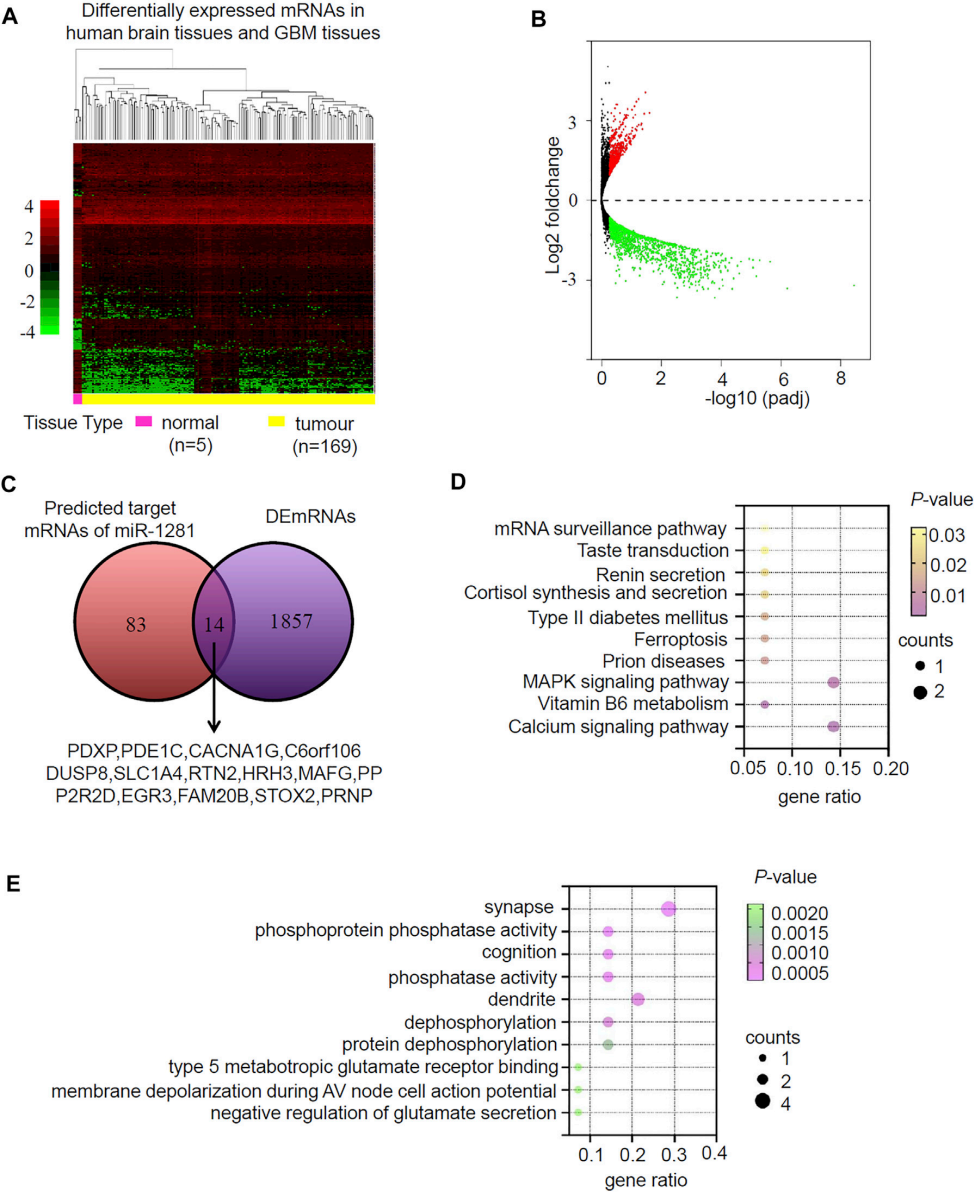
Prediction and analysis for Target genes of miRNA in GBM. **(A)** Clustered heatmap showing differentially expressed mRNAs (DEmRNAs) in human normal brain and GBM tissues. The color scale indicates the foldchange of the log2 value of the reads for each gene in tumors vs. normal tissues (red represents up-regulation; blue represents down-regulation). **(B)** Volcano plot of deferentially expressed miRNAs in human normal brain and GBM tissues. The red dots represent upregulated miRNAs and blue dots represent downregulated miRNAs with statistical significance (|log2FC > 1 and *p*-value <0.05). **(C)** Intersection of the predicted target mRNAs of miR-1281 and DEmRNAs. **(D)** The KEGG pathways in which the potential miR-1281 target mRNAs were enriched. **(E)** The GO analysis revealing the biological functions in which the potential miR-1281 target mRNAs were enriched.

miRNAs usually function by negatively regulating the expression of their target genes. We sought to identify the target mRNAs of hsa-miR-1281 via three databases (miRDB, miRTarBase and TargetScan) and found 97 candidate mRNAs (Supplementary Table S4). We then looked for differentially expressed mRNAs (DEmRNAs) in GBM relative to normal brain by analyzing the TCGA RNA-seq database (Supplementary Table S5), and found 1872 downregulated as well as 826 upregulated DEmRNAs (Figures 5A,B). Intersection of the 97 candidate mRNAs and the downregulated DEmRNAs resulted in 14 overlapping mRNAs (PDXP, PDE1C, CACNA1G, C6orf106, DUSP8, SLC1A4, RTN2HRH3, MAFG, PPP2R2D, EGR3, FAM20B, STOX2, PRNP) (Figure 5C). KEGG pathway analysis demonstrated that these genes were significantly enriched in calcium signaling pathway, vitamin B6 metabolism, MAPK signaling pathway and so on (Figure 5D). In GO analysis, these genes were annotated to certain biological processes, cellular components and molecular functions, such as synapse, phosphoprotein phosphatase activity, cognition, phosphatase activity, dendrite, and dephosphorylation (Figure 5E). Therefore, miR-1281 as a potential target of circLRRC7 may be involved in regulation of several signaling pathways in GBM.

### The Association Between circLRRC7, miR-1281, and PDXP in GBM

**FIGURE 6:**
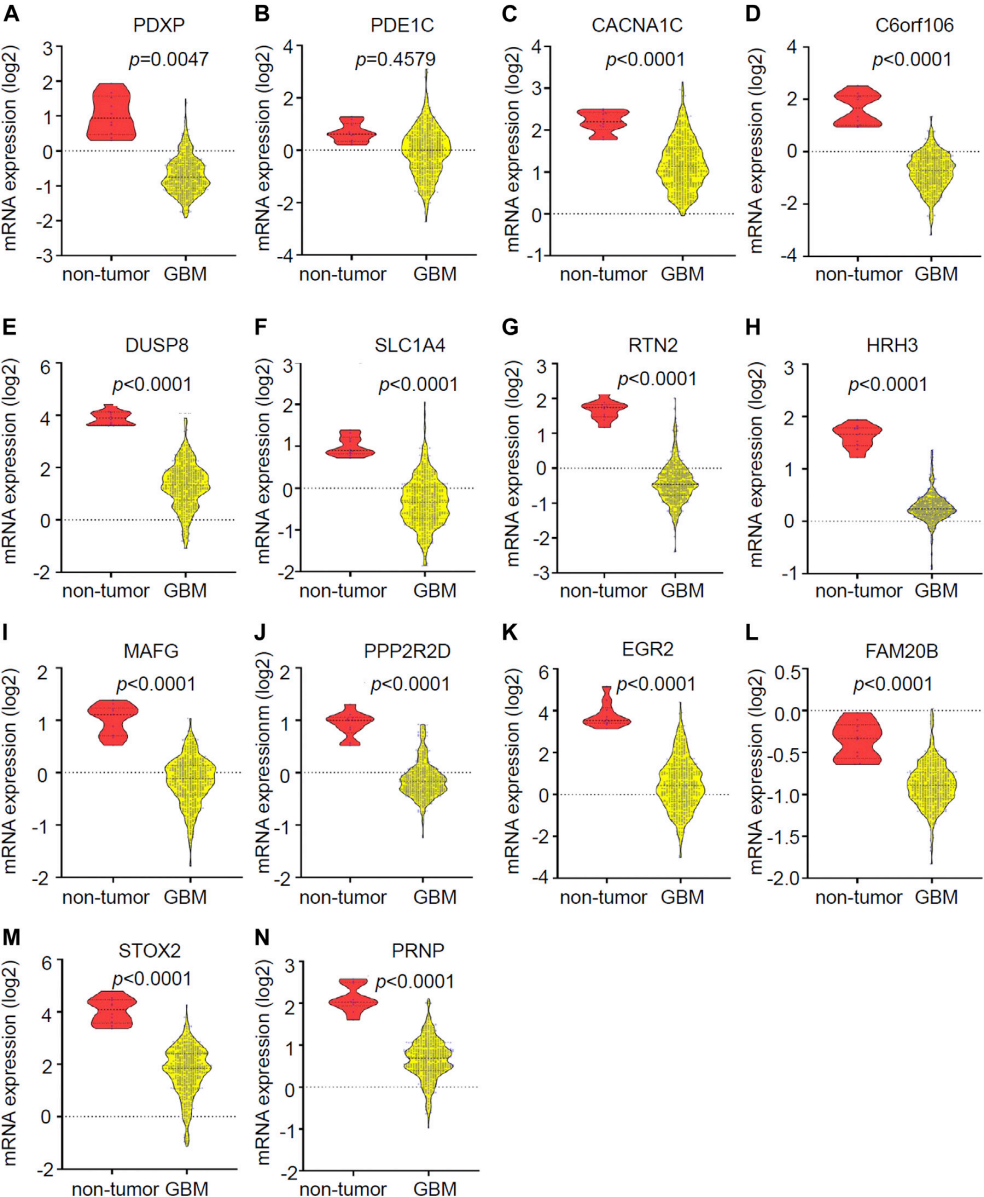
Validation of the expression of 14 potential downregulated genes in GBMs vs. brains. **(A–N)** Expression of **(A)** PDXP, **(B)** PDE1C, **(C)** CACNA1G, **(D)** C6orf106, **(E)** DUSP8, **(F)** SLC1A4, **(G)** RTN2, **(H)** HRH3, **(I)** MAFG, **(J)** PPP2R2D, **(K)** EGR3, **(L)** FAM20B, **(M)** STOX2, and **(N)** PRNP in GBM vs. brain tissues according to the Gliovis database. *p*-value < 0.05 was considered as statistically significant.

**FIGURE 7:**
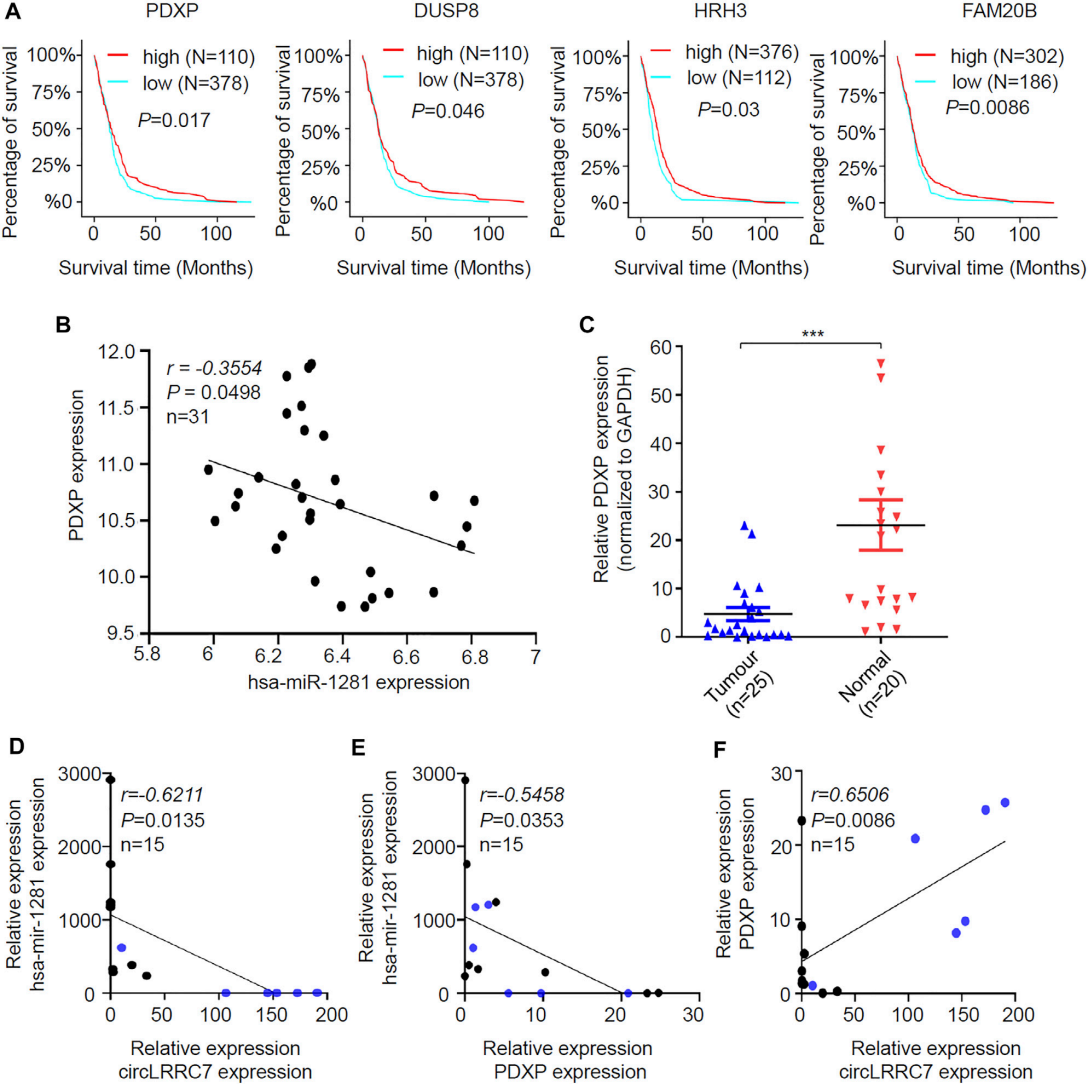
Identification of the potential circLRRC7-miR-1281-PDXP axis in GBM. **(A)** The impact of PDXP, DUSP8, HRH3, and FAM20B on the prognosis of GBM patients. **(B)** Correlation analysis of the Gliovis database revealing the negative correlation between miR-1281 and PDXP in GBM and brain tissues. **(C)** qPCR analysis of the PDXP levels in GBM and brain tissues collected in our hospital. **(D)** Lineage regression analysis for the correlation between circLRRC7 and miR-1281 in 15 brain and GBM tissues. circLRRC7 levels were negatively correlated to miR-1281. **(E)** Lineage regression analysis for the correlation between miR-1281 and PDXP in 15 brain and GBM tissues. circLRRC7 levels were negatively correlated to miR-1281. **(F)** Lineage regression analysis for the correlation between circLRRC7 and PDXP in 15 brain and GBM tissues. circLRRC7 levels were positively correlated to PDXP. Blue dots represent normal tissues and black dots represent tumor tissues. (***p < 0.001).

The expression of the 14 candidate miR-1281 target genes in GBM was further checked by analyzing the datasets of TCGA Agilent 4502-A platform microarray assays. The results showed that 13 genes except for PDE1C were lowly expressed in GBM relative to normal brains with statistical significance (Figure 6A-N). In addition, Kaplan-Meier survival analyses demonstrated that higher expression of PDXP, DUSP8, HRH3, and FAM20B was associated with better OS in GBM patients (Figure 7A). CircRNAs as ceRNAs may function as miRNA sponges to offset miRNA-mediated mRNA inhibition. By using the Gliovis database, we found that PDXP among the four candidates was the only gene with a negative correlation with miR-1281 (Figure 7B). By using the same batch of samples as in Figure 2A, we determined the expression levels of PDXP in normal and GBM tumor samples. The results showed that PDXP had a lower expression in tumor relative to normal tissues (Figure 7C). We further tried to determine the potential association between circLRRC7, miR-1281 and PDXP in the samples collected in our hospital. Among all samples collected in our hospital at different time points, simultaneous detection of circLRRC7, miR-1281 and PDXP was achieved in 15 samples. Lineage regression analyses revealed a negative correlation between circLRRC7 and miR-1281 (Figure 7D). Meanwhile, a negative correlation was detected between miR-1281 and PDXP (Figure 7E). However, a positive correlation was detected between circLRRC7 and PDXP in these samples (Figure 7F). These data strongly suggest the association between circLRRC7, miR-1281 and PDXP in GBM progression.

## Discussion

Despite the advances in GBM treatment in the past decades, GBM is still a disease with a very high fatality rate (Okolie et al., 2016). GBM tumor cells demonstrate rapid proliferation and infiltrative growth, but the underlying mechanisms are yet unclear. Increasing evidence has indicated that circRNAs play important roles in physiological and pathophysiological processes in the central nervous system (Li et al., 2019). As the malignant brain tumor, GBM may have altered circRNA expression that may affect tumor development and progression. According to the cancer circRNA landscape through exome capture transcriptome sequencing, circRNAs may have diagnostic and therapeutic values in glioma (Vo et al., 2019). Nevertheless, there are very limited studies about the functions and mechanisms of circRNAs as tumor suppressors. In this study, by systematic analysis of existing databases, we detected circLRRC7 as a tumor suppressor and proposed miR-1281 and PDXP as potential downstream genes of circLRRC7 in GBM pathogenesis. High expression of circLRRC7 was significantly associated with favorable PFS and OS in GBM patients. Moreover, meta-analysis revealed that circLRRC7 was associated with prognosis of GBM in our study (Figure 2E). The tumor size and age showed no connection with prognosis (Figure 2E) probably due to the limited number of samples. More specimens are required for future studies about the impact of circLRRC7 on the proliferation, apoptosis and invasiveness of GBM cells.

LRRC7, the host gene of circLRRC7, is a major component of the post-synaptic density (PSD) of excitatory synapses (Liu et al., 2013), and is reported to be associated with memory formation (Kim et al., 2020). We did not detect significantly altered LRRC7 expression in GBM relative to normal brain tissues in the Gliovis database. However, our results showed that circLRRC7 derived from LRRC7 was distinctively downregulated in GBM tissues compared to normal brain samples. As circRNAs were derived from pre-mRNAs (Ashwal-Fluss et al., 2014), we predicted that splicing factors may participate in circLRRC7 formation, but the detailed molecular mechanisms underlying the downregulation of circLRRC7 in GBM required further exploration.

Plenty of evidence has shown that circRNAs regulate the expression of target miRNAs by acting as molecular sponges (Zhong et al., 2016). We identified has-miR-1281 as a potential target miRNA of circLRRC7. Hsa-miR-1281 was reported to be negatively regulated by linc-GIHCG in gastric cancer (Liu et al., 2019) and breast cancer (Fan et al., 2019). Hsa-miR-1281 was also found as a p53-responsive microRNA that targeted USP39 to inhibit the survival of human osteosarcoma cells under ER stress (Jiang et al., 2018). Although these previous studies demonstrated the down-regulation of miR-1281 in breast and gastric cancers, we detected the upregulation of miR-1281 in GBM samples, indicating the different roles of miR-1281 in different tumors. Such difference may rely on the specific downstream target of microRNAs. We predicted PDXP as a potential downstream target of miR-1281 in GBM, which was supported by the fact that the expressions of PDXP and miR-1281 were negatively correlated. PDXP had never been associated with miR-1281 in other cancers, and the tumorigenic functions of miR-1281 in GBMs may rely on PDXP.

Through systematic analysis of a series of databases, we connected circLRRC7 to has-MIR-1281 and PDXP in GBM. Previous studies reported that monoallelic PDXP (Pyridoxal phosphate phosphatase) loss frequently occurred in gliomas and the occurrence rate increased along with glioma grades. GBM patients with lower median PDXP expression levels had significantly shorter mean survival periods (Schulze et al., 2016). In addition, PDXP regulated active vitamin B6 levels and transcriptomic features of GBM cell lines cultured under non-adherent, serum-free conditions (Schulze et al., 2018), indicating a potential role of PDXP in tumor metabolism and cancer stem cells. Future studies will further unveil the roles of circLRRC7, miR-1281, and PDXP in the development of GBM and verify the prognostic values of these genes in GBM patients.

## Data Availability

The original contributions presented in the study are included in the article/Supplementary Material, further inquiries can be directed to the corresponding authors.
