# Supplementary material for: CircularLRRC7 is a Potential Tumor Suppressor Associated With miR-1281 and PDXP Expression in Glioblastoma
Source: Front Mol Biosci. 2021 Nov 29;8:743417. doi: 10.3389/fmolb.2021.743417 (PMC8667166; doi:10.3389/fmolb.2021.743417)
Supplement: Supplementary file 1 [file Table1.DOCX]

**Table1** the differentially expressed circRNAs with significance（ |logFC| > 1，padj<0.05）between normal brain and gliomas tissues

| **circRNA name** | ***P*-value** | **B** | **Log_2_FC** |
| --- | --- | --- | --- |
| hsa_circ_0073237  hsa_circ_0069865  hsa_circ_0024085  hsa_circ_0000437  hsa_circ_0009027  hsa_circ_0021350  hsa_circ_0006988  hsa_circ_0126761  hsa_circ_0003718  hsa_circ_0007848  hsa_circ_0000643  hsa_circ_0000419  hsa_circ_0007513  hsa_circ_0001136  hsa_circ_0001400  hsa_circ_0005692  hsa_circ_0095454  hsa_circ_0006618  hsa_circ_0004674  hsa_circ_0000524  hsa_circ_0004777  hsa_circ_0001359  hsa_circ_0006944  hsa_circ_0001727  hsa_circ_0001004  hsa_circ_0008587  hsa_circ_0001897  hsa_circ_0004086  hsa_circ_0006127  hsa_circ_0007292  hsa_circ_0006677  hsa_circ_0005823  hsa_circ_0004592  hsa_circ_0002468  hsa_circ_0138300  hsa_circ_0001810  hsa_circ_0000258  hsa_circ_0121507  hsa_circ_0131934  hsa_circ_0001681  hsa_circ_0078784  hsa_circ_0001368  hsa_circ_0055954  hsa_circ_0017184  hsa_circ_0008278  hsa_circ_0003028  hsa_circ_0000348  hsa_circ_0111832  hsa_circ_0002904  hsa_circ_0104727  hsa_circ_0000296  hsa_circ_0005054  hsa_circ_0005114  hsa_circ_0000118  hsa_circ_0035796  hsa_circ_0131933  hsa_circ_0047270  hsa_circ_0003915  hsa_circ_0074362  hsa_circ_0001829  hsa_circ_0139669  hsa_circ_0001640  hsa_circ_0001095  hsa_circ_0080941  hsa_circ_0024997  hsa_circ_0064615  hsa_circ_0010467  hsa_circ_0098514  hsa_circ_0114014  hsa_circ_0003068  hsa_circ_0001558  hsa_circ_0132023  hsa_circ_0120050  hsa_circ_0132020  hsa_circ_0092765  hsa_circ_0002544  hsa_circ_0138460  hsa_circ_0073128  hsa_circ_0018403  hsa_circ_0077837  hsa_circ_0076948  hsa_circ_0135915  hsa_circ_0000119  hsa_circ_0000219  hsa_circ_0008215  hsa_circ_0006935  hsa_circ_0132022  hsa_circ_0016351  hsa_circ_0137676  hsa_circ_0099549  hsa_circ_0092798  hsa_circ_0138744  hsa_circ_0007294  hsa_circ_0064618  hsa_circ_0001392  hsa_circ_0002584  hsa_circ_0019061  hsa_circ_0125987  hsa_circ_0079557  hsa_circ_0103896  hsa_circ_0134739  hsa_circ_0007162  hsa_circ_0071375  hsa_circ_0018401  hsa_circ_0000117  hsa_circ_0139566  hsa_circ_0095626  hsa_circ_0114304  hsa_circ_0053441  hsa_circ_0117770  hsa_circ_0001661  hsa_circ_0003045  hsa_circ_0007062  hsa_circ_0097878  hsa_circ_0133954  hsa_circ_0001367  hsa_circ_0007132  hsa_circ_0042049  hsa_circ_0098551  hsa_circ_0008599  hsa_circ_0073379  hsa_circ_0125149  hsa_circ_0007242  hsa_circ_0001369  hsa_circ_0009009  hsa_circ_0103284  hsa_circ_0103204  hsa_circ_0001535  hsa_circ_0008199  hsa_circ_0078619  hsa_circ_0129941  hsa_circ_0079422  hsa_circ_0006667  hsa_circ_0006365  hsa_circ_0052318  hsa_circ_0002103  hsa_circ_0003171  hsa_circ_0088300  hsa_circ_0001868  hsa_circ_0001467  hsa_circ_0074368  hsa_circ_0004083  hsa_circ_0127310  hsa_circ_0099295  hsa_circ_0005164  hsa_circ_0085302  hsa_circ_0004087  hsa_circ_0007843  hsa_circ_0115525  hsa_circ_0132250  hsa_circ_0002972  hsa_circ_0006561  hsa_circ_0121603  hsa_circ_0073517  hsa_circ_0007108  hsa_circ_0001519  hsa_circ_0130789  hsa_circ_0007042  hsa_circ_0002702  hsa_circ_0096736  hsa_circ_0001246  hsa_circ_0006916  hsa_circ_0001615  hsa_circ_0108703  hsa_circ_0124264  hsa_circ_0005077  hsa_circ_0137709  hsa_circ_0139117  hsa_circ_0003101  hsa_circ_0024843  hsa_circ_0000615  hsa_circ_0006034  hsa_circ_0107086  hsa_circ_0139737  hsa_circ_0005882  hsa_circ_0099755  hsa_circ_0005540  hsa_circ_0075158  hsa_circ_0000371  hsa_circ_0000198  hsa_circ_0125610  hsa_circ_0097876  hsa_circ_0099456  hsa_circ_0138458  hsa_circ_0130363  hsa_circ_0057553  hsa_circ_0129477  hsa_circ_0064555  hsa_circ_0136694  hsa_circ_0003341  hsa_circ_0100397  hsa_circ_0042079  hsa_circ_0005003  hsa_circ_0125143  hsa_circ_0000620  hsa_circ_0105987  hsa_circ_0007723  hsa_circ_0056567  hsa_circ_0008928  hsa_circ_0072654  hsa_circ_0002484  hsa_circ_0017848  hsa_circ_0005314  hsa_circ_0004849  hsa_circ_0009576  hsa_circ_0005037  hsa_circ_0024999  hsa_circ_0003279  hsa_circ_0078328  hsa_circ_0002546  hsa_circ_0134899  hsa_circ_0005646  hsa_circ_0110608  hsa_circ_0000994  hsa_circ_0025612  hsa_circ_0001882  hsa_circ_0005335  hsa_circ_0130887  hsa_circ_0130236  hsa_circ_0006107  hsa_circ_0000002  hsa_circ_0002867  hsa_circ_0002163  hsa_circ_0132837 | 1.88E-32  4.03E-09  1.51E-06  1.51E-06  6.85E-05  0.000550079  0.000590814  0.000601194  0.000721901  0.001797076  0.001836994  0.001973507  0.002405358  0.003067599  0.006105032  0.00628762  0.006536969  0.006635828  0.006724457  0.007318631  0.008308  0.008691715  0.010069257  0.015304083  0.015409684  0.015959265  0.017270541  0.017825163  0.017844604  0.017990837  0.018322382  0.019311905  0.021950012  0.022467331  0.025748721  0.027042818  0.028590271  0.044147586  2.69E-16  4.83E-10  2.97E-09  1.26E-08  2.34E-08  8.20E-07  1.51E-06  1.51E-06  1.57E-06  2.43E-06  4.95E-06  4.95E-06  1.20E-05  1.20E-05  1.92E-05  1.92E-05  6.10E-05  6.11E-05  6.25E-05  7.86E-05  8.34E-05  8.34E-05  8.47E-05  9.14E-05  9.14E-05  0.00013059  0.000135288  0.000177874  0.000191653  0.000277952  0.000277952  0.000330471  0.000371599  0.000391518  0.000413924  0.000501027  0.000507602  0.000546439  0.000546439  0.000829692  0.000863374  0.000996844  0.001145622  0.001424485  0.001486111  0.001670161  0.001670161  0.001670161  0.001836994  0.001973507  0.002070687  0.002073456  0.002167796  0.002167796  0.002225962  0.002225962  0.002225962  0.002225962  0.002405358  0.002405358  0.002586049  0.002912382  0.002950023  0.002984846  0.003130769  0.003406102  0.003720095  0.004298548  0.004672625  0.004812238  0.004812238  0.004812238  0.005002128  0.005140825  0.005140825  0.005713078  0.00607934  0.00607934  0.006105032  0.00628762  0.00628762  0.00628762  0.006465345  0.006623501  0.006623501  0.006635828  0.006635828  0.006947795  0.006950237  0.007014792  0.007014792  0.007162766  0.007162766  0.007199606  0.007199606  0.007295447  0.007606697  0.007606697  0.007648635  0.008240376  0.009347666  0.00951782  0.010459416  0.010459416  0.010459416  0.010627904  0.010875426  0.011150338  0.0118963  0.012374954  0.013773657  0.01400692  0.01400692  0.014924987  0.015409684  0.016085648  0.017052524  0.017270541  0.017270541  0.017270541  0.017270541  0.017270541  0.017825163  0.017825163  0.018322382  0.018322382  0.018322382  0.018616856  0.01876246  0.01953968  0.021023176  0.021464585  0.022467331  0.02426992  0.025257146  0.025404542  0.025404542  0.025676928  0.025676928  0.025748721  0.026118265  0.026923623  0.027343653  0.027400778  0.028557957  0.028590271  0.028917424  0.028983295  0.02915259  0.029413529  0.029605219  0.030379003  0.030663117  0.030663117  0.030663117  0.030663117  0.030663117  0.030910975  0.031109991  0.031674311  0.032081793  0.033113498  0.036441188  0.037273637  0.037273637  0.037273637  0.037273637  0.037273637  0.038376576  0.039628466  0.039628466  0.039883762  0.040445008  0.042829683  0.044147586  0.044147586  0.044446599  0.044973205  0.045868686  0.047378099  0.047378099  0.047378099  0.047378099  0.047378099  0.048410402  0.048851158 | 105.0081668  9.400758999  3.615830329  21.98412133  3.750267651  1.227686718  4.637276201  5.991652322  6.361446281  3.592610294  6.224263975  5.050903237  1.411585086  8.448047683  4.673799721  1.414534  1.967716006  1.254799336  2.350912611  6.419718666  1.288933724  2.590435906  2.862353204  11.51991655  4.737908313  6.006835684  5.817649398  1.080286942  1.539573724  13.54694661  10.94123519  1.919657138  1.285280556  1.483366341  2.981494638  1.076791926  1.291085434  1.201349239  5.220663592  7.173432769  5.253227992  11.58790123  4.342061264  13.26839558  6.265156047  14.53543145  23.92475038  3.111206153  3.880464848  2.695913999  27.25921318  4.567002793  15.14708395  54.15487613  11.93344726  9.621884336  5.104256619  7.137742483  3.01533954  11.22845619  2.749673571  2.641763371  6.530721325  2.20207595  3.635645675  5.461329861  3.292399001  2.110746785  1.699945287  1.438251854  11.94530638  13.74220403  4.623300924  2.03146529  11.31433496  5.615232587  1.816488947  2.393169586  1.721372667  3.704013143  21.06658607  1.157582003  17.27960106  5.481319412  1.626579191  1.672024842  2.050578162  1.735731281  1.176755136  1.581556519  1.603328349  5.513926722  5.799914305  1.929773281  7.841714266  2.306368655  1.941217947  6.097431993  2.646655039  5.594913055  3.742380894  1.907186719  2.001062902  4.140063534  11.80831499  8.040493383  1.21814835  1.561727853  3.495536352  1.60805942  3.450687372  2.954327342  2.832753427  1.361671754  2.812368035  4.725842148  4.766508544  4.59947791  1.759881853  2.009698395  1.736436352  3.378274729  2.124001877  2.468106417  1.470995721  1.764445391  1.50396415  10.2093959  1.465093813  1.211884318  1.257408089  3.165362396  4.944616968  3.309493135  2.61217789  3.641721388  7.928795431  1.680696251  2.767119996  7.548967034  3.394336238  1.738827545  1.692817432  1.909176061  3.742728814  2.517956454  6.882309137  3.170427789  8.332826538  29.06589052  3.751938849  0.980543261  1.399672218  3.075569869  7.883575851  3.23732002  6.123191972  1.94521872  1.487574286  1.230904102  4.730559318  9.101213303  1.064603586  1.730679805  3.391492374  2.862340427  5.568579215  1.695498684  2.892343444  1.026947118  8.378455871  1.705107718  1.025904448  1.390731542  11.07557814  1.200917009  2.042792987  1.226987471  2.717015752  1.826062045  2.442687176  5.104755991  1.054000687  1.481473659  1.481784457  0.917129734  1.693095318  3.348085599  2.645064841  1.824318952  0.921632147  1.698157463  1.071726634  1.076800641  2.461245339  1.117007509  1.851281137  1.656284799  1.166148622  1.413181737  2.836995436  1.573502682  0.79990245  2.216930044  1.326082347  1.338642089  2.765932398  1.154087628  1.340147349  3.329411665  6.649287929  1.569268828  0.977541242  33.63766036  3.411157853  1.865908536  1.648917843  0.821421728  1.144576923  3.830526192  1.740016432  1.772304209  1.377456389  0.930457599 | 5.284232218  2.885349538  4.63877503  1.419134212  4.576516122  4.245986517  3.140096485  2.585565377  3.379469992  2.608283523  1.792951365  3.273966812  4.398741595  1.339423386  2.028685433  3.938678238  4.017808531  3.823914348  2.841853536  2.020659967  4.245209467  3.243154247  2.470086002  1.027002929  2.314962897  1.870776853  1.805750177  3.980530476  3.61066513  1.313487397  1.081097397  2.891739963  3.421877016  3.412359688  3.137180981  3.522952933  2.995363307  3.503545776  -5.616262636  -3.792374821  -3.96307385  -3.388961266  -4.47807931  -1.798686371  -2.965258265  -2.069008599  -1.449597962  -3.253827022  -3.126967834  -3.893202254  -1.16040558  -3.265333732  -2.26225217  -1.353047536  -1.8430666  -2.969114958  -2.344411367  -2.269590932  -2.966513366  -1.907207659  -3.577069062  -3.638953856  -2.403215134  -3.461895216  -2.51697262  -3.294739919  -2.587956787  -2.965051421  -3.987167056  -3.761630284  -2.000868204  -2.141366414  -2.374084062  -3.411576274  -2.547461483  -1.985803228  -3.277293666  -2.596622666  -3.117183024  -3.49083578  -1.031723309  -3.048009979  -1.536196996  -1.904352073  -3.376914922  -3.067535027  -3.390271901  -2.98001214  -3.461764892  -3.020130328  -3.913020024  -2.941236665  -2.238455578  -3.339144969  -1.781849305  -2.810046513  -2.889161937  -2.092132108  -3.156453408  -3.006814526  -3.131978521  -3.1397763  -2.973378554  -2.310351394  -1.138895203  -1.772035924  -2.959648615  -2.843882223  -2.716481375  -2.745514978  -1.941205793  -2.096209431  -2.071717365  -3.672169416  -2.861432147  -2.102187648  -2.019383495  -1.690862824  -3.038325381  -2.969009141  -2.404762891  -2.287255573  -2.664654271  -2.93751864  -2.650915806  -3.676268205  -2.995452841  -1.372216491  -3.784640487  -2.744683926  -3.163954152  -2.7421762  -2.6788032  -1.934922052  -2.191046567  -1.932744899  -1.311630931  -2.887191284  -1.855652809  -1.839230424  -1.819007283  -2.545835592  -2.55676915  -2.421733216  -2.186004752  -1.9172326  -1.270343701  -2.08039127  -1.371363252  -1.19590545  -1.655868797  -3.19781665  -3.161707231  -1.917158429  -1.224953285  -1.614485533  -1.624062645  -2.423822078  -2.598330476  -3.528400025  -1.434794205  -1.413300104  -3.322172813  -2.663636237  -1.97931298  -2.19101762  -1.519320098  -3.144254267  -2.04078185  -3.271782323  -1.240468796  -2.397904323  -2.69113797  -2.841708283  -1.259252765  -2.715230966  -2.266287176  -2.710902341  -1.975388819  -2.147423081  -2.27746719  -1.754052267  -3.312295856  -2.638145382  -3.412345134  -3.111122241  -2.13301733  -2.317756864  -2.081071658  -2.712355812  -3.109118306  -2.619475336  -2.764798605  -2.954691185  -2.231657299  -2.811729636  -2.487597374  -2.477223851  -2.648614131  -2.322413787  -2.184437016  -2.61791331  -2.906377697  -2.051039249  -2.718032168  -2.898403595  -2.036403583  -2.510011821  -2.555398931  -1.806921243  -1.879447493  -2.271538803  -3.206014947  -1.1033561  -1.570864032  -2.260177548  -2.577400035  -2.944061064  -2.805462419  -1.563358738  -2.602544373  -2.193144082  -2.972805033  -3.13336434 |
