# Supplementary material for: CircularLRRC7 is a Potential Tumor Suppressor Associated With miR-1281 and PDXP Expression in Glioblastoma
Source: Front Mol Biosci. 2021 Nov 29;8:743417. doi: 10.3389/fmolb.2021.743417 (PMC8667166; doi:10.3389/fmolb.2021.743417)
Supplement: Supplementary file 2 [file Table2.DOCX]

**Supplementary Table 2. The binding miRNAs of circRNAs predicted by CSCD and circular RNA interactome.**

| CircRNA ID | miRNA ID |
| --- | --- |
| hsa_circ_0114014  hsa_circ_0114014  hsa_circ_0114014  hsa_circ_0114014  hsa_circ_0114014  hsa_circ_0114014  hsa_circ_0114014  hsa_circ_0114014  hsa_circ_0114014  hsa_circ_0114014  hsa_circ_0114014  hsa_circ_0114014  hsa_circ_0114014  hsa_circ_0114014  hsa_circ_0114014  hsa_circ_0114014  hsa_circ_0114014  hsa_circ_0114014  hsa_circ_0114014  hsa_circ_0114014  hsa_circ_0114014 | hsa-miR-1203  hsa-miR-1229  hsa-miR-1246  hsa-miR-1248  hsa-miR-1252  hsa-miR-1261  hsa-miR-1281  hsa-miR-1287  hsa-miR-1290  hsa-miR-377  hsa-miR-409-3p  hsa-miR-485-3p  hsa-miR-495  hsa-miR-502-5p  hsa-miR-578  hsa-miR-604  hsa-miR-605  hsa-miR-628-3p  hsa-miR-647  hsa-miR-874  hsa-miR-938 |
