# Supplementary material for: CircularLRRC7 is a Potential Tumor Suppressor Associated With miR-1281 and PDXP Expression in Glioblastoma
Source: Front Mol Biosci. 2021 Nov 29;8:743417. doi: 10.3389/fmolb.2021.743417 (PMC8667166; doi:10.3389/fmolb.2021.743417)
Supplement: Supplementary file 3 [file Table5.DOCX]

**Supplementary Table 5. Identification of differentially expressed genes (DEGs) between GBM and normal brain tissues.**

| Gene | P-value | t | B | log2FoldChange |
| --- | --- | --- | --- | --- |
| SST  RASAL1  MAST3  DNAJC6  AK5  SNCA  SYNGR1  FAIM2  HSPA12A  PPP2R2C  PNMAL2  ICAM5  GABRA5  PWAR6  RAPGEF4  KIAA0513  NCDN  PRRT1  ADAM11  CTD-2562J17.6  KCNH3  ATP8A1  SLC8A2  CPNE6  MAP1A  MAGEE1  GPRASP1  SCN2B  NPTXR  NAPB  STX1B  MPPED1  SYP  KIF1A  ENC1  MAPK8IP2  CEP170B  SERPINI1  NCS1  PRKCG  JAKMIP1  STXBP1  GDA  SMIM10L2B  KCNAB2  KCNJ9  SYN2  PAK1  SULT4A1  NECAB1  PDE2A  NEURL1  DNM1  RASGRF1  CHD5  KCNJ4  SLC32A1  DOC2A  CHN1  KIF5C  SYNGR3  AMPH  NAP1L2  CACNG8  KNDC1  PRKAR1B  STX1A  SEPT5  PEX5L  SCN3B  RP11-320H14.1  PRKCZ  SCN2A  SYT5  TPPP  SV2B  MAP7D2  SVOP  SNAP91  RGS7  FAM163B  LONRF2  PTER  CASKIN1  SYT7  PDE1A  C1orf115  PRKCB  RAB11FIP4  PHF24  CLSTN3  SLC30A3  SLC17A7  SYT1  DMTN  ATP1B1  SOWAHA  RAB3A  GRIN1  EHD3  WBSCR17  PEG3  SPTBN2  CAMK2B  GLT1D1  EPHB6  BSN  TMEM130  MAL2  DLG2  GOT1  EEF1A2  PSD3  CELF4  CAMK1D  RTN1  NECAB2  NELL2  RUNDC3A  PTPN5  CLCN4  TEF  PPFIA3  PHYHIP  JPH3  FBXL16  INPP5F  BRINP1  KCNJ3  ATP1A3  FBXO41  PNCK  CA11  TMEM246  LYNX1  TMEM155  CHGB  NRIP3  SYN1  SLC6A17  NUAK1  RAP1GAP  TUBA4A  CREG2  RBFOX1  GAD2  KCNK1  SLC12A5  CACNB1  DOCK3  SH2D5  CPLX1  DLGAP3  NRGN  PSD  CNTNAP2  ASPHD1  GABRD  CACNG3  RAB3C  UNC13A  SLC4A10  KIAA1107  DLG4  NPTX1  SNAP25  CHRM1  MFSD4  ADAP1  MGAT3  SLC7A14  MIR770  PTPRN  FRRS1L  PPP1R16B  SNCG  ATL1  GABRB3  NSF  NMNAT2  ADRA1B  SLC1A2  GABARAPL1  RTN4R  RAP1GAP2  HPCAL4  HRH3  OLFM1  CAMKK1  PACSIN1  KIAA1644  CAMKV  CELF5  GABRA1  NGEF  CBX7  RAB40B  ST8SIA3  PI4KA  UNC5A  GABBR1  ATP6V1G2  ATP2B2  KIF3C  DUSP8  GRM3  BZRAP1  FBXO2  NRSN1  PRKCE  SNPH  SYT4  ARHGDIG  CALY  CEND1  ITPKA  VSNL1  CNKSR2  JPH4  PITPNM3  REPS2  SYNPR  IQSEC3  MT-TL1  TSPYL2  CPLX2  SMIM10L2A  GNAO1  NAP1L3  PAK3  HS6ST3  DNAJA4  HS3ST4  PPP3CB  RGS7BP  SHANK1  SH3GL3  RGS4  NRXN3  DDN  SNCB  CDK5R2  C1QL3  IDS  TMEM151B  SH3GL2  LINC00641  GABRG2  GLS  CDK5R1  EPB41L3  NEGR1  RP11-1263C18.1  OGDHL  C1orf95  BEX5  SRCIN1  CABP1  MAPK8IP3  CAMK2N1  CAMK2A  KIAA1549L  SLC25A27  VSTM2L  MEF2C  SYT13  PDZD4  C1QTNF4  RAB15  ANKS1B  STK32C  VAMP2  PRRT2  SLC24A2  ZCCHC12  SHANK3  MYO5A  GNG3  SEMA6B  C11orf87  IQSEC2  RAB6B  ADCY5  TSPYL1  RP11-701H24.3  PDE1B  EPB41L1  NPM2  VSTM2B  PTPRT  MGAT5B  RUSC2  SCAMP5  CHGA  PNMA3  KCNN1  SLC25A22  HPCA  CYFIP2  HLF  MTND4P12  RP13-514E23.1  MTURN  TAGLN3  DLGAP1  YWHAH  MATK  CHRNB2  PLPPR4  ATRNL1  RIMS3  CPNE7  DNM3  KIF3A  RAPGEFL1  SYNJ1  CHST1  MFSD6  CYP46A1  CAMKK2  KIRREL3  SPRYD3  PGBD5  DUSP26  ZNF365  GUCY1B3  TMOD2  PPM1H  CDKN2D  RASGRF2  CBX6  ERC2  C2CD4C  ARFGEF3  SEMA4A  GFOD1  PVRL1  NAP1L5  BCL2L2  GALNT9  ADCY1  STMN4  BASP1  PGM2L1  HDAC11  PRSS3  KIAA1211L  NEFM  MAP3K10  TCEAL6  NYAP1  ELAVL2  PDP1  LMTK3  DYNC1I1  BRSK1  GABRB2  NIPAL3  CALM3  RP5-1119A7.17  AP3B2  APLP1  ARPP21  KCNB1  IL34  SLC6A1  TTLL7  GAS7  KCNQ3  PPFIA2  MICAL2  HID1  CAP2  UBE2QL1  MOAP1  NKAIN2  SCN8A  NEFL  NCOA7  CDKL5  ARRB1  APBA1  CCK  RP11-434B12.1  PNMAL1  RTN4RL2  FAAH  BRSK2  SPOCK1  CAMK1G  MAPRE3  SEPT3  TMEM151A  SPRN  THRB  RP5-1187M17.10  TMCC2  GAD1  RP11-731J8.2  RALYL  ATP9A  STXBP6  MAST1  EXTL1  RCAN2  NDRG4  DIRAS2  ASPDH  GOLGA7B  SH3BGRL2  PCDHGC5  CYP26B1  BIN1  IQSEC1  TLN2  TMEM59L  GRIA2  GRAMD1B  ITPR1  PHACTR3  SPTBN4  KCNQ2  FBXW7  PPP3CA  CRYM  KLC2  NEFH  SSTR2  WNK2  TPRG1L  MYCBP2  OPCML  LY6H  TSPYL4  PHACTR1  SPINT2  UBE2C  RASD2  LAMP5  SV2A  LINGO1  GRASP  GABRA2  APBB1  LRP11  MADD  MIR7-3HG  TUBB4A  CLSTN2  DOC2B  PRDM8  PREPL  PTPRD  PARM1  GDAP1  MICU3  DGKZ  ELFN2  FAM171A1  YWHAG  LMTK2  ABLIM2  MYRIP  FCHO1  GNAI1  KLHL2  CAMK2G  PTK2B  IGFBP2  GPRASP2  ROGDI  TOLLIP  C10orf35  SEZ6L2  KCNQ5  NCAM2  SYNJ2  KCNAB1  SHTN1  ENO2  CELF3  NIPA1  AIFM3  SGIP1  FABP3  CNTNAP1  INA  PPP1R3F  PTGDS  ARHGEF9  PPFIA4  KCNC1  CACNA2D1  PALM  NALCN  NRXN1  LGI3  RBP4  GNAZ  KCNC3  AC096772.6  CDH22  HMP19  ACOT7  LRRC8B  SHC3  LARGE  KCNMA1  WASF1  RAPGEF5  CRY2  SNRPGP10  CABLES1  RAB26  CTIF  KBTBD11  DLG3  CAMTA2  TBC1D9  ATP6V0A1  ZNF204P  PPP3R1  PCSK2  MAP7  IGSF8  BEX2  RPLP0P6  LLNLF-187D8.1  ATP2B1  L1CAM  SLC9A6  TOP2A  PNMA6A  MAPK9  ATP6V1A  TUBB2A  FAM81A  SLC2A13  NRG3  NBEA  TCEAL5  PELI3  ANK2  TMSB4XP8  PLK2  LPCAT4  BTRC  NAT8L  TUB  RND1  AKAP11  MAPT  CALM1  H3F3AP4  MYBL2  KALRN  STXBP5  KIFC2  ERICH3  KCTD4  KCNS1  VAMP1  COL4A1  SLITRK1  AATK  DOCK9  MKL2  FNDC9  ADGRB3  OMG  CMIP  ANKRD34A  RP11-713C5.1  FXYD7  CLSTN1  PDYN  KCNIP2  PANX2  TYRO3  PCDH7  YPEL4  ISLR2  ELMOD1  WDR47  GLRB  C9orf172  SEMA4D  FTLP3  MPP2  TMEM179  ABR  RASGEF1A  SUN2  DCTN1  NUSAP1  NOS1AP  ACBD7  KLHL32  LDB2  GABBR2  MBNL2  RAPGEF2  MEF2D  GABRA3  B3GALT2  RPS18P12  EFR3B  GPRIN1  CACNB3  RAB27B  RASL10A  CADM3  ARHGAP32  LINC00634  ATP6V1C1  LRRC73  SSBP3  DIRAS1  PBK  HMGN2P5  ELAVL3  BAIAP3  SNORD113-3  RP3-406A7.7  RTN2  LARP6  AL450992.2  SPNS2  MAPK10  RP11-234A1.1  HPRT1  FAM49A  PTPRN2  BIRC5  CD200  MAP2K4  CGREF1  C1QL2  FAM131A  TCEAL2  AMIGO1  ANKRD24  PCP4L1  RP3-425C14.4  TOM1L2  HCN2  HK1  DIXDC1  AC016739.2  MRAP2  RPS3AP6  ANXA1  PIANP  NPTN  CDS1  PRKACB  CDH13  PRNP  QDPR  FAM189A1  WIF1  BEX1  ARHGAP23  ABCA2  RRM2  C1orf216  RPS3AP26  HIVEP2  RPL13AP5  SYT3  MMP17  MAP2K1  SBF1  EDIL3  C5orf30  INF2  DNAJC12  MDK  LINC00599  NRXN2  AGAP1  FAM64A  RP11-588K22.2  GJB6  THEM5  FOXM1  DGKB  RNU6-529P  PNMA2  IGIP  RTN3  MT-RNR2  FN3K  LANCL1  ANXA2  ASF1B  CMTM4  OPTN  RHOBTB2  RP11-36C20.1  OSBPL1A  INPP5A  TTC9B  SYNGAP1  ZNF25  ANXA2P2  RPL39P3  SEC14L5  NTNG2  TPT1P9  AMER2  SECISBP2L  HERC1  FAM234B  CTB-63M22.1  CAMSAP3  TMEM35  TRIM2  SCD  FBLL1  CADPS  CIT  SCN1B  SEC61A2  F2R  NACAD  ELOVL4  SORCS2  LGI4  AKAP6  RP11-761N21.2  TSPYL5  UCHL1  AGTPBP1  ARNT2  NPY  CNTNAP4  SH3PXD2A  PLCL1  ADGRB2  SIK3  MAP2  AAK1  PAIP2B  BTBD9  PIGCP1  BAIAP2  VIM  PDPN  ATP6V1H  KB-1460A1.5  SH3GLB2  KCNA2  ACSL6  ZER1  NAV3  EGR3  CHD3  B4GALT6  EEF1A1P5  ST6GALNAC5  PFKP  AC131263.1  FUT9  CKS2  MIAT  PXK  ATXN7L3  LINC00294  DNAJC5  ADGRL1  SLIT1  PRKAR2B  FGF12  DYNC1H1  CRTC1  EZH2  SGTB  RNF208  TENM2  RP11-425L10.1  ATP6V1B2  RPS2P5  COL4A2  B4GAT1  B3GAT1  LCNL1  FBXO44  NCEH1  SPOCK3  ACTL6B  TNC  CYTH1  CTC-251I16.1  GPR158  EFNA3  RPL24P8  OPRL1  GPR27  KIFC1  RPL13P12  C14orf132  STOX2  SLCO1A2  PLEKHG5  FBXO27  RPL18AP3  TMEM63C  RPS23P8  EMP3  CDK1  CDC42EP2  RPL37AP1  RPRML  ADAMTS8  SEPT4  NLK  TPRN  CACNB2  ADD2  EML2  MAP6D1  CENPU  STMN2  FAUP1  ANKRD65  BEGAIN  BEX4  PLP2  PRRT3  YBX1P1  HHATL  DPP10  RP11-1134I14.4  RANGAP1  ACTN2  C9orf91  TUBA1C  PAQR6  ZBTB7A  KCNIP4  LPGAT1  DHCR24  CSRNP3  EMX2OS  PRKAG2-AS1  FAM171B  KIF2C  CACNB4  TMSB4XP2  HECTD4  PLEKHG3  ITPK1  GPR162  FAM50B  LINC00320  OXCT1  C2CD2L  FAM124A  ATP6V0C  FGFR2  ELMO1  SYT12  PKP4  CLIP3  CPNE5  MINK1  EEF1A1P12  NECAP1  PLCB1  METTL7B  MYO18A  UNC80  TARSL2  CERCAM  EPHA4  RP11-778D9.4  CACNA1A  AURKB  EIF4EBP1  RGS14  DOHH  DACT3  LRRTM1  NES  OXR1  DNAJB2  TMSB4XP1  BCL11A  R3HDM1  YPEL2  RP11-285F7.2  SORBS2  TNFRSF12A  AGAP3  GRIN2C  RP11-92K2.2  TENM3  RPL5P34  SREBF2  EEF1A1P11  S100A10  PDIA2  MAN2A2  NDRG3  CELSR2  AC004453.8  TMEFF2  SMIM17  ID3  ADARB1  CCDC92  WDR37  PIP5K1C  CDIP1  SLC22A17  LDHD  SYNPO  AL162151.3  SNRPGP2  RPL12P4  CTD-2192J16.15  RPS20P14  CLIC1  RPL35P5  GRID1  CCNB2  RP11-127B20.2  ADRBK2  S100A11  OPALIN  CDCA7  KLHDC8A  RP5-1056L3.3  PRR36  PLEKHH1  LDOC1  FAM73A  NISCH  PIP4K2A  RP11-403A3.3  LRRC4  RPH3A  MYH14  RPL37P2  ELAVL4  EEF1A1P6  TTBK1  PPIAP31  KCND3  MDH1  RAPGEF3  FAM155A  RP11-318C24.1  BOK  HOMER1  RNF112  GS1-358P8.4  ENPP4  PDE4A  TPX2  REEP1  STEAP3  PPP1R9B  DISP2  WDR7  PRR18  SPARC  RIMS4  FAM171A2  FTLP2  GFRA2  ARHGEF4  RTP5  RP11-384O8.1  RP11-124N14.3  ZDHHC22  RP11-475C16.1  KIF5A  FASN  PDXP  FAM19A5  PLD3  SPTAN1  SCG5  CDCA8  TPT1P4  NDFIP2  TSPAN7  OIP5-AS1  SHISA9  MELK  UHRF1  LIPE  ACTR3B  SPINK8  TAGLN2  CNP  GRIPAP1  LMO3  CD99  KIF1B  AC079250.1  UBE2O  CENPM  HIP1R  DKK3  CMAS  UBR3  MYOM2  CRISPLD1  KCNIP3  CAMK2N2  FAM13C  RP11-112J1.1  SORCS3  CNTN1  RPS10P3  AC019129.2  C21orf62  RPL23AP42  TRNP1  CD44  RPS7P1  KIF4A  FOXO4  THY1  FAM65B  GALNT18  TMEM63A  NRIP2  RP6-65G23.3  NT5DC3  CDC20  ADRB1  RP11-690D19.3  DUSP7  EVI5L  LRRTM2  AGPAT4  HABP4  TULP4  COBL  MMD  ANGPT2  SCRT1  CTD-3035D6.1  OAT  DIP2C  RPLP0  HMGN2P3  ADGRA1  ACTBP2  RPS7P10  RPL7AP6  HSPH1  AC005330.2  PAFAH1B1  USP11  SC22CB-1E7.1  PTTG1  SLC48A1  RP11-69L16.5  HSPA2  NCALD  RPS6KA2  CDKN2C  GPRC5B  KIAA0930  FLRT1  SLC22A15  SNRPN  CNST  HPCAL1  C17orf96  PLEKHA4  MAP1LC3A  MIR600HG  SHISA7  TRIM23  SERINC1  TRAPPC6B  PRRG1  NTM-AS1  PPP1R37  KCNH8  TRIM37  ATCAY  GPR62  TSPAN15  SLC12A2  DTNB  LGI1  TBC1D24  C1orf233  SIRPA  GNB5  RHBDL1  GIT1  PFN1P1  SYBU  XXbac-BPG283O16.9  TIMP4  NR1D1  RP11-270C12.3  ATP13A2  LINC00672  CELF2  PINK1  NCAPH  RP1-278E11.3  ARHGEF37  PPP1R9A  FAM107B  CCSAP  LMNB1  SMIM3  DGCR5  KCTD8  KIAA1109  CRNDE  CX3CL1  PPM1K  CRTAC1  SERPINH1  FCGBP  KIAA0101  CCP110  KIF20A  CCDC80  SEMA3B  TMEM56  CCNA2  PTPRK  LINC00957  RP11-40C6.2  TTYH2  RASGRP1  ENTPD6  FBXL15  ASPA  MAP7D1  GJC2  DBNDD2  RPS2P46  POLR3A  CDC42BPB  FAM53B  SPC24  KIAA0232  DAGLA  FKBP10  FAM60A  NKIRAS1  TERF2IP  PPP1R14B  RPL34P18  IGFBP7  MAPRE2  TMEM240  MDFI  PIP4K2B  CSRP2  RPL14P1  RPS15AP1  RP11-57H14.4  RP11-760H22.2  GPR37  MAL  APOBEC3C  FBXL2  AP2A2  LHPP  CMTM3  NME2P1  RAB40C  AP3M2  CTD-2192J16.22  PLEKHB1  PCDH9  RPS11P5  CBLN4  CNTN2  BSCL2  LINC01105  HLA-DRA  GPIHBP1  MAPK8IP1  RAB11FIP2  RBFOX2  MAPK11  PDE1C  SEPT8  RP11-244O19.1  RPL15P3  CAPN3  UNC5C  PWWP2B  AVPI1  PPM1L  WEE1  MYO1D  CXCR4  SRGAP3  PRDM2  GHITM  NTM  SMIM13  SPOCK2  APOL4  FTH1P10  MEX3A  DYNLL2  RP11-12M9.3  FAM174B  TECPR2  NPAS1  FBXO34  CDC45  CLASP2  USP12  PRDX4  RPS13P2  ZFYVE27  HENMT1  HLA-DPA1  RAB42  DZIP3  CDK17  SPTBN1  TMEM235  SH3BP5  TMEM88B  MTCO2P2  EEF1A1P13  PRKCQ  PLLP  CTA-276O3.4  FOXD3-AS1  C20orf194  MT-ND1  ANXA11  RSPO2  TP53I11  MTCO1P40  RP11-212P7.1  BAIAP2-AS1  DCAF6  LHFPL4  STMN3  PPP1R1B  BANF1P3  SNX32  KIF18B  DYSF  RASGEF1C  SYNDIG1  PPM1E  ANXA5  NBL1  CTXN1  RPL9P7  MYRF  ANLN  S1PR5  CADM2  TTC7B  CECR6  SLC45A1  MPRIP  KIAA1161  ZFYVE9  RPS7P11  S100A6  HR  RP11-74E22.8  PPP1R13B  FCER1G  EPT1  RP11-51O6.1  CDK18  ETS2  KIF21B  NDC80  CHIC1  FSTL1  CPEB1  HS3ST2  SPC25  CCNB1  RPL41P2  CCDC6  KIAA1324L  GRIA1  IGSF21  DDX24  CCSER2  NSMF  PLPP2  RP11-490M8.1  TNFRSF19  ZDHHC8P1  NCAM1  PSMB8  PITHD1  RP11-627K11.1  HJURP  LINC00936  PPP1R14C  CTB-79E8.3  SLC25A23  SLC39A10  WDR17  ABHD8  ENPP2  DMXL2  MT2P1  TROAP  ERMN  CAMTA1  AC018647.3  GPX8  NEBL  CPXM1  USP54  PLXNA2  RPL10AP6  RUFY3  ADAM23  LPL  RP11-632C17__A.1  CPVL  TMEM38A  NEDD4L  CDYL2  AEBP1  MLLT11  FA2H  C15orf59  CLN8  KIAA1462  RPL26P19  RGS11  HAPLN2  KIF17  RP11-809C18.3  DST  CCDC184  RP3-340B19.2  JPH1  CAMSAP1  NRM  KLF13  CACNA1G  GNG5  PITPNM1  ADAM22  DDHD2  B2M  H3F3AP6  PYGL  RIPPLY2  TSPAN6  NMB  NPM1P27  EIF4A1P10  PITPNM2  SSX2IP  RABGAP1L  EFHD1  MAP1B  RP11-466H18.1  YJEFN3  HMGCS1  ARHGAP26  MGEA5  FBXL19  RNF220  NETO1  CLEC5A  G3BP2  NFIA-AS2  EFR3A  DYNLT1  RPS3A  MEOX2  SGSM2  SPRYD7  CD99L2  HLA-A  RP5-907C10.3  PARK2  RORB  SVIP  GARNL3  MAPK3  CTNNA2  RP11-464D20.2  PDXK  HOTAIRM1  RAB11FIP5  LINC00205  NAPG  RPL39  HLA-B  IPO13  RAB13  TMTC4  ERBB4  MAG  GUCY1A3  MMP14  ACVR1B  NT5DC2  TF  SERP2  PPL  FAM219A  LMBRD2  CDHR1  AC007969.5  CHI3L1  MEST  TMEM144  CD74  RPL24P4  MTA3  PROS1  ACADSB  C10orf90  SMARCA2  RPS6KL1  MAPK1  C12orf76  MBP  MRC2  SLC45A3  EPHX4  BUB1  FOXD1  H3F3A  KLC1  MOBP  TUBAP2  HLA-DPB1  PPP1R12B  CENPA  PLP1  NCAPG  ATP6V1E1  MAFK  TUBB6  RPS3AP5  EEF1B2P3  APOC1  PPIC  LINC00404  PLPPR3  TGFB1I1  NR4A2  STMN1  FOLH1  PSMB9  FLNA  FAM131B  INPP4A  WASF3  SLC2A6  SOCS7  RP11-543P15.1  NKX6-2  TMSB15A  FAM174A  ASPHD2  MTHFD2  NOVA2  CHRD  RPS19P1  VCAM1  FAM126B  IDI1  RPL13AP7  DSTYK  RUNDC3B  NECAB3  GTSE1  GNG2  PDIA4  GBP2  HOXA5  TMEM125  DLGAP5  CDK2  SORT1  RPS2  RPS2P7  FTH1P2  CNIH2  GPX7  HLA-DMA  RTN4  LGALS1  SRP9P1  DRP2  DTL  CFI  ATP1A2  FAXC  RPL4P4  CDCA7L  PIP5K1B  COL4A5  PAQR8  HAS2  MEGF9  RPL23AP65  TSPAN12  CHN2  KIAA0040  CLMN  CD276  SLCO3A1  WWTR1  KLHL26  CARD16  PTMAP2  RNF165  CARNS1  STEAP2  RP11-641D5.1  HERC2  CNDP1  SEMA4F  SLC9A7  LINC00643  LIN7B  LRRTM4  SRPX2  KCNJ10  CA4  RELL2  PC  MYH10  C6orf136  DCLK1  NXPE3  LIMA1  PRRC2B  YBX1  SMAD7  RUSC1  SKA3  CD58  MROH1  LFNG  TMSB10P1  HRK  TAGLN2P1  PCNA  PLVAP  MCM2  BTN3A2  IQGAP2  FXYD1  NHLRC1  SMPD3  CAPG  SNX30  PHYHIPL  MYPOP  PPP2R2B  APBA2  SYNE1  RANP1  SLC40A1  DIO2  FTH1P11  PADI2  ENTPD4  AP2A1  DGKQ  KLK6  SLC16A4  CENPF  RIMKLA  S100A16  CCDC3  TP53  CTD-2206G10.2  NINJ2  RPL13AP25  ULK1  CTD-2031P19.4  NR1D2  MT-TF  OPA1  PPM1A  MOG  GAPDHP65  RP11-1055B8.3  FTH1P8  FRS3  LINC00403  NRN1  ARX  TIAM1  SATB1  CD93  C16orf45  BHLHB9  MKI67  MSR1  AC016700.6  PGP  COG1  USP46  AURKA  GRIA3  RAB32  CLCN6  ABCC3  HSPB1P1  RP11-603J24.7  ZBTB18  NUF2  PPP2R5B  RBP7  DEPTOR  RP11-1134I14.8  TMC7  RPS6KA4  ARHGAP35  BUB1B  MT-ND6  MTMR9  TFPI  IDH1  EZH1  MVB12B  RP11-698N11.2  TTC3  TMEM45A  NTN1  EPS15  PCDH1  CMTM5  HMGN1P38  CD248  EEF1A1P19  EFEMP1  AACS  RP11-403A3.2  RP11-380L11.4  HLA-DMB  C8orf46  RP11-592N21.1  CNN3  ZYG11B  RP11-834C11.4  RAD51AP1  KIAA0368  OIP5  CTB-33G10.1  ACO2  PICK1  NOL4  RBP1  KIF21A  FZD7  ABHD17B  SPOCD1  MRVI1  ARF3  SYDE1  WI2-1896O14.1  ALOX5AP  PTPRZ1  TREM2  LINC00657  ARHGEF17  ST14  MMP2  RPL18A  ELN  EEF1A1  JAG2  EEF1A1P9  MEIS3  SLC5A11  FAM217B  GLDN  PLA2G16  PTPN4  LIMCH1  EGFL7  CD151  SLC38A1  EXOC6B  HMGCR  GAPDHP63  MEF2A  HTT  HOXC10  SUMO2P1  PHLDB1  TMEM170B  MTND2P28  AC092933.3  LZTS2  HLA-DRB1  USP27X  EIF4E3  RP11-478C6.4  ITFG1  S100A4  GNG12  KIF15  RP11-364B14.3  EFHD2  PRKCD  AC002456.2  SLK  ZNF540  NDUFAF4  FAM102A  HLA-DQA2  HMGB2  TCF19  GBP1  GPR176  LDLRAD4  C21orf91  TMTC1  FCGRT  RB1CC1  MORN4  TMEM150C  STS  ABCC8  COL9A2  CLDND1  VPS53  CLDN10  GPR155  GALNT16  PRRX1  DPYSL2  TYROBP  CLDN11  DUSP3  NCKIPSD  GPX1P1  S1PR3  MMP9  NT5DC1  TEX2  SHOX2  FABP5P7  MSN  ELK1  PNMA1  GDI1  LAPTM5  CCDC136  PTPRM  ZDHHC11B  TRIM3  BMPR2  RP11-452N17.1  FTH1P4  LPAR1  LINC01158  TTBK2  C1R  TMSB4XP4  KIAA1217  REEP5  RPL7AP30  MRAS  NXT1  RP11-84A19.3  NPC2  PLXNB3  CALB2  KIFAP3  KBTBD6  VAMP5  EGFR  SPRY1  CDKN1C  PCDH19  MEOX2-AS1  CA9  WNT7B  ALDH5A1  SLC39A12  ACSL4  RPSAP12  GAL3ST4  ACTL6A  RP3-417G15.1  ARFGEF2  MS4A6A  ABCA3  DBI  RNASEH2A  PRICKLE2  ADAMTS4  MT-ND5  ITGA5  BRWD1  JADE2  MTRNR2L2  PITRM1  RHOC  PDE6B  JAG1  FAXDC2  RP11-20O24.4  TTC9  NDEL1  PHLPP2  KIF1BP  RP11-331N16.1  SAMD8  DENND6B  FAM168B  ATP5EP2  YPEL3  TBX15  RUFY2  SOBP  CPEB2  RPL3P4  CLTB  MYL12A  RPSA  MAP1S  RBBP8  RCC1  LRRN4CL  PDK2  RP11-343H5.4  TPI1P1  AC010468.1  MBOAT7  SLC25A18  CCDC85B  GINS2  EMP1  STAM  RP11-126K1.6  BZW1P2  ZIC1  MAFG  NEURL4  CEBPD  SLC25A44  ABCA8  DNM1L  TESC  DTYMK  SYTL2  VEGFA  ETNPPL  ICA1  PDCD1LG2  TCF20  APC  SLC43A3  ARHGDIB  RPS3AP47  SOX4  FAM69A  MCF2L  MTUS1  ULK2  SLC2A3  HDAC5  RNASE2  SPIN1  C19orf81  RNASE6  ATE1  PROCR  CDKN3  RNF6  CDC42BPA  CADPS2  ENHO  SPTLC2  TK1  STAU2  FAM57B  FCGR3A  C19orf68  RAP1GDS1  ZMYND11  ARPP19  PACS2  CDC42P6  ANTXR2  PPP1R12C  CD63  MT-RNR1  FAM134B  TMEM37  POPDC3  HINT3  BAG4  BRI3BP  SLC25A4  CHADL  PCDH8  CYLD  KCTD17  TMEM132C  SPATA2  RPS2P55  SHCBP1  ACTR1A  CTD-2270N23.1  RDH10  GBP3  TRAM1L1  USP32  SUSD4  IL32  BX322557.10  EEF1A1P4  FAM13B  SNRPG  FAM169A  IDH3A  MIR4482  RP11-192H23.8  DPF1  SREBF1  RPS10  GJB1  BTBD3  RPP25  LINC00511  VPS13D  PPP1R3E  S100A3  TNK2  ETV1  RP11-3P17.5  CIPC  IQCJ-SCHIP1  TAC3  LIG4  RPS15A  DMRTA2  OSTC  NFASC  ST6GALNAC6  C1orf226  TMEM25  ANKH  RPL35P1  CTD-2510F5.4  SLC25A12  ARHGAP11A  COX6A1P2  AC064875.2  PIK3CB  CALU  FAM161B  PTGES3P1  TRIM44  PRKAG2  KDELC2  PCNT  EID2B  DEAF1  MT-ND2  FTH1P12  RP11-524D16__A.3  SCG3  VAMP8  DBP  GOT2  RYBP  WAC-AS1  CD55  CALD1  CNRIP1  MT1XP1  GAL3ST1  IFI35  NTSR2  CNGA3  STOX1  THRA  SLC45A4  CEP55  WBP2  MTMR12  UCP2  RPS19  RNF11  TPD52  GNG7  PCMT1  CYBA  EN1  GBF1  NOL4L  OSMR  PDLIM3  RP11-88H12.2  C2orf69  FKBP8  RPL7P1  LINC01579  HSPA6  TEAD2  DFNB31  MAP3K7CL  RP5-1052M9.1  LYPD1  GNAQ  DVL1  TRIP6  PDLIM1  SIN3B  RNU7-123P  PLSCR1  RPE65  ADRBK1  PEBP1  LYPLA1  BRINP2  KCTD1  HOXB7  6-Mar  BLCAP  TMEM132B  NEK2  ATP6V0E1  TSR2  STK17A  TBC1D12  TRUB1  PER3  CCNY  PLCL2  ZFYVE28  CLIP4  PTK7  UROS  RP11-587D21.1  SHOC2  HNRNPKP4  YBX1P10  IFI16  NKRF  RPL7P9  RAB20  FRY  TACC1  UGT8  PLA2G4C  HMMR  ATRAID  SCN1A  ABTB1  FAM111B  GPR65  KIAA1755  TRPM2  HNRNPUP1  ROMO1  LAMC1  UHRF1BP1L  LAMA4  TRIB3  PJA2  FHOD3  PYGM  STON1  HERC3  MIR4442  TNFSF13B  SRRM3  DNAJC27  EVA1B  RP11-372E1.1  SERPING1  NKX2-5  FIBIN  SERINC2  KLF16  AC005251.3  CDS2  ERMP1  RPL5P4  CTD-2195M15.1  NCF2  RP11-864N7.2  TMC6  ARHGAP21  MBLAC2  YBX1P2  DPEP1  LRRC10B  CDH10  CASP1  HSD11B1L  BCAN  FXR2  HOXD10  CBX2  ZYX  MAP6  HYAL2  TGIF1  PCSK1  LARP1  KCNJ11  RANBP3L  RPL31P49  PPP1R14A  YWHAB  SLC13A3  PPIP5K1  MTCO1P53  PLA2G6  CPEB4  NDRG2  SKA1  RP3-423B22.5  FAM20B  FNBP1  TUBG2  CSTA  RPS7  AP000936.1  CISD1  DOPEY1  TMEM74B  EFEMP2  ERBB3  AC004540.4  SLC27A4  RP11-365O16.1  C17orf51  SMAP2  SLC25A42  RPL6P27  TTK  GAPDHP60  CD300A  RP11-436H11.1  ANG  ATP6V0D1  AFAP1L1  TMEM176B  MIR25  RP3-402G11.25  RP11-1017G21.5  PTN  CACNA1H  FTH1P5  DLG1  RPS15AP24  HLA-DRB6  S100A2  ZRANB1  DSCAML1  SLC2A10  SH2D3C  TNRC6C  SRPK2  HMGN2P4  FCGR2A  MICAL3  HSPB3  BMF  COX5BP6  ALCAM  ODC1  MT-CO3  KIF23  SGPP2  BCHE  DNAL4  MGLL  LSM11  TRIM46  RPL37P23  RGS1  NUDT10  RP11-488C13.1  ANKRD11  AARS  ACY3  C16orf70  SOX2  SOCS1  NCF4  RALGPS1  DNALI1  C1QC  CKS1B  NR3C2  GNB2L1  RNF122  EVI2A  C4orf47  RP11-249L21.4  EPHB4  HOXD9  RP11-347E10.1  CENPK  RNF175  CA10  PARP9  CKAP2L  TRIM22  COPZ2  HNRNPA1P48  CXCL16  LAMB3  ITPRIPL1  SLC11A1  RAD51  RP11-617F23.1  WFS1  RP11-288E14.2  ASRGL1  TRIM21  TYMS  KIF3B  NNMT  RAB3GAP1  C1S  KIF11  MYD88  MID1IP1  ID4  C1QB  USP31  CHL1  PKIA  ANO5  RP2  MGAT5  A2M  RILPL1  SLC7A7  RP11-680H20.1  ATP6V1D  RAI1  LRSAM1  GINS1  PPP1R26  ADAMTS15  GAPDHP40  ALDH2  PHLDA1  EIF4A2  KLHDC3  MYC  HOXC4  C22orf29  RP11-384F7.2  CHST14  RND2  ARSJ  SCAI  FPR3  HIPK2  TCEAL7  LOXL3  CA12  LRFN1  C16orf52  IGF2BP2  NAMPT  HAGH  SERINC3  SYT11  CCNA1  CENPH  ASH1L  PLK1  GAPDHP72  RMND5A  FANCI  RP11-832N8.1  PLEKHG2  TMEM184B  HAUS1  ETV4  RGS20  LAMB2  RPL35P2  ALDH6A1  GCC2  RPL29  FRG1BP  R3HDM2  HHIPL1  ZDBF2  AZIN2  AB019441.29  DHRSX  ZWINT  PIN1  TBRG1  FLYWCH1  REXO1  PPP2R2D  SLC4A3  NDN  PRKAR1A  HOXB2  PTBP1  SLAMF8  RAB9B  CCDC167  PKI55  DENND5B  RP11-401L13.4  KIAA1147  RPSAP54  UBQLN2  OLFML3  MARVELD1  STOML1  KLHL11  CSRP1  ABHD6  TGIF2  GDAP1L1  MNT  BBS7  ENDOD1  TCP11L1  IBSP  MTCO3P12  CAMSAP2  AC092171.4  VAX2  C6orf106  PDE8B  BMP1  RPS15AP11  MFSD13A  MASP1  GNL1  RAB6A  AC147651.4  HSPB1  DDX25  LRRC4C  ME1  RPL7AP66  BACE1  SOX11  TMEM106C  CASP4  CPOX  RP1-159A19.3  FXYD5  RNF157  DPYD  REEP2  GMNN  C1orf162  S100A1  IL13RA2  HECTD1  YBX3  GPD1L  TP53I3  NIPAL2  ADGRB1  CKLF  KIAA1468  FTH1P16  CD48  IFITM10  RPSAP15  SLC1A5  RNF135  ANKRD46  RP11-170M17.2  WDR76  SLC16A3  USP20  DPYSL3  TTLL11  SHMT2  SPRY4  AMN1  PABPC1L  GSDMD  DPP6  RPL4P5  RPS5  HSPE1P2  AMPD2  HLA-DQA1  HLA-C  PIGZ  SEMA3G  TNNT1  MLXIPL  PSME2P2  CLCF1  AC007387.2  TMEM8B  EVL  GMFG  ACKR3  ECM2  PPP1R1A  GUSB  UBBP1  GIMAP2  RP11-676M6.1  SLC4A8  KCTD2  TMEM255A  BDH1  SKP1P1  SETP14  RP11-889L3.1  SSR2  RP5-827C21.1  SHF  C1RL  AUH  CDK19  FSD1  PKD1  SCAMP1  PLEKHB2  FAM102B  ZBTB4  PLOD2  GAS2L3  MTCYBP18  PRR11  PSMB8-AS1  MTND6P4  NME4  ZDHHC17  HLA-DOA  ITGB1P1  RNU6-415P  HCG4P5  GBE1  CAV1  PLTP  C1QA  RPS14  NAPSB  MTCH1  ORC1  PPP1R7  LUZP1  AKT3  PARVB  RP3-375P9.2  RP11-351I24.3  YWHAZ  SIGLEC7  NICN1  PCNXL2  RP11-257O5.2  POU6F1  TIMELESS  ZSCAN18  ARHGEF11  BTG1  PRKCQ-AS1  PIPOX  HSD11B1  SLC23A2  TMEM178B  PRCP  CSGALNACT1  TYMP  RPS18  RPS15AP38  FKBP1B  STAC2  C1orf54  EDNRA  SYT17  DTX3L  ATAD1  RP11-299L17.3  TRAF4  UHMK1  PRKCA  SUCLA2  TIMM8B  FMNL1  GAS1  ASTN1  SCAF1  IKBIP  STRN  ARPC1B  PDPK1  RP1-232L24.3  RP11-467L13.5  STRIP2  NUDT14  RP11-244J10.1  RP11-214N15.5  EIF1P3  CD53  BCAS1  PPP1R3B  COL26A1  WSB2  TMEM178A  RABEP1  ADAMTSL2  MCM5  BRAF  RP11-566E18.1  CTD-3035K23.7  ABCA1  MAPK8  RHOU  NXT2  BCL2L12  ANTXR1  UPF2  ADAM9  RP4-604A21.1  PPP1R18  PTGS1  FAM83D  SIK2  FKBP9  PRC1  FBXO31  IFI44  RPL13AP20  GPX1  RP11-613F7.1  SBNO1  LSM2  FANCD2  C7orf73  DIP2B  FAM111A  MEGF8  STAC  MUC1  RPS3  LRRC20  AC004057.1  COL4A3BP  AC107983.4  AC006538.1  CHEK1  WDFY3  SAT1  PLAU  MPHOSPH8  CCDC109B  FAM222A  PLAT  LRRTM3  LGALS3  ATP2A2  ATXN7L3B  TNFAIP6  SPSB4  SLC1A4  SLC25A48  NLGN2  SERPINA1  NUDT11  LDLRAD3  PTK2  ITGB2  RP4-706A16.3  RPL5P1  ANO6  TJP2  PRSS23  TBX2-AS1  RAC2  DOCK10  LYRM9  IL1RAP  TSC1  USP35  RAB34  RP11-887P2.3  MAD2L1  CAND2  RPL21  TSPO  FSD1L  TXNIP  SGSM3  HS3ST3B1  EIF4G3  NECAP2  MT-CO1  RPL10  RPL29P11  CDCA4  CTSC  KIAA0430  HMOX2  CKB  FABP7  VCAN  IRX1  ZC3H13  KDELR2  MT-TP  RP11-321C24.4  PTMAP4  ADCY2  MAD2L2  ZNF248  SYTL4  SOX2-OT  RP11-434H6.7  TESK2  PQLC1  GPI  SLC9B2  TMED8  TSPAN5  YWHAEP5  TAOK2  FBP1  SQRDL  TNFRSF1A  SAMD9L  RRAS  RP11-796G6.1  RPL3P2  RPS16  SFXN3  LRRC4B  LEFTY2  TRIM47  TMSB4X  ABHD4  RP11-78O7.2  ATG2B  TYMSOS  ARHGEF7  AFF3  PALLD  RP11-490G8.1  EDA2R  RGS12  ABLIM1  LYPD5  ZNF667  FBXW4  SLC6A12  ASCL1  FAM149A  CASP6  KCNMB4  LDLRAP1  DEPDC1B  OST4  SLC1A1  WDR34  ECSCR  PLOD1  RPS11  RP3-460G2.2  RBM8B  WNT5A  RP5-1125N11.2  SUGP2  NACC2  F2RL1  PPP4C  NUPR1  MT1G  FAM184A  RPS29  MT2A  RP11-382A20.3  HOXB3  TCF12 | 3.80E-47  1.14E-35  1.27E-32  9.31E-31  4.37E-30  1.88E-29  2.09E-29  1.05E-28  2.97E-27  6.03E-27  1.32E-26  6.77E-26  1.18E-25  1.83E-25  4.53E-25  8.21E-25  2.02E-24  2.09E-24  2.23E-24  2.48E-24  3.19E-24  6.31E-24  7.54E-24  9.57E-24  1.06E-23  1.38E-23  1.99E-23  2.13E-23  2.20E-23  2.45E-23  4.20E-23  4.49E-23  4.87E-23  6.31E-23  6.56E-23  1.36E-22  1.64E-22  1.86E-22  2.59E-22  3.01E-22  3.06E-22  3.25E-22  3.94E-22  4.39E-22  5.48E-22  5.95E-22  8.42E-22  8.43E-22  1.13E-21  1.61E-21  2.87E-21  3.72E-21  3.83E-21  5.56E-21  5.77E-21  5.88E-21  7.59E-21  7.83E-21  8.14E-21  8.55E-21  8.97E-21  9.74E-21  9.79E-21  9.90E-21  1.26E-20  1.33E-20  1.47E-20  1.58E-20  1.86E-20  2.17E-20  2.35E-20  3.20E-20  3.78E-20  3.90E-20  3.91E-20  4.52E-20  5.37E-20  6.57E-20  1.06E-19  1.16E-19  1.21E-19  1.22E-19  1.29E-19  1.30E-19  1.34E-19  1.50E-19  1.67E-19  1.70E-19  1.99E-19  2.35E-19  2.58E-19  2.92E-19  3.32E-19  3.35E-19  4.58E-19  4.67E-19  5.03E-19  5.79E-19  6.55E-19  7.77E-19  7.81E-19  8.20E-19  8.70E-19  9.12E-19  1.11E-18  1.14E-18  1.25E-18  1.30E-18  1.33E-18  1.50E-18  1.59E-18  1.72E-18  1.82E-18  1.84E-18  1.94E-18  2.03E-18  2.07E-18  2.09E-18  2.63E-18  2.67E-18  2.97E-18  3.03E-18  3.71E-18  3.88E-18  3.89E-18  3.96E-18  4.08E-18  4.18E-18  4.30E-18  4.50E-18  4.50E-18  4.70E-18  5.00E-18  5.34E-18  5.46E-18  5.55E-18  6.00E-18  6.04E-18  6.13E-18  6.79E-18  7.01E-18  7.73E-18  8.51E-18  8.60E-18  9.97E-18  9.99E-18  1.03E-17  1.07E-17  1.20E-17  1.32E-17  1.35E-17  1.56E-17  1.58E-17  1.71E-17  1.75E-17  1.84E-17  1.95E-17  1.98E-17  2.01E-17  2.11E-17  2.24E-17  2.25E-17  2.30E-17  2.33E-17  2.52E-17  2.83E-17  2.89E-17  3.72E-17  3.73E-17  3.79E-17  4.05E-17  4.54E-17  5.16E-17  5.28E-17  5.62E-17  5.86E-17  5.96E-17  6.68E-17  7.82E-17  1.14E-16  1.16E-16  1.31E-16  1.34E-16  1.39E-16  1.52E-16  1.55E-16  1.61E-16  1.72E-16  1.78E-16  1.92E-16  1.95E-16  2.11E-16  2.40E-16  2.58E-16  2.59E-16  2.67E-16  2.83E-16  3.20E-16  3.21E-16  3.23E-16  3.52E-16  3.65E-16  4.06E-16  4.70E-16  4.81E-16  5.09E-16  5.23E-16  5.26E-16  5.40E-16  5.68E-16  5.80E-16  6.42E-16  6.71E-16  6.92E-16  7.31E-16  7.32E-16  8.30E-16  9.15E-16  9.15E-16  9.18E-16  9.26E-16  9.60E-16  9.75E-16  1.22E-15  1.22E-15  1.44E-15  1.47E-15  1.47E-15  1.50E-15  1.57E-15  1.60E-15  1.69E-15  1.75E-15  1.87E-15  1.89E-15  2.44E-15  2.50E-15  2.92E-15  3.27E-15  3.27E-15  3.54E-15  3.84E-15  4.11E-15  4.59E-15  4.88E-15  5.06E-15  5.61E-15  5.64E-15  5.90E-15  6.15E-15  6.21E-15  6.29E-15  6.52E-15  7.00E-15  7.08E-15  7.60E-15  7.96E-15  8.42E-15  8.61E-15  9.49E-15  9.60E-15  1.05E-14  1.07E-14  1.13E-14  1.15E-14  1.16E-14  1.27E-14  1.35E-14  1.36E-14  1.44E-14  1.46E-14  1.54E-14  1.67E-14  1.86E-14  1.93E-14  1.95E-14  2.04E-14  2.14E-14  2.15E-14  2.21E-14  2.41E-14  2.41E-14  2.49E-14  2.71E-14  2.96E-14  3.45E-14  3.59E-14  3.80E-14  4.00E-14  4.04E-14  4.06E-14  4.24E-14  4.25E-14  4.32E-14  4.53E-14  4.61E-14  4.68E-14  4.88E-14  4.93E-14  5.53E-14  6.49E-14  6.60E-14  6.69E-14  6.98E-14  7.04E-14  7.71E-14  8.04E-14  8.55E-14  9.29E-14  9.41E-14  1.08E-13  1.10E-13  1.26E-13  1.27E-13  1.32E-13  1.32E-13  1.33E-13  1.60E-13  1.71E-13  1.78E-13  1.87E-13  1.93E-13  2.22E-13  2.25E-13  2.61E-13  2.64E-13  2.69E-13  2.81E-13  2.92E-13  2.95E-13  3.02E-13  3.04E-13  3.08E-13  3.22E-13  3.23E-13  3.48E-13  3.77E-13  4.22E-13  4.34E-13  4.46E-13  4.94E-13  5.54E-13  6.81E-13  7.20E-13  7.37E-13  7.40E-13  7.44E-13  8.25E-13  8.82E-13  9.48E-13  9.54E-13  1.22E-12  1.27E-12  1.31E-12  1.33E-12  1.40E-12  1.47E-12  1.49E-12  1.52E-12  1.54E-12  1.56E-12  1.61E-12  1.64E-12  1.82E-12  1.90E-12  1.91E-12  1.93E-12  2.09E-12  2.34E-12  2.67E-12  2.71E-12  2.81E-12  2.87E-12  2.93E-12  3.12E-12  3.28E-12  3.58E-12  3.79E-12  4.63E-12  4.83E-12  5.01E-12  5.72E-12  6.18E-12  6.45E-12  6.51E-12  7.23E-12  7.64E-12  7.70E-12  8.67E-12  8.75E-12  9.35E-12  9.94E-12  1.02E-11  1.03E-11  1.04E-11  1.06E-11  1.06E-11  1.18E-11  1.25E-11  1.35E-11  1.37E-11  1.60E-11  1.65E-11  1.71E-11  1.82E-11  1.91E-11  1.98E-11  2.09E-11  2.09E-11  2.38E-11  2.54E-11  2.71E-11  2.74E-11  3.07E-11  3.29E-11  3.98E-11  4.14E-11  4.15E-11  4.21E-11  4.22E-11  4.97E-11  5.22E-11  5.34E-11  5.69E-11  5.71E-11  6.14E-11  6.81E-11  7.18E-11  7.23E-11  7.34E-11  7.36E-11  7.41E-11  7.54E-11  8.00E-11  8.03E-11  8.12E-11  8.45E-11  9.35E-11  9.60E-11  9.60E-11  9.71E-11  9.97E-11  1.00E-10  1.22E-10  1.24E-10  1.31E-10  1.34E-10  1.37E-10  1.49E-10  1.50E-10  1.50E-10  1.60E-10  1.75E-10  1.77E-10  1.81E-10  2.00E-10  2.02E-10  2.12E-10  2.16E-10  2.23E-10  2.42E-10  2.63E-10  2.85E-10  2.85E-10  2.89E-10  2.90E-10  3.02E-10  3.05E-10  3.17E-10  3.22E-10  3.66E-10  3.67E-10  3.93E-10  4.01E-10  4.09E-10  4.39E-10  4.79E-10  4.90E-10  5.57E-10  5.63E-10  5.64E-10  5.67E-10  5.73E-10  5.94E-10  6.06E-10  6.07E-10  6.25E-10  6.38E-10  7.28E-10  8.02E-10  8.19E-10  8.46E-10  9.16E-10  9.38E-10  9.49E-10  9.49E-10  1.03E-09  1.08E-09  1.09E-09  1.10E-09  1.14E-09  1.14E-09  1.21E-09  1.22E-09  1.23E-09  1.23E-09  1.31E-09  1.48E-09  1.49E-09  1.49E-09  1.58E-09  1.61E-09  1.62E-09  1.69E-09  1.69E-09  1.72E-09  1.76E-09  1.91E-09  2.05E-09  2.07E-09  2.12E-09  2.15E-09  2.18E-09  2.29E-09  2.37E-09  2.45E-09  2.48E-09  2.74E-09  2.89E-09  3.01E-09  3.18E-09  3.34E-09  3.35E-09  3.70E-09  3.92E-09  4.27E-09  4.45E-09  4.61E-09  4.86E-09  4.87E-09  4.95E-09  5.12E-09  5.20E-09  5.22E-09  5.34E-09  5.54E-09  5.56E-09  6.32E-09  6.57E-09  6.64E-09  6.93E-09  7.55E-09  7.57E-09  7.79E-09  7.80E-09  7.89E-09  8.20E-09  8.44E-09  8.56E-09  8.56E-09  8.99E-09  9.04E-09  9.45E-09  1.00E-08  1.08E-08  1.09E-08  1.15E-08  1.15E-08  1.17E-08  1.25E-08  1.26E-08  1.27E-08  1.27E-08  1.34E-08  1.34E-08  1.48E-08  1.49E-08  1.53E-08  1.53E-08  1.56E-08  1.66E-08  1.68E-08  1.88E-08  1.89E-08  2.05E-08  2.05E-08  2.22E-08  2.29E-08  2.30E-08  2.32E-08  2.32E-08  2.35E-08  2.36E-08  2.44E-08  2.44E-08  2.66E-08  2.72E-08  2.76E-08  2.83E-08  2.97E-08  2.98E-08  3.03E-08  3.15E-08  3.27E-08  3.28E-08  3.31E-08  3.47E-08  3.56E-08  3.60E-08  3.63E-08  3.65E-08  3.97E-08  4.09E-08  4.41E-08  4.63E-08  4.68E-08  4.73E-08  4.81E-08  4.87E-08  4.88E-08  4.89E-08  4.90E-08  4.90E-08  5.13E-08  5.55E-08  5.66E-08  5.70E-08  5.81E-08  5.83E-08  5.96E-08  6.35E-08  6.37E-08  6.51E-08  6.58E-08  6.72E-08  6.99E-08  7.25E-08  7.25E-08  7.26E-08  7.63E-08  7.92E-08  7.99E-08  8.18E-08  8.58E-08  8.69E-08  8.72E-08  9.02E-08  9.39E-08  9.77E-08  9.79E-08  9.97E-08  1.05E-07  1.06E-07  1.06E-07  1.10E-07  1.16E-07  1.17E-07  1.18E-07  1.19E-07  1.22E-07  1.23E-07  1.31E-07  1.32E-07  1.34E-07  1.34E-07  1.36E-07  1.42E-07  1.48E-07  1.53E-07  1.53E-07  1.54E-07  1.54E-07  1.56E-07  1.61E-07  1.63E-07  1.64E-07  1.64E-07  1.69E-07  1.74E-07  1.76E-07  1.79E-07  1.81E-07  1.89E-07  1.89E-07  1.89E-07  1.97E-07  2.00E-07  2.13E-07  2.17E-07  2.18E-07  2.19E-07  2.20E-07  2.21E-07  2.23E-07  2.28E-07  2.29E-07  2.29E-07  2.29E-07  2.32E-07  2.43E-07  2.47E-07  2.50E-07  2.54E-07  2.54E-07  2.57E-07  2.63E-07  2.73E-07  2.85E-07  2.87E-07  2.96E-07  2.97E-07  3.09E-07  3.11E-07  3.14E-07  3.31E-07  3.40E-07  3.60E-07  3.60E-07  3.66E-07  3.69E-07  3.72E-07  3.79E-07  3.90E-07  3.92E-07  4.00E-07  4.00E-07  4.00E-07  4.07E-07  4.16E-07  4.17E-07  4.19E-07  4.34E-07  4.34E-07  4.41E-07  4.59E-07  4.60E-07  4.77E-07  4.87E-07  5.03E-07  5.15E-07  5.21E-07  5.34E-07  5.51E-07  5.57E-07  5.57E-07  5.58E-07  5.65E-07  5.82E-07  5.84E-07  5.95E-07  5.99E-07  6.10E-07  6.16E-07  6.18E-07  6.20E-07  6.31E-07  6.33E-07  6.34E-07  6.40E-07  6.57E-07  6.82E-07  6.82E-07  6.98E-07  7.03E-07  7.22E-07  7.26E-07  7.28E-07  7.29E-07  7.37E-07  7.44E-07  7.67E-07  7.70E-07  7.70E-07  7.97E-07  7.98E-07  8.12E-07  8.15E-07  8.28E-07  8.28E-07  8.40E-07  8.49E-07  8.53E-07  8.73E-07  8.76E-07  9.14E-07  9.35E-07  9.39E-07  9.42E-07  9.54E-07  9.62E-07  9.78E-07  9.97E-07  9.98E-07  1.02E-06  1.02E-06  1.02E-06  1.05E-06  1.06E-06  1.13E-06  1.15E-06  1.18E-06  1.20E-06  1.21E-06  1.22E-06  1.23E-06  1.25E-06  1.25E-06  1.25E-06  1.26E-06  1.28E-06  1.29E-06  1.29E-06  1.33E-06  1.34E-06  1.35E-06  1.38E-06  1.43E-06  1.43E-06  1.43E-06  1.51E-06  1.52E-06  1.53E-06  1.54E-06  1.54E-06  1.57E-06  1.61E-06  1.65E-06  1.69E-06  1.69E-06  1.70E-06  1.71E-06  1.74E-06  1.77E-06  1.77E-06  1.78E-06  1.81E-06  1.82E-06  1.85E-06  1.90E-06  1.91E-06  1.92E-06  1.92E-06  1.94E-06  1.97E-06  1.98E-06  2.09E-06  2.10E-06  2.11E-06  2.13E-06  2.13E-06  2.16E-06  2.18E-06  2.19E-06  2.22E-06  2.34E-06  2.37E-06  2.40E-06  2.42E-06  2.47E-06  2.48E-06  2.50E-06  2.50E-06  2.54E-06  2.55E-06  2.55E-06  2.56E-06  2.56E-06  2.58E-06  2.60E-06  2.61E-06  2.63E-06  2.64E-06  2.66E-06  2.66E-06  2.70E-06  2.72E-06  2.86E-06  2.97E-06  2.99E-06  3.11E-06  3.16E-06  3.20E-06  3.23E-06  3.23E-06  3.24E-06  3.27E-06  3.29E-06  3.33E-06  3.36E-06  3.41E-06  3.48E-06  3.49E-06  3.50E-06  3.50E-06  3.63E-06  3.66E-06  3.83E-06  3.86E-06  3.95E-06  3.98E-06  4.09E-06  4.10E-06  4.29E-06  4.32E-06  4.33E-06  4.33E-06  4.37E-06  4.50E-06  4.58E-06  4.77E-06  4.78E-06  4.88E-06  4.95E-06  5.15E-06  5.17E-06  5.18E-06  5.23E-06  5.34E-06  5.36E-06  5.37E-06  5.46E-06  5.49E-06  5.52E-06  5.52E-06  5.53E-06  5.54E-06  5.57E-06  5.65E-06  5.69E-06  5.71E-06  5.74E-06  5.87E-06  5.88E-06  5.91E-06  6.12E-06  6.13E-06  6.15E-06  6.15E-06  6.24E-06  6.25E-06  6.36E-06  6.36E-06  6.56E-06  6.60E-06  6.76E-06  6.77E-06  6.77E-06  6.83E-06  6.92E-06  6.98E-06  7.00E-06  7.12E-06  7.24E-06  7.39E-06  7.41E-06  7.41E-06  7.60E-06  7.62E-06  7.67E-06  7.80E-06  7.88E-06  7.91E-06  8.14E-06  8.19E-06  8.32E-06  8.41E-06  8.43E-06  8.54E-06  8.61E-06  8.63E-06  8.83E-06  9.04E-06  9.31E-06  9.32E-06  9.42E-06  9.59E-06  9.76E-06  9.86E-06  1.00E-05  1.00E-05  1.00E-05  1.02E-05  1.02E-05  1.02E-05  1.03E-05  1.03E-05  1.04E-05  1.04E-05  1.05E-05  1.05E-05  1.05E-05  1.07E-05  1.07E-05  1.08E-05  1.08E-05  1.09E-05  1.09E-05  1.10E-05  1.10E-05  1.11E-05  1.13E-05  1.14E-05  1.26E-05  1.26E-05  1.27E-05  1.27E-05  1.28E-05  1.30E-05  1.34E-05  1.34E-05  1.35E-05  1.36E-05  1.37E-05  1.39E-05  1.44E-05  1.45E-05  1.46E-05  1.48E-05  1.49E-05  1.49E-05  1.50E-05  1.50E-05  1.50E-05  1.52E-05  1.52E-05  1.55E-05  1.57E-05  1.60E-05  1.65E-05  1.66E-05  1.67E-05  1.68E-05  1.72E-05  1.72E-05  1.74E-05  1.75E-05  1.75E-05  1.76E-05  1.82E-05  1.82E-05  1.84E-05  1.89E-05  1.91E-05  1.95E-05  2.00E-05  2.02E-05  2.03E-05  2.03E-05  2.05E-05  2.07E-05  2.07E-05  2.08E-05  2.09E-05  2.10E-05  2.11E-05  2.12E-05  2.13E-05  2.16E-05  2.19E-05  2.20E-05  2.23E-05  2.24E-05  2.24E-05  2.25E-05  2.32E-05  2.33E-05  2.35E-05  2.35E-05  2.36E-05  2.38E-05  2.40E-05  2.45E-05  2.45E-05  2.51E-05  2.52E-05  2.54E-05  2.56E-05  2.58E-05  2.59E-05  2.60E-05  2.60E-05  2.61E-05  2.62E-05  2.63E-05  2.64E-05  2.67E-05  2.71E-05  2.71E-05  2.77E-05  2.78E-05  2.80E-05  2.82E-05  2.87E-05  2.89E-05  2.93E-05  2.94E-05  2.98E-05  3.03E-05  3.03E-05  3.03E-05  3.03E-05  3.07E-05  3.11E-05  3.17E-05  3.18E-05  3.21E-05  3.22E-05  3.27E-05  3.29E-05  3.30E-05  3.30E-05  3.32E-05  3.32E-05  3.34E-05  3.43E-05  3.43E-05  3.49E-05  3.49E-05  3.49E-05  3.50E-05  3.59E-05  3.59E-05  3.59E-05  3.66E-05  3.67E-05  3.71E-05  3.73E-05  3.88E-05  3.90E-05  3.92E-05  3.98E-05  4.02E-05  4.06E-05  4.11E-05  4.14E-05  4.15E-05  4.29E-05  4.33E-05  4.37E-05  4.37E-05  4.40E-05  4.41E-05  4.44E-05  4.47E-05  4.54E-05  4.63E-05  4.66E-05  4.67E-05  4.67E-05  4.72E-05  4.72E-05  4.74E-05  4.78E-05  4.79E-05  4.82E-05  4.82E-05  4.83E-05  4.87E-05  4.90E-05  4.93E-05  4.97E-05  4.97E-05  4.98E-05  5.02E-05  5.02E-05  5.05E-05  5.06E-05  5.07E-05  5.12E-05  5.14E-05  5.16E-05  5.17E-05  5.21E-05  5.23E-05  5.27E-05  5.27E-05  5.28E-05  5.30E-05  5.31E-05  5.35E-05  5.41E-05  5.44E-05  5.44E-05  5.47E-05  5.53E-05  5.66E-05  5.80E-05  5.81E-05  5.87E-05  5.87E-05  5.93E-05  6.11E-05  6.32E-05  6.34E-05  6.35E-05  6.41E-05  6.41E-05  6.51E-05  6.58E-05  6.60E-05  6.61E-05  6.72E-05  6.75E-05  6.80E-05  6.93E-05  6.95E-05  6.95E-05  6.98E-05  6.98E-05  7.02E-05  7.12E-05  7.14E-05  7.16E-05  7.18E-05  7.20E-05  7.23E-05  7.31E-05  7.38E-05  7.40E-05  7.49E-05  7.59E-05  7.74E-05  7.77E-05  7.78E-05  7.84E-05  7.86E-05  7.88E-05  7.92E-05  7.97E-05  8.14E-05  8.15E-05  8.18E-05  8.18E-05  8.21E-05  8.21E-05  8.22E-05  8.23E-05  8.25E-05  8.27E-05  8.33E-05  8.35E-05  8.35E-05  8.37E-05  8.42E-05  8.47E-05  8.50E-05  8.50E-05  8.52E-05  8.53E-05  8.55E-05  8.58E-05  8.62E-05  8.67E-05  8.71E-05  8.74E-05  8.75E-05  8.80E-05  8.83E-05  8.91E-05  8.95E-05  9.01E-05  9.01E-05  9.15E-05  9.18E-05  9.24E-05  9.27E-05  9.46E-05  9.52E-05  9.53E-05  9.53E-05  9.54E-05  9.59E-05  9.66E-05  9.80E-05  9.82E-05  9.85E-05  9.86E-05  9.87E-05  9.88E-05  9.96E-05  9.96E-05  9.96E-05  0.000100268  0.000100564  0.000101152  0.000101377  0.000102677  0.000102787  0.00010337  0.000103597  0.000103664  0.000104001  0.000105072  0.000105097  0.000106449  0.000107796  0.000107994  0.000108014  0.00010817  0.000108305  0.000109135  0.000109139  0.000110817  0.000112273  0.000112695  0.000112801  0.000112818  0.000114226  0.000114319  0.000114491  0.000114965  0.000115206  0.000115631  0.00011583  0.000116273  0.000116396  0.00011685  0.000119743  0.000120132  0.000120386  0.000121481  0.000121553  0.000124112  0.000124395  0.000125981  0.000127769  0.000127935  0.000128052  0.000128489  0.000128493  0.000130222  0.00013246  0.000133749  0.000135377  0.000136066  0.000136295  0.000137944  0.000138597  0.000139048  0.000139722  0.000140232  0.000140904  0.000140904  0.000141602  0.000141936  0.000142497  0.000144179  0.000144594  0.000144915  0.000145159  0.000145532  0.00014555  0.000145836  0.000147306  0.000148099  0.000148463  0.000148841  0.000149616  0.000150688  0.000150847  0.000152361  0.000153404  0.000154639  0.000155095  0.000156714  0.00015761  0.000158407  0.000159247  0.000160707  0.00016125  0.000162413  0.000163031  0.000164401  0.000165528  0.000166495  0.000167894  0.000168087  0.000169269  0.00016957  0.000169688  0.000173099  0.000174902  0.000177094  0.000178772  0.000180262  0.000180523  0.000180615  0.000180925  0.00018209  0.000182956  0.000184812  0.000188778  0.000189636  0.000190626  0.000190823  0.000191024  0.000191604  0.0001917  0.000193661  0.000193764  0.00019455  0.000195117  0.000195174  0.000197707  0.000199876  0.000200201  0.000201631  0.000205024  0.000205328  0.000205473  0.000206575  0.000206763  0.000208168  0.000208704  0.000210073  0.000211751  0.000212231  0.00021457  0.000216895  0.000217002  0.000218209  0.000221125  0.000221193  0.000221494  0.000222581  0.00022386  0.000224048  0.000225162  0.000226715  0.000227224  0.000229116  0.000229255  0.000230339  0.000230842  0.000232199  0.00023257  0.000233126  0.000233394  0.000234268  0.000234284  0.000235245  0.000236121  0.000238427  0.000238729  0.000240026  0.00024688  0.000247387  0.000248358  0.000249048  0.000249135  0.000249804  0.000252812  0.000255829  0.000256186  0.00025661  0.00025816  0.000260733  0.000263191  0.00026377  0.000264214  0.000264874  0.000265197  0.00026584  0.000267289  0.00026781  0.000269575  0.000269766  0.000269797  0.00027201  0.000273319  0.00027591  0.000275985  0.000276609  0.00027758  0.000279537  0.000279807  0.000282136  0.000282254  0.000284826  0.000285851  0.000286859  0.000286948  0.000290748  0.000291296  0.000294231  0.000296503  0.000297178  0.000297628  0.000298398  0.000298678  0.000299001  0.000301703  0.000301708  0.000301899  0.000302583  0.000303332  0.000303797  0.00030462  0.000305166  0.000309509  0.0003097  0.000312443  0.000314363  0.000316723  0.000318724  0.000323025  0.000323516  0.000323854  0.000325248  0.000325552  0.000325882  0.000327289  0.000330206  0.000332936  0.000333398  0.00033486  0.000335981  0.00033915  0.000339969  0.000340316  0.000340408  0.000341581  0.000343468  0.000347832  0.000348035  0.00034897  0.000352712  0.000353185  0.0003536  0.000356491  0.000358436  0.000359975  0.000360247  0.000362988  0.000368183  0.000368322  0.000368516  0.000369669  0.000369811  0.000371542  0.00037242  0.00037396  0.000377077  0.000380009  0.000381272  0.000383852  0.000385681  0.000386351  0.000389726  0.000392729  0.00039277  0.000396215  0.000398646  0.000403555  0.000403996  0.000405825  0.000406586  0.000409094  0.000409569  0.000410321  0.000411553  0.000412511  0.000412646  0.00041493  0.000416118  0.000416328  0.000416352  0.000416606  0.000416708  0.000417809  0.000421642  0.000422363  0.000423711  0.000425538  0.000428858  0.00043551  0.000437024  0.000437196  0.000437357  0.000443286  0.000449334  0.00044973  0.000450236  0.000451798  0.000452151  0.000458225  0.000461966  0.00046268  0.000463191  0.00046334  0.000464717  0.000465418  0.000466492  0.000467377  0.000469641  0.000470737  0.000474906  0.000481067  0.000481435  0.000484049  0.000485926  0.000487414  0.000487701  0.000487803  0.0004892  0.000489395  0.000491091  0.000491474  0.000495767  0.000496476  0.000497939  0.000501127  0.000502361  0.000507564  0.000511204  0.000514014  0.000514802  0.000516125  0.000516532  0.000518584  0.000518627  0.000521995  0.000523529  0.00052405  0.00052596  0.000525966  0.000531556  0.000533145  0.00053617  0.000542745  0.000543281  0.000545451  0.000545747  0.000546753  0.00054678  0.00055094  0.000554101  0.00055485  0.000558571  0.000560061  0.000560752  0.000562215  0.00056294  0.000566742  0.000569344  0.000579666  0.00057975  0.000580565  0.000580639  0.000581077  0.000581952  0.000583422  0.00058436  0.000584744  0.000587625  0.000588024  0.000591528  0.000596051  0.000598997  0.00060029  0.000600751  0.00060192  0.000602372  0.000602485  0.000607244  0.000608325  0.000609801  0.000615457  0.000617497  0.00061781  0.000619656  0.000628507  0.000635564  0.000635628  0.000638906  0.000639412  0.000643664  0.000645892  0.000648152  0.000651155  0.000651466  0.0006569  0.000658655  0.000660238  0.000668761  0.000669087  0.000670807  0.000671284  0.00067521  0.00067803  0.000683588  0.00068942  0.000690741  0.000690779  0.000697974  0.000701154  0.000718514  0.000720577  0.000720676  0.000723546  0.000729601  0.00072965  0.000731749  0.000734893  0.000735105  0.000737719  0.000739628  0.000742795  0.000745468  0.000748313  0.000749736  0.000750909  0.000755635  0.000756543  0.000762715  0.000764481  0.000764578  0.000765869  0.000770357  0.000771802  0.000776078  0.0007792  0.000783372  0.00078558  0.000792241  0.000794116  0.000798037  0.000800671  0.000801194  0.000802628  0.000802698  0.000804681  0.000805766  0.000807442  0.000810462  0.000812218  0.000821722  0.000824589  0.00082506  0.00082602  0.000830217  0.000833569  0.000847132  0.00084891  0.000849449  0.000849788  0.000851974  0.000858265  0.000859149  0.000865904  0.000868592  0.000869197  0.000869907  0.00088113  0.000883696  0.000891093  0.00089266  0.000895993  0.000896289  0.000899032  0.000899235  0.000903066  0.000903703  0.000903877  0.000904881  0.000908901  0.000911061  0.000915192  0.00091737  0.000920495  0.000927816  0.000929622  0.000944611  0.000945066  0.000946428  0.000949374  0.000951538  0.000954156  0.000955689  0.000957233  0.000957713  0.000962601  0.000963415  0.000967593  0.000970892  0.000973147  0.000973247  0.000973827  0.000977484  0.000988032  0.000993497  0.000995318  0.000997174  0.000997902  0.000998109  0.001000638  0.001001673  0.00101019  0.001015224  0.001017882  0.001018065  0.001019548  0.001019571  0.001020285  0.001023076  0.001023809  0.001024449  0.00102598  0.001030869  0.001034064  0.001035441  0.001042534  0.001053082  0.001060962  0.001063495  0.001066725  0.00107566  0.001080405  0.001080878  0.001084345  0.001084981  0.001089269  0.00108959  0.001093189  0.001093241  0.001102948  0.00110757  0.001109505  0.001114148  0.001119103  0.001124056  0.001131315  0.00113216  0.00113503  0.001141189  0.001143075  0.001146766  0.001150404  0.001151813  0.001153082  0.001171709  0.001175655  0.001177398  0.001185219  0.001185773  0.001191388  0.001200009  0.001202469  0.001206346  0.001211157  0.001213743  0.001217767  0.001220183  0.001226017  0.001253854  0.001254405  0.001254691  0.001264094  0.00126855  0.001272653  0.001277744  0.001282658  0.001291443  0.001298052  0.001299687  0.001302413  0.001309906  0.001309965  0.001313724  0.00131774  0.001319533  0.001319855  0.001326786  0.001333467  0.001333605  0.001334331  0.001335794  0.00133944  0.001345485  0.001347343  0.001356038  0.001356134  0.001357866  0.001364388  0.001365224  0.001372804  0.001374954  0.001377824  0.001379055  0.001381836  0.001387621  0.001390573  0.001392176  0.001396009  0.001401034  0.001414415  0.001416344  0.001418789  0.001420036  0.001428252  0.00143429  0.001437934  0.001439535  0.001443447  0.001444152  0.001451337  0.001452293  0.001453163  0.001453723  0.001454523  0.001465694  0.001469546  0.001475313  0.001476255  0.001479425  0.001490564  0.001490924  0.001494484  0.001508882  0.001511788  0.001513112  0.001516619  0.001520403  0.001520498  0.001521603  0.001524653  0.001547709  0.001548405  0.001553669  0.001564126  0.001566014  0.001567499  0.001570396  0.001574824  0.001579176  0.00158679  0.001590821  0.001594235  0.001595955  0.001596044  0.001596457  0.001596906  0.001601582  0.001609348  0.00161068  0.001613696  0.001621698  0.001628278  0.001631025  0.001644179  0.0016517  0.001663486  0.00166753  0.0016934  0.001702787  0.001705352  0.001706378  0.001711105  0.001713394  0.001716864  0.001718581  0.001723675  0.001724524  0.001729423  0.001733499  0.001753416  0.001759129  0.00177262  0.00177375  0.001779356  0.001780603  0.001785013  0.001797086  0.001801158  0.001805478  0.001811454  0.001814392  0.001819761  0.001829591  0.001829669  0.001837906  0.001841127  0.001845705  0.00184601  0.001874566  0.001880759  0.001883705  0.001887626  0.001888006  0.001888506  0.001892518  0.00189595  0.001910593  0.001915465  0.001919003  0.00192609  0.001929162  0.00193574  0.001938537  0.001940121  0.001947185  0.001955338  0.001978672  0.002007277  0.002020315  0.002020906  0.002022599  0.002035415  0.002042592  0.002047901  0.002054191  0.002056222  0.002056997  0.002058579  0.002061797  0.002068517  0.002070192  0.002078228  0.002078396  0.002086517  0.002098607  0.002101748  0.002102776  0.002105845  0.002107023  0.002108874  0.002109007  0.002109304  0.002123048  0.002128865  0.002129785  0.002139146  0.00215647  0.002160976  0.002162523  0.002164831  0.00216721  0.002170499  0.002185872  0.00219239  0.002198247  0.002201711  0.002217021  0.00221949  0.002224424  0.00222719  0.002227674  0.002231991  0.00223275  0.002233876  0.00223463  0.002235528  0.002243758  0.002244766  0.002251916  0.002261395  0.002277466  0.002283071  0.002308304  0.002313295  0.002322245  0.002335986  0.002340929  0.002344714  0.002344926  0.002350908  0.002354367  0.002354713  0.002356159  0.002359418  0.002361243  0.002363057  0.002367253  0.002370496  0.002372927  0.002375365  0.002380577  0.002390637  0.002393153  0.002396477  0.002402416  0.002405412  0.002407421  0.00241496  0.002416047  0.002419159  0.002420518  0.002424035  0.002425451  0.002434506  0.002439203  0.002457798  0.002458086  0.002460008  0.002460677  0.002464093  0.002471861  0.002475667  0.002482128  0.00248732  0.002494612  0.002507091  0.002509852  0.002517765  0.002520342  0.002526528  0.002526891  0.002532937  0.002545985  0.002554915  0.002559089  0.002568135  0.002571702  0.002580775  0.002590985  0.002592708  0.002593069  0.002603412  0.002603946  0.00260883  0.00261292  0.002614653  0.002617367  0.002631955  0.002639064  0.002648291  0.002654346  0.002669496  0.002688477  0.002711412  0.002713187  0.002714769  0.002719556  0.002721122  0.002723059  0.002726037  0.002728997  0.002732755  0.002733534  0.002744374  0.002745276  0.002747939  0.00275177  0.00276957  0.002776245  0.002778835  0.002782414  0.002794934  0.002807477  0.002812649  0.002827969  0.002835613  0.002851293  0.002868822  0.002868877  0.002871971  0.002888685  0.002893028  0.00289648  0.002897106  0.002904589  0.002905155  0.002922218  0.002926009  0.002938573  0.002939256  0.002941377  0.002949498  0.002949713  0.002952854  0.002961406  0.00297065  0.002974777  0.002980231  0.002980505  0.002987682  0.003002378  0.003022048  0.003026467  0.003026557  0.003034895  0.003039248  0.003039837  0.00305063  0.003050774  0.003050865  0.003065209  0.003070629  0.003074194  0.00307683  0.003081864  0.003096004  0.003097323  0.003104682  0.003114918  0.003119487  0.003124404  0.003134559  0.003137051  0.003140749  0.003148554  0.003191475  0.003206255  0.003211572  0.003219703  0.003223551  0.003228969  0.003240843  0.003243417  0.00324758  0.003247661  0.003273162  0.003281311  0.003290207  0.003298063  0.003301853  0.003303843  0.003309528  0.003312761  0.003330303  0.003349674  0.003352498  0.003354438  0.003362065  0.003362188  0.003368582  0.003377267  0.00337739  0.003381595  0.003383408  0.003383857  0.003384403  0.003390437  0.003392194  0.003392215  0.003404606  0.003412928  0.003426403  0.003430835  0.003433589  0.003439242  0.003440487  0.003458575  0.003468062  0.003478438  0.003493885  0.003504358  0.003507004  0.003511748  0.003531481  0.003532041  0.003536154  0.003536257  0.003537196  0.0035399  0.003545638  0.003547055  0.003549655  0.003552632  0.003556377  0.00355791  0.003559736  0.003567945  0.003571622  0.003594923  0.003604241  0.003613837  0.003617293  0.003617434  0.00361805  0.003623813  0.00362468  0.003625246  0.003626936  0.003638408  0.00364111  0.00364273  0.003650986  0.003670533  0.003672173  0.003686282  0.003689734  0.003712313  0.003714378  0.003715215  0.003730914  0.00373435  0.003736479  0.003751219  0.003787654  0.003790975  0.003795872  0.003796738  0.003807598  0.003813075  0.003813246  0.00382514  0.003831368  0.003834427  0.003836121  0.003847528  0.003865224  0.003870192  0.00387501  0.003876927  0.003885707  0.003890073  0.003890269  0.00391977  0.003922803  0.003945489  0.003952379  0.003956418  0.003982088  0.003991963  0.00399764  0.004001152  0.004044593  0.0040655  0.004078225  0.004089248  0.004090596  0.004102207  0.0041112  0.004113048  0.004123036  0.004133717  0.00413871  0.004174358  0.004181501  0.004189305  0.004194042  0.004205044  0.004209525  0.004210516  0.004222114  0.004224114  0.00423653  0.004255607  0.004256766  0.004272098  0.004275633  0.004289331  0.004297699  0.004300147  0.00430195  0.004315265  0.004320624  0.004339429  0.004342856  0.004353465  0.004355714  0.00437023  0.004381834  0.004400657  0.004400956  0.004406309  0.004422994  0.004430834  0.004435586  0.004453106  0.004454738  0.004460434  0.004463701  0.004497978  0.004522502  0.004530944  0.00456026  0.004569541  0.004579299  0.004584226  0.004585938  0.004621587  0.004632686  0.004636724  0.004641382  0.004641541  0.00465319  0.004660536  0.004661591  0.004668995  0.004669934  0.004699337  0.004707695  0.004709559  0.004718745  0.00473987  0.004747829  0.004756917  0.004759763  0.004771502  0.004778525  0.004778611  0.004785568  0.004796915  0.004821352  0.004828786  0.004830879  0.004846495  0.004857216  0.004858502  0.004860999  0.004871294  0.004886012  0.004893643  0.004910242  0.004913695  0.004924344  0.004941024  0.004942055  0.004949298  0.004977096  0.004978498  0.00498032  0.004981187  0.004988198  0.00501742  0.005025812  0.00504303  0.005057584  0.005070721  0.005099371  0.005105155  0.005110455  0.005117243  0.00513634  0.00514111  0.005142898  0.005147317  0.005150352  0.005156756  0.005171451  0.005173677  0.005179887  0.005204326  0.005206515  0.005208708  0.005216697  0.005221165  0.00522666  0.005238006  0.005239779  0.005239902  0.005247571  0.005257053  0.005259079  0.005270699  0.005299586  0.005322238  0.005324236  0.005370529  0.005378522  0.005388338  0.005401162  0.005435194  0.005436395  0.005447781  0.005449278  0.005484437  0.005489686  0.005503164  0.005512011  0.005516841  0.005528227  0.005553697  0.005571918  0.005580573  0.005591455  0.005596218  0.005607624  0.005618808  0.005622217  0.005663885  0.00566722  0.005695887  0.005710875  0.005783413  0.005791904  0.005792179  0.005799081  0.005817697  0.005831334  0.00583717  0.005861264  0.005889676  0.005901367  0.005912243  0.00592799  0.005958324  0.005991952  0.005993644  0.00600274  0.006002905  0.006013166  0.006028908  0.006051569  0.006055862  0.006062144  0.006071055  0.006081384  0.006083444  0.00609655  0.006101254  0.006105828  0.00612095  0.006125304  0.006126603  0.006132217  0.006155024  0.006161421  0.006173841  0.006184915  0.006245569  0.006247576  0.006254213  0.006280298  0.006286638  0.006289247  0.006313427  0.006323781  0.006362975  0.006371599  0.006375206  0.006386493  0.006395784  0.006420904  0.006427406  0.006439759  0.006449133  0.006452651  0.006454909  0.006459875  0.006466563  0.006489015  0.006492892  0.006498049  0.006506675  0.006520266  0.006522013  0.00653313  0.00654244  0.006547257  0.006547785  0.006550121  0.006580586  0.006594831  0.006595365  0.006639786  0.006647122  0.00667479  0.006676315  0.006679325  0.006700695  0.006701133  0.006701932  0.006706283  0.006708181  0.006728982  0.006732412  0.006759918  0.006777798  0.006787726  0.006790876  0.006794528  0.006800762  0.006812863  0.006828117  0.006864723  0.006870097  0.006873699  0.00688011  0.006884649  0.006889225  0.006896462  0.006897233  0.006901821  0.00690734  0.006908802  0.006937351  0.006938853  0.006953875  0.006959279  0.006994497  0.007009488  0.007039073  0.007064268  0.007064575  0.007085821  0.00709516  0.007108512  0.007127463  0.007135297  0.007140608  0.007155541  0.007182599  0.007184983  0.007215249  0.007232477  0.007236032  0.007249651  0.007255513  0.007261846  0.007287832  0.007298108  0.007335953  0.00736134  0.007370787  0.007386767  0.007438101  0.007454029  0.007454652  0.007462027  0.007479013  0.007489979  0.007499125  0.007504675  0.007514862  0.007530312  0.007533053  0.007538791  0.007583667  0.007627208  0.007628687  0.007635902  0.007646328  0.00765763  0.007662941  0.007672463  0.007683762  0.007686627  0.007690079  0.007695781  0.0077056  0.00771623  0.007722767  0.007742787  0.007769021  0.007795344  0.007811262  0.007813278  0.007819252  0.007825472  0.007828604  0.007834665  0.00783746  0.007840211  0.007841093  0.007903523  0.007917989  0.007923699  0.007936778  0.007938384  0.00796952  0.007998151  0.008028048  0.008045462  0.008050507  0.008092362  0.008092393  0.008093851  0.008095897  0.008112929  0.008144254  0.008152204  0.008158311  0.008163598  0.008168953  0.008169962  0.008176252  0.008187225  0.008220844  0.008252133  0.00830285  0.00832171  0.008322537  0.008332495  0.008346002  0.00834961  0.008372835  0.008378028  0.008423765  0.008426297  0.008429398  0.008433434  0.00845737  0.008479132  0.008529404  0.008536008  0.008566771  0.008588451  0.00859982  0.008621075  0.008642461  0.008661885  0.008695883  0.008702364  0.008736667 | 9.300189913  1.57798133  6.758879687  8.646212489  5.151913784  7.510032478  10.87127853  29.99033508  3.961769177  3.741288897  2.95664832  2.950617871  2.291937318  5.016100289  5.209769649  6.952179179  28.02887589  4.104101233  2.463099721  7.497113091  3.376342449  3.420609969  3.053647599  2.744266996  24.99325417  3.482221763  6.012174735  2.557283745  25.00387409  12.2541418  8.196710743  1.142204467  20.50931034  25.89148254  38.30302412  9.224294933  5.147757653  12.6709688  26.47887626  3.283961043  2.35432181  18.88418143  2.322942663  2.582113721  6.162189641  4.653562772  10.33256055  10.54238299  7.771046263  3.918852778  4.775469125  2.11870265  15.19947486  1.217627985  2.262601367  3.921987971  2.001719518  1.816298781  25.27718431  15.16968445  4.66187893  5.307191655  5.708618821  2.411306124  3.444556107  24.58084315  7.535827411  16.42639624  1.591691709  4.044918401  3.043422482  3.058667715  2.113511297  3.060811549  13.4749725  2.248345616  1.907961585  1.659457203  4.610411345  2.628266371  6.302150158  4.010773059  2.389938691  2.779961919  5.918779308  1.669606827  5.536258324  4.319886843  2.352377021  3.275736248  11.85937881  1.234477367  17.59580264  14.82838791  11.80415913  53.78315062  2.604448108  19.74209864  6.442195824  8.732281774  4.183031156  7.072693503  5.287005791  8.080001417  1.229180497  4.26706315  2.60451479  7.951308612  1.734443211  1.916293593  19.31999658  73.99658232  5.156842504  2.897502881  5.647915503  24.60656297  4.947240296  10.89547549  11.44330678  2.833839248  5.370119232  6.831059408  3.53241635  12.96378145  3.092660572  20.43171762  7.016156974  4.410984365  1.095757255  32.90664728  4.097791862  2.086937352  31.58286929  3.290089896  10.09600701  1.211911731  6.81540948  3.147394474  20.65653656  4.056069855  4.592308712  10.33511181  10.03876717  2.775958059  1.733786308  1.511598878  3.460766703  3.110454099  3.54531597  3.556860472  1.161423394  9.084585849  2.540064748  76.39273217  7.444962817  1.848584147  8.392173046  5.915332292  1.982445921  2.020462933  3.964363704  2.155795236  2.104336493  16.28766119  9.008024012  55.71859382  2.626049561  2.691074634  2.180433416  6.316552433  1.359875999  6.950114456  7.700378217  2.287442481  5.26018247  10.08677728  5.490681216  1.695707706  16.99675579  4.184256178  1.95830858  35.61981877  22.7416371  2.46817555  3.523854321  9.575351242  1.101611592  24.12023111  3.428848354  8.268820246  1.659989208  7.004800968  3.72318182  2.860209302  7.064172405  4.062945205  4.062358202  1.582511829  10.09602962  3.387478628  17.55476173  20.1458088  5.16429086  14.36330511  4.902223364  4.019899038  2.978540066  12.95925638  4.014885524  2.37552426  6.096104754  3.444371941  5.685403963  3.419501365  29.4832529  4.512320677  16.06076232  2.866948655  8.537318077  1.55216477  1.331347687  2.630605317  1.594952881  1.104804465  12.52242916  12.30682679  3.225673686  14.21626623  15.22138838  1.612724032  1.12100112  4.093882469  1.030329024  10.69963367  1.207278164  2.132525889  2.562989304  6.976322436  1.060286897  6.107184684  19.92404921  5.426200638  1.410972554  35.97882867  2.858913524  12.0584505  4.697866063  3.041056236  6.129092861  11.41065404  5.000237184  1.30179298  1.782222396  2.139233923  3.859648703  8.879445445  3.013971904  1.628002212  11.50539138  68.18119739  15.6819686  2.827366901  3.07183997  7.492770416  6.879118075  3.487406091  23.24663259  2.568315605  7.654045681  2.233374989  6.042306086  57.8077163  7.193125399  2.588720627  4.325604187  4.815940498  6.295698537  19.5697539  8.914961873  1.321264084  4.569758609  22.34862875  2.560905629  28.79394466  1.581057892  1.8752509  12.93377355  1.684990525  5.051518199  1.085097071  3.438819364  9.420635862  25.8608362  7.852483375  2.100106045  2.473825787  10.54858892  12.82976788  10.43531958  2.918676568  19.35383925  2.024389334  37.4518859  31.3852639  3.143656328  106.3072121  1.182090756  2.487368032  8.364690446  2.075554315  5.3392905  1.121679715  2.663460729  5.767615595  3.210960097  4.891138779  9.090503949  6.456698349  2.323504875  8.25117071  2.05656321  24.00323182  3.037998538  7.732180972  2.37240448  8.379342671  9.19598531  2.402309342  10.55912836  1.204819167  11.45327231  1.156156986  2.327700958  2.099629398  1.767100432  1.57748127  6.346490581  13.790273  10.77682805  1.35276173  2.86075286  17.46580967  37.79504118  6.889681759  6.59432855  1.879989581  4.239258162  4.93304142  6.895271752  5.043806791  5.236570274  1.841679118  5.778811651  5.892125533  4.912743973  14.42462158  1.176198874  5.734523671  137.180507  1.176226594  3.712557879  77.73271163  1.764043663  1.778337186  3.282911595  18.47180662  3.693692668  19.19824085  1.827218543  1.183785606  2.299874218  4.098384894  12.54929912  4.099877193  26.33723171  2.329273317  1.397106257  14.643942  3.659407066  1.104984472  3.541553194  2.674327813  7.193731787  1.418373806  21.65436294  3.62465477  4.257629827  4.207552701  15.23789928  2.485520365  18.84940122  27.63612505  6.998810453  2.285863128  2.16909885  11.30910543  5.02449918  3.857055379  2.156747716  1.469460988  19.4526225  1.18609567  4.084844945  2.089703587  11.87109058  26.27320827  7.311758846  1.997759381  2.676600405  4.281665806  3.843200535  1.365590301  19.78661384  12.14995632  3.786738035  49.20898049  7.906608884  2.895929912  2.519273561  4.418920665  1.996210389  9.88520821  3.151834699  16.1874143  4.229791904  8.574439252  2.636956811  1.506472518  1.406673855  22.24958728  3.281315881  4.552202657  11.9593008  20.82532037  2.746239475  4.213168573  29.00078199  3.360644698  12.17358656  31.20321955  17.22721982  4.800802833  1.063986216  25.70641506  6.628561613  8.642244076  1.357129933  65.61184184  2.641995059  1.31800139  1.794988483  13.90281325  4.924450787  4.388892815  8.269237745  2.053078642  9.801341996  1.322010536  13.1662742  100.3862806  3.780584647  2.001516076  2.276598888  1.865319134  7.335811863  8.566549764  11.09591494  8.558493518  111.8462644  7.176417099  11.05037963  18.20121589  6.585852614  21.17518737  1.346919925  6.123405459  3.146399184  1.374045702  6.273674008  65.89117982  5.537393455  4.133542563  1.735245481  1.53352217  19.23736479  12.67816773  7.058644955  4.312211994  116.8764303  9.073651534  2.574496823  1.477433395  2.620671704  18.1637887  1.946039955  3.452186261  7.480647687  2.448975195  9.056401788  2.038677617  4.827332631  1.513516717  16.95414179  13.20242232  4.418605297  5.680070158  3.208942569  2.178683995  15.48854669  3.944822574  6.365244942  27.15025097  2.477627394  2.764138457  6.61052793  7.958410348  3.248161988  9.320087188  4.704857518  13.43112877  3.214470978  21.99277983  2.275597275  4.23068033  33.69750384  32.5696619  42.61043058  7.934136176  9.496463141  4.864133483  9.898554659  18.34979485  2.197670972  6.083923933  21.1167701  74.05938594  1.627604705  2.364015045  1.262160688  1.987983033  14.85127655  3.493947699  11.51855094  280.9617341  7.10650756  5.005237976  4.833229576  18.93761801  6.933585142  4.66561004  7.090298703  14.47563707  117.6164754  55.88446602  16.64287776  1.54037727  1.312659851  6.564800607  1.146863489  1.790597937  1.016261108  4.762588029  59.23429375  2.174923134  6.480175908  2.580674799  4.09497846  1.412966614  4.189383075  10.0360709  5.699859927  1.803570566  1.957298164  5.701588106  78.98793698  4.284812274  3.971781361  2.042135906  8.74542863  1.757013888  1.723737806  1.257091582  5.063943295  11.01570386  6.893144001  2.765395241  3.400166293  58.77409209  6.35373557  3.155299062  18.58527453  1.457188683  21.56703846  18.79030601  18.94682638  1.127563508  3.458187147  2.563280227  3.570542317  9.261839536  10.60817879  4.333416614  10.11023182  2.779868844  2.762047689  14.33521686  5.040879744  6.619217194  3.592360092  1.361342851  2.976308249  33.07280779  3.780070192  5.773909299  13.2381692  1.759771884  13.70719983  19.25852815  13.2105947  36.57964371  12.85863929  9.764334558  1.441038355  2.40858316  4.818742347  7.634170988  1.824696344  6.201963274  5.739307917  51.47526707  15.8180535  11.60905832  18.14006998  16.98549825  9.856784115  7.377865671  4.116033104  1.033673058  8.511766186  24.13559489  5.432589392  1.745741836  3.507669576  4.279885638  10.96487711  10.35190729  22.39006628  3.920867962  37.5205295  3.448731859  19.97725365  76.17700534  15.8099199  24.70814387  1.843025424  22.83147866  2.91809459  96.59992202  40.23204824  1.578066873  1.865260453  118.380256  10.15016283  19.44826898  11.29635471  15.08038328  15.67685026  3.638508569  72.54064156  1.857800871  3.257951335  21.48871704  13.40892346  11.62874893  5.377882609  8.090225389  3.837158559  77.62957508  1.536085994  13.07772673  6.342277091  10.94658084  2.334820652  2.057129129  1.162689293  10.49021503  1.962292652  5.583688839  30.1757411  6.87999194  85.96831475  4712.644277  8.477458891  20.53320077  53.1557909  10.31814165  6.421061043  12.19599245  6.10969179  17.9069934  8.637184244  5.347825908  5.107181574  5.001979041  5.484090261  10.46462396  80.14071947  1.435803221  1.858118436  10.25290645  5.711570857  9.551118209  3.017928556  4.526119797  23.08903552  1.451031652  7.437222464  15.13203764  82.14139544  5.160760192  3.384465245  3.527919341  4.832985793  5.714507495  25.08328724  13.27634455  3.496858284  3.016638907  5.082846454  2.71085028  13.51109433  2.919480095  141.9820031  4.924346765  29.70824885  8.773978608  2.130604458  3.379276495  1.449107224  21.41586499  6.281610589  32.15172746  1.989744309  2.15203542  2.771173229  2.834751345  5.689437107  599.9124396  80.60199509  12.91175314  1.91033517  10.94146359  1.543069787  2.271681662  18.90974528  1.311205143  5.205214872  10.40833478  2.027634572  79.93702481  1.405971761  24.06146245  2.785030678  1.46953628  52.85364284  3.305582031  6.48422491  12.79927683  5.049682745  27.05242234  14.01950518  9.539879598  5.67612957  3.89343473  23.83327497  6.921054097  8.391401871  5.272531559  16.39400254  1.027003017  49.94521501  27.4845535  135.6240053  54.47218406  61.8103956  11.67407983  1.097839984  16.74707657  5.428977163  5.594700798  4.7629897  71.92003132  6.200477785  3.952626671  2.809407138  3.48689718  8.353398592  3.021890931  2.893444781  8.002546165  55.35247503  13.35115985  2.003345401  1.461716838  2.495953348  2.234557203  50.08877132  1.755317605  22.02457506  68.07088592  7.940272964  4.105059246  8.074375107  1.077753815  1.257604912  12.06941111  7.42958854  4.530755131  1.538929199  5.631258858  3.929822838  10.9141643  7.69924811  33.40725647  7.86640769  3.226939562  1.447879561  61.22977169  70.15734187  3.338979828  7.65541191  5.80107984  1.186078029  1.634186741  19.70514913  5.627701687  4.526937094  25.86603057  37.78283896  6.091465004  3.742683125  4.703804777  28.24933851  1.554806153  1.394821768  4.162584901  17.1489864  7.313738744  1.215691364  10.56394618  2.946785839  2.302827493  19.69728741  17.01303263  5.58530005  2.40664284  14.06333806  2.612345433  1.904644089  28.09402732  6.669895811  8.033334116  1.260484583  7.648474014  69.30978915  12.07569421  16.57273918  7.296451476  17.30642279  1.877353417  90.49294322  5.504539737  1.050330408  3.523977179  14.74153752  5.998383972  7.207355838  1.898956034  6.956935289  39.64242942  2.535381159  6.644752108  8.130884405  1.351170169  136.0795975  8.777208241  25.58130963  17.40783444  1.003197218  5.973574137  2.244668217  7.339925115  1.39170943  43.5869191  11.57013914  1.01298098  9.537980765  1.577964401  6.835927511  19.70638189  9.454912431  143.2535627  2.511183405  8.350837435  24.31590714  13.90849289  23.00934772  2.990949381  1.137014146  168.2584602  1.734654832  7.522707655  4.1192816  14.28622966  23.94545946  53.72667732  3.658337504  14.99761699  20.3388811  22.49091076  8.968815497  16.18064584  6.499158493  130.3114593  9.013553642  3.934268797  8.758134022  2.509222196  2.147210857  241.6847534  4.64601883  6.257804243  42.33391774  6.435467573  5.670318501  4.268493142  33.77338869  2.334554564  15.19158439  14.10177757  36.29820678  7.370772678  5.185089363  3.11679486  6.303567768  2.919214693  19.73205053  3.313550624  6.226872622  5.616934506  32.68283081  1.407922813  1.15054014  6.222633785  7.092817644  2.944181677  7.121895999  5.288969376  5.792595864  3.853576224  16.96237704  7.478327381  21.35714305  24.99213233  1.23374137  2.209611359  4.277401811  769.3170588  6.667355011  4.950571439  9.464896554  1.392336466  9.286172651  1.943459067  0.959088602  5.921274601  8.158909093  36.26380594  33.89242819  17.72593412  1.460262406  13.45529311  61.17754805  33.61841424  41.05240582  7.980223222  5.932509155  11.47127559  74.09648082  8.919994167  1.39203687  5.709047646  7.998639213  2.598779959  3.461389939  8.856267574  196.1673574  62.62814606  8.398633045  6.846123893  195.3570156  13.96423676  5.74010006  8.538698024  5.633684622  11.21010921  67.13698423  17.72366295  4.274682596  1.81299247  28.78542971  2.299288632  7.640562671  1.715949489  7.801790839  2.107310511  18.02338836  5.59051615  5.607202608  10.34594697  19.52472194  6.482587265  64.09432184  23.19234241  5.418796887  5.513151502  44.70779198  1.769291499  5.56551025  3.963676082  1.669970528  1.085567851  3.217947895  17.13604093  1.273432712  1.469386946  4.757000558  8.17990988  4.412801276  4.631509243  11.51420077  2.565452944  5.622590731  13.61930954  7.318911514  1.829464165  5.366222475  20.73224722  4.597172517  390.860725  7.234806738  3.166193434  5.295489862  5.323493373  15.23878209  12.55373358  2.181367  24.65732164  47.09932244  7.298666733  18.3023099  16.7627408  5.271608823  13.35695209  10.58228099  8.531651805  29.99021866  55.14504764  10.65576869  2.170601736  1.235633175  42.63976971  4.054020942  7.696019856  2.293890089  21.44122614  29.58537719  1.41842182  2.55154826  5.93005058  114.324387  6.200112003  2.6737707  1.189718408  4.956843402  0.984160256  11.44779273  17.1175169  1.46537171  10.1036844  3.761582156  2.569306451  2.092334033  2.862050859  3.576319003  41.92190673  2.375762606  2.777173321  19.05424085  5.039468761  7.705285786  2.756235261  62.16284369  10.62493465  14.51148425  18.32579735  3.613874607  9.968889516  13.28218358  4.857048264  4.970606933  3.472554821  2.278667917  7.256935966  8.195492128  15.50334327  36.12377961  1.607590752  1.10152116  2.693431001  4.856419395  14.41245126  4.681428011  3.057522537  38.55237218  38.11789296  4.819042637  2.916368757  4.770173145  13.8499769  10.03096385  1.342128789  8.203448476  5.000264296  2.483173795  12.14466837  17.6295781  2.738905682  15.93565377  4.482979188  1.97888857  33.44453741  3.211121817  5.430055992  15.22080034  2.880237729  13.44636348  5.677801941  6.32961995  7.236566047  5.646132205  51.10954532  4.672820068  5.7994088  41.84808445  50.84177783  4.730107544  427.0861386  22.84324104  3.907955698  20.43661204  26.83738563  22.81476478  16.60669977  8.139390565  1.664767523  1.597485529  21.85033448  15.66303324  15.23534919  1.497121307  12.73300193  10.1821461  27.17226833  4.683692167  7.536284345  6.346654646  4.66901173  62.80462859  2.723639183  11.59063226  2.201251222  9.648771637  3.06254731  1.909841011  735.6880273  2.644456085  53.33981835  2.743489896  8.285718048  9.52415948  1.918468558  23.05759106  2.220074522  19.11209632  1.127478891  0.998827837  2.786525967  4.742332555  2.439447119  6.00560963  2.011986367  34.01061662  2.435234878  4.020723858  59.70247566  7.325447229  8.655958823  42.00095183  11.17348148  4.532042758  7.006154652  27.46332805  7.731985911  1.448122594  5.247141562  1.547059139  6.007258594  4.388626301  10.03245186  5.389334939  69.57507919  7.61207639  8.09748356  1.911397705  60.40801771  4.439101289  4.594426287  7.451962566  27.99955894  1.585074216  1.904792409  1.35530488  10.85402958  12.48630378  1.150277312  7.047116717  4.404190475  4.528523234  5.137231775  2853.917313  7.7962563  1.220971916  8.117788438  22.32092768  4.402598984  7.649697904  7.721599917  6.989282833  79.00242067  11.38877711  4.36233385  1.660946823  4.263227594  2.887526377  2.633884223  11.2782331  2.913887703  223.1063551  5.910310492  57.38196042  4.371393323  6.951731356  9.787719305  2.016883343  8.404316632  1.697412087  2.41135577  4.686151394  10.68598462  5.981674087  6.079517802  5.784327051  565.8633808  2.609474365  1.573076435  1.914496765  86.68606974  4.7743535  22.18264295  10.01057294  12.55148639  4.231511523  4.209936826  1.919974675  33.47122044  1.325170357  3.839458557  5.592907523  20.33353647  8.774968541  6.244846105  2.00807826  10.51796205  9.306813963  18.68361108  5.596104325  41.03723496  1.268426522  6.597391992  11.70497566  1.521601984  32.06183693  45.89381087  34.03215696  4.23979755  4.150470598  1.31674224  3.952955406  17.64289591  29.44843772  9.33229462  1.511047971  14.99247795  9.327433224  3.029849691  20.5651982  4.113461684  14.9974176  5.648254414  2.301483183  5.678420133  4.826499328  33.95268672  5.773421843  3.766457258  8.517634676  15.48414123  7.379725535  36.81718546  33.71655238  23.61679121  9.486369716  2.850139063  1.250422843  100.7600855  31.14909412  4.619943121  6.018935617  4.046875618  2.547259094  11.44586279  3.426424344  9.412062122  1.368806262  1.03824327  5.575432068  6.919523491  4.10338761  1.073338323  6.482412913  12.73550645  9.005472202  1.773730718  91.16508063  6.5665197  4.246210449  4.610582859  685.9247994  4.043823961  16.96853491  2.453721353  20.7910566  87.68164359  9.571044585  4.032039289  3.01051779  4.539990965  2.349646571  23.38827249  51.80226266  36.65192753  2.983020633  16.76494219  2.934711468  15.66359226  5.891050993  8.258388935  1.420969967  4.289617206  19.36358649  6.009273442  9.294626634  37.59794446  330.5283121  13.85915464  10.33140753  5.024188169  25.85189212  419.7621617  1.07248476  1.518503135  2.270472558  3.59563212  1.190800371  26.77308508  12.2974426  3.958363286  13.1133539  5.974682083  7.153016333  5.264495073  7.040896234  62.40212644  405.3782701  19.02289038  40.66638468  5.418766882  1.307834834  29.01227544  2.085580735  59.7216079  8.180678302  24.74281416  23.55418195  7.024362391  1.619668967  22.34687855  4.069831177  1.521405654  27.43314881  1328.265122  52.00979343  5.981558649  684.6167405  21.18589664  4.299594368  16.10263307  3.017951456  1.031547377  12.61627438  2.301582458  20.37054896  6.054902598  63.40452401  31.09026285  2.120847139  1.587581008  3.777296561  3.823668771  20.73635086  2.882926731  6.896274733  7.652415852  83.62982334  1.791711814  3.762751142  203.9092638  3.745280993  45.40947365  4.923955608  18.31844332  8.799361148  10.39703969  150.4300011  10.33890164  1.969381932  1.885574464  7.512380631  2.007839293  59.78552083  2.675903827  16.94647363  78.25873889  14.72672226  3.786403141  14.95208035  4.044430411  4.332446109  37.80403655  4.64310121  18.32241731  10.36438968  3.545102851  12.93746107  5.895536327  2.605700183  3.761447173  11.5237883  1.284392806  15.11463147  3.759133815  4.176116125  1.404812242  5.355998866  3.671608956  15.70839675  75.57178493  11.57420851  5.198910831  3.221082999  3.659142755  9.837491633  32.81180231  341.8875614  3.706695053  14.26193881  20.30530599  16.50612696  35.14699631  78.09519442  417.7652922  7.293865481  1.506150423  3.627499352  15.73102866  93.46759673  1.299940912  9.876563385  9.594358372  1.113249822  4.039522698  16.8986459  4.796906856  10.22125418  3.674618154  12.39529686  1.552128896  11.10761505  1.113557086  25.69802384  3.044208802  19.82153296  4.010692164  5.195258999  12.43352397  1.400190806  7.667863081  1.261632984  8.407260536  3.741139678  5.83800575  2.798564987  3.046174092  1.206773906  4.869090039  1.206378575  15.99869208  27.81397602  2.46722208  1.887761782  8.403914353  12.15798283  7.906804328  13.02598904  2.939959974  23.68277614  14.06125033  310.040073  3.337782111  8.615455432  3.531731222  11.00305233  2.835669015  35.07405792  7.119926553  0.991705626  3.581869965  93.46447275  20.63421102  16.60601406  9.467549224  3.526753645  2.099588434  1.044338941  1.222865777  53.7820042  2.810446039  21.51866672  5.601368665  7.477403827  18.07414922  1.584090991  3.542759918  20.25396603  3.698235936  9.521669309  25.77214633  4.876821747  16.98953727  3.964176405  11.42220202  9.381266936  4.46580357  1.483732142  145.402334  10.45075195  22.02169605  3.511880368  3.310675826  14.48551932  6.919848693  19.01344842  6.608234235  3.102322419  7.980670817  4.920310315  19.25348608  8.050203351  2.056967527  16.76012369  3.482273071  3.302488759  41.72128163  2.842134353  4.490109013  3.214551007  11.88133309  30.77663442  3.233686624  3.70019196  11.26451371  3.504022788  7.855315528  7.437299156  5.046273147  5.477628077  10.47254996  24.60660899  7.427298134  7.970614824  6.808598688  3.454105237  16.06203035  4.404721183  16.85342974  2.770032447  3.462659097  1.304832601  1.881085381  10.00541446  11.02591102  3.379333982  2003.718316  3.39148185  3.437699633  30.52433124  6.691187768  9.044708785  5.762103381  12.76830392  4.899919842  16.03777138  13.02750977  11.20536654  8.715000137  3.386142675  17.13443141  6.643761221  96.08668392  1.659009745  7.764624789  2.566376905  18.67495872  16.86553561  3.413547254  302.4195109  6.822761486  11.40402185  4.336543449  9.219563332  3.359608812  4.4230659  31.98578702  4.798525285  2.102989573  34.92153246  8.176110062  13.84331925  8.350373287  13.59302201  4.283244753  57.77142413  11.24982916  11.61565146  49.51950958  163.8993306  90.76720669  72.37099014  8.476381259  4.664369911  33.3230507  121.7307319  29.55123047  1001.45083  1.926451949  7.645840092  10.6215324  1.597686695  5.97993565  4.746463579  25.00610274  1.57807307  8.202722479  9.270040994  62.7375334  6.98346303  4.379314217  9.279209056  3.310126745  8.483438407  5.160267769  4.731350676  5.234290886  6.087046132  3.877901612  106.6552426  3.314809154  8.161383146  281.1203129  2.406585665  2.201341323  3.303445986  9.600277678  37.31836858  30.30207885  3.212046345  1.151833872  35.70352654  7.361862364  4.23895454  3.937044038  1.042303662  7.593906881  19.76119836  9.641785639  42.23050538  8.355306434  19.48987467  4.455819547  3.294329492  2.68509764  1.420541513  46.7062844  7.679419745  6.980779659  4.576709347  3.211336683  1.267782776  11.96351464  43.01426455  2.326642684  4.257282396  4.229285604  2.33479985  13.12133099  94.53863293  133.8121361  14.3826524  16.72189287  11.93370065  20.87964583  9.796855382  21.44109928  1.794154264  7.165681015  3.170300387  6.944547352  94.9701584  15.08787834  59.57913839  104.8169373  148.9702369  4.586886644  2.970904149  1.517515026  2.952353284  7.238812814  3.199656877  3.222598025  18.70326361  52.10743946  1.424511089  56.16947785  17.518591  1.468366124  48.15422279  3.180160617  17.45754876  26.30435468  6.303377512  87.42882386  8.403148029  4.439285268  17.21233431  6.117380286  88.52833657  124.3025411  12.71473549  7.70516509  2.464613975  9.559398004  12.22079404  1.345789972  9.510198358  3.705695811  7.300525784  3.157649861  19.96918623  14.79091682  6.047023246  4.142599115  17.42781742  16.82165005  225.6796073  21.68315654  3.542410054  3.618061797  1544.076213  12.41659556  3.124286485  3.30615621  4.104363534  6.41312024  79.35407904  3.141528154  14.47385063  6.909576072  6.008466692  3.292227918  4.797615735  1.327969205  12.01530503  5.99809006  7.434943735  4.3084433  29.89365148  4.977085867  18.84145508  3.099729359  2.773480425  5.643817009  5.256151249  42.02844467  35.37574559  43.33881768  224.9787418  11.26559744  7.945478852  8.943128632  5.098010058  6.662022884  23.0076346  13.4775156  3.094379483  18.89863992  10.99139755  21.1792804  5.778893912  40.57873582  7.423749456  6.863522754  3.051568387  8.988488206  8.455716468  5.122483118  39.51280041  8.434491421  2.474858302  11.72710173  3.784019197  22.14060024  5.02203072  33.31638326  9.26847588  2.460197065  3.06731087  3.594528692  9.174132027  7.813534076  72.18310195  3.048971096  28.18108652  9.136611911  2.452520376  5.809753061  4.141009073  13.39494786  9.727258868  14.64997356  22.78107895  1.531681245  23.95836657  3.759005796  10.06614621  6.698892976  6.21847927  4.504717861  3.519118978  45.22202461  6.240828788  11.95388858  4.987029026  2.8113366  74.30028943  3.481840335  13.1392906  13.97090407  37.22561077  10.10713536  4.755585808  3.010808711  9.369742858  394.4021219  1619.356412  10.79689643  3.957978677  1.676356868  13.38091701  5.598085677  3.531651005  19.78348201  7.460658667  2.119129484  3.686611677  16.46939169  1.608752247  7.602106602  2.962166841  2.932649073  31.38265849  2.974452707  14.49809836  9.42574787  2.657711105  4.424701605  5.70038303  6.708894012  2.108511463  2.95579412  5.591513149  35.22310073  1.89761031  3.961874753  1.417673556  1.673099842  5.772415415  12.2329557  131.8324791  7.901809823  12.44455528  2.068612122  7.55056732  3.535629237  2.393009134  5.123615868  10.01851115  20.9362135  3.839545884  10.5880581  1.209229288  2.225293671  3.700429189  55.25103734  4.837862158  44.6345005  8.634560301  16.48894049  7.411513702  6.179098836  8.254331711  5.587742086  6.371269318  6.746206881  2.859422435  20.30276691  2.869216065  4.118143329  69.70199688  2.798989109  16.46350577  7.055177435  4.171739726  9.333380112  3.303809128  3.457697715  16.0960436  2444.347136  3.80159293  5.0210565  41.51369093  45.86219634  2.472736137  28.75941795  4.987686511  5.463649458  3.450562742  28.36046751  15.09693241  7.757961664  2.638040836  22.83903798  7.093987921  7.682552063  2.322284241  33.07960795  1.483544716  2.826900007  50.39904079  5.253320363  22.81287846  191.4956421  47.69786682  7.75559497  22.0961067  39.02119161  26.71917195  2.812187089  10.03996131  3.623007389  11.30340998  8.217618907  1.117470186  4.683362687  113.6499273  7.278677501  2.81025534  7.534828451  10.97961081  3.357881456  3.856612406  2.830170733  25.22467864  17.26146458  19.00053131  38.95875235  16.11287995  9.501620597  1.879047786  15.1541621  5.398416046  14.69870428  270.0559745  16.94445941  11.60103627  2.615430319  4.417674892  11.83522404  13.92034484  3.25679802  3.613638665  119.3850673  37.61784631  17.59894495  2.900693306  4.869115738  2.361799842  13.13572263  2.886824611  1.413433392  3.303434193  7.134616459  5.594945967  2.786231879  7.229734666  2.7571813  7.060846975  22.1015366  3.300799975  12.05291843  14.10043406  1.772328687  8.49152217  10.93654727  3.613398881  2.727275283  74.05593852  2.0060093  4.409348909  2.703121179  2.801741795  2.771345237  3.391120161  2.736374501  119.3499104  21.91750505  4.384799275  8.055901333  8.396673542  54.17392159  2.010496518  2.700437714  2.746382279  2.419391306  7.843810614  7.181005569  1.649118755  1.534867686  12.02849444  2.752057152  68.55643207  3.301951753  45.91354026  4.766107598  8.585202956  2.746516919  11.78316585  4.148319109  2.747198908  1.787964346  7.198075505  22.79739536  4.176095613  14.8773358  1.551175706  2.713945786  3.257523683  1.173090782  5.313584795  7.07737386  8.053857382  314.6575261  13.06124504  2.767166647  2.661878812  108.7786171  9.772037284  16.40899162  6.019414436  5.484883621  21.01221529  1.54060142  3.917807028  2.721740994  16.75399625  94.32606001  1.693970837  1.576631921  4.350725525  2.674906162  6.174062801  79.87496387  2.658927538  2.698478909  7.946670148  10.79516395  17.600171  3.064580676  91.96197174  2.697604537  9.060439816  2.050023936  1.817223829  21.50713661  3.695831605  12.98441598  15.06286336  2.68393289  2.050464727  27.51315805  4.043146579  12.58018315  2.628277448  2.670324939  10.78637075  2.679186019  3.560704979  16.93803644  5.820509016  66.33424138  6.712411511  1.093026632  1.594018588  475.2190389  1.450115813  3.556023435  6.09075628  2.664898981  14.16133247  2.673780136  2.201983985  2.116947832  6.779008541  3.811856315  2.821834629  14.86517952  2.631686503  15.62574228  1.088936053  1.055504101  2.658516894  2.61192101  15.16210807  73.15849595  5575.040392  2.601437992  1.598863984  21.73583309  8.422350965  12.01293722  1.347589427  2.719064165  2.642198755  31.01296091  5.077341374  2.632917165  4.711358124  46.20640184  2.66332172  3.429897165  127.1974852  3.946564304  8.137882945  2.081673377  14.91675141  312.8512557  9.387742945  1.855069302  150.7322271  10.26567342  15.8778213  3.443413092  2.618522041  6.657491833  3.101493899  3.203893528  2.569610944  1.898607068  4.39117259  9.335018406  2.55240103  13.15776296  13.38489607  5.732285396  35.73575184  2.292871561  2.571421237  8.06917555  2.569801871  9.761524256  15.01335428  2.598931786  6.702782605  11.27523187  21.4025135  13.93291572  44.72237214  9.561009927  36.21136659  4.104034467  13.06251957  21.83021239  90.81197261  317.8392873  1.257602692  15.83768976  16.12527905  1.139309872  8.348168746  8.373106398  179.8270306  3.878757589  6.726829796  3.442731711  17.90906435  3.717399388  7.198686615  6.491341227  5.816444878  2.643629964  2.5753164  12.43278477  28.91796403  58.01552911  78.39973739  16.34105517  2.545352715  2.921342958  3.080914149  11.02669399  41.89984382  3.458729728  1.138552901  5.663361027  13.65315915  41.93199448  8.328182  14.73457737  3.454434437  2.926692955  4.110106117  51.86097017  7.981689692  38.82583142  119.2803822  1.161724035  6.416964486  5.506853313  6.415583258  2.545951514  7.450843934  4.747278604  2.542886314  6.44260181  18.6065074  12.80371043  6.279544132  2.602103682  36.27881437  4.136109851  11.938423  2.182239373  243.2793254  4.698370529  6.339824288  1.551462559  1.286369269  3.232600174  5.671250004  9.969722011  8.903228971  18.14422411  4.210674677  6.137323912  6.235258711  3.245656695  13.88085892  19.54397415  32.98065823  4.446340089  29.84857404  4.149336498  2.692837907  55.95113936  11.52686528  2.808929489  2.505533167  9.307665198  2.503243699  16.51949419  33.76900874  8.93536799  3.576167201  1.164085  41.97097554  8.183306354  6.285251819  6.406111369  2.982276991  3.404787192  23.25857708  2.072619173  12.87279264  23.52647622  10.91825425  6.391483546  4.719970729  18.23647289  5.383122019  8.393389088  2.490524228  3.718256547  6.301020896  13.03318796  38.85339416  3.354523441  196.6675017  1.064646014  3.684122956  4.889353749  2.471970094  16.23749713  8.208116302  30.64449335  4.051972197  5.531260033  5.516927301  14.39763743  11.76973293  8.729565338  31.50536949  12.37656631  9.02008202  14.13077125  16.36980047  6.440037153  11.5714997  8.195210865  9.660181362  2.166448701  11.25602029  9.733793225  2.88178254  7.216472305  2.482976293  4.445237368  2.443807388  9.698752501  6.306982267  7.548559495  3.280807477  4.600305684  8.088746868  7.711881578  104.659489  1.472603872  25.12742887  16.92999346  2.790662898  6.107700134  7.954417484  12.18689513  2.42962394  197.3369128  2.423897646  11.0878891  12.14353407  325.1309866  3.190184212  1.095657932  1.027079528  1.889765231  2.422482891  5.06807939  2.421806007  7.17859406  10.17526667  17.79024824  26.0013646  6.568669722  2.367495176  21.92867031  3.935871733  7.101308309  4.969940998  2.909613874  14.88881033  20.00880474  2.400477107  6.195439866  2.36929872  16.33677595  34.03903047  6.184380901  2.922072358  8.733192261  4.754200892  5.674795001  9.254289624  7.406586821  12.49713049  25.79578053  2.54391102  18.47291835  14.72319149  2.364719701  2.127391031  4.536276688  6.187630722  6.184025251  31.2562228  5.935153388  11.36676202  2.391143485  2.179149569  10.18254136  11.20813542  28.95747711  189.201188  244.8498736  151.8974839  16.05252904  106.3893317  2.33324326  14.93751994  3.400237508  6.971898226  4.288734211  3.898624787  2.374616363  68.2595865  2.402234209  12.3424301  2.02719402  2.152833179  5.483462935  7.518734984  7.981322436  7.152717346  27.08530713  1.026887549  14.69893671  2.718407466  13.85782992  5.462904826  20.64013363  5.684383618  13.31791389  624.6986522  2.342605785  4.162161524  11.12517937  17.67799478  6.820779547  5.50664266  10.05135062  7.804518696  2.318377177  27.25064519  8.199362244  10.24209866  15.52485835  54.89200115  3.934299857  15.82075797  15.5028379  22.13492766  6.03121291  3.98401979  27.20061742  2.984466063  1.327046916  2.341755188  2.708588316  7.459344834  2.324671014  2.32342268  2.312915137  42.56554859  11.61113133  4.501749826  1.723690603  34.55165179  13.45060293  13.25308698  3.730117926  9.060460862  2.719735921  3.552738601  2.281792675  10.81911014  2.865609829  22.21442998  8.318623248  4.44652888  20.87412147  3.722496487  29.56894283  2.308209909  22.63732578  8.787718434  4.379565929  5.937489695  32.68652581  8.905534692  7.024045896  21.69799802  3.791442853  282.6725021  2.289766794  3.592110371  54.77239557  2.246449848  24.08400204  8.393267439  5.792677889  15.19423232  2.86893727  3.10729357  139.1873822  6.293894939  2.607934855  4.4726118  5.146729719  1.426516473  3.041560232  4.180412929  161.0865795  18.78703482  9.066509483  12.45873669  6.204534114  16.19383856  3.023937084  97.67878434  25.43095757  17.92015481  11.1680405  4.324145732  13.27417743  1.055897564  26.08227536  18.83598618  12.19822412  18.29610186  9.169845747  25.00106858  9.30884905  2.264924362  12.34885623  10.60340117  5.940445722  3.054493255  7.539766779  4.480968478  3.121711173  7.228263007  4.452131669  2.508526813  22.32866394  2.247681431  5.724115724  3.179496905  160.6497802  50.61728743  1.450391851  84.43937632  10.76951361  2.484973902  11.28664754  19.2093831  6624.965504  207.3361703  3.047287646  5.73140456  9.087426367  6.119730245  16.10382494  293.1063753  132.2145933  18.25967371  5.894917342  5.755308978  64.14158655  500.4849098  3.036119123  4.326607879  5.018788028  23.38920143  1.724325996  5.72110865  19.24933987  3.700701813  1.759220042  10.85562474  41.45142671  4.385914545  3.815313068  11.84248045  2.207576138  9.240156666  6.607587582  5.843303235  42.72896023  6.43338129  28.43780271  2.222951475  2.213367489  223.6301642  11.5592227  36.42609008  3.485346575  39.24591231  1571.404803  23.19317816  2.291940744  2.005892951  2.702697033  9.469437446  1.484034103  16.70642344  2.20159411  2.999173849  5.520491633  11.18080713  1.112147782  1.602396374  6.697475924  1.22667206  19.54139995  2.700359466  6.427441297  11.44205736  3.039180481  2.152376879  190.3858441  5.170205625  28.10071549  2.193258189  40.64866651  769.5135676  7.254544698  2.165781381  4.941802188  2.289549921  12.4096349  19.18751085  3.46744768  41.75303611  10.42276908  15.87641514  1.494155823  29.30817023  493.1649511  4.422061305  2.295314451  26.27514431 | 4.381494781  0.686951073  4.875039856  6.375406192  2.93721698  4.989931593  8.048692057  22.49059177  2.864111436  2.253325296  2.017296434  1.613304156  0.98695354  3.800541394  3.701890991  4.418809345  20.68881581  3.084386718  1.647705002  5.587363193  2.299100118  2.506176501  1.425534098  1.462821089  19.74176317  2.558700426  4.679864276  1.586274049  17.86510966  7.752572272  5.586049997  0.493816731  13.66459227  20.60799894  27.86770691  7.301960193  4.023124194  8.767503658  21.00439837  1.112866974  1.574140519  13.32614382  1.158542158  1.722456094  4.422734141  3.18372262  5.325281741  8.397508883  3.904869078  3.024315089  3.068894743  1.352254802  10.37014347  0.66889455  0.976708788  2.307580005  1.151143604  1.088777081  19.52096227  12.19800385  3.02691948  4.018907814  4.144047914  1.639116538  2.588482067  19.93844993  5.276337568  13.24689302  1.014784935  2.379800486  1.910215122  2.288728515  1.412119753  1.954324011  8.67235425  0.957734206  1.066948103  0.741574744  2.783888798  1.869926326  3.496492896  3.147092754  1.388817299  2.047725796  3.938541227  1.091521215  3.569311696  2.64622978  1.690595401  1.731692302  9.650691157  0.585847952  9.020984858  7.60854382  8.464737923  44.5290445  1.686529243  12.80206966  2.646305974  7.153296099  2.506025046  5.763852277  3.729229348  6.179489116  0.725377466  3.005610046  1.559121846  4.072895244  0.818856466  1.331724698  15.95594171  60.21058252  4.188949347  1.706533217  4.335721392  19.70373803  3.24583273  8.820722902  8.777713872  1.771467392  4.396181126  5.612113915  2.77221051  7.514570494  1.907415323  13.65493448  5.76810064  3.274945908  0.618423912  22.80379048  3.280084183  1.49016087  23.64468492  2.561732833  8.340860345  0.707892317  4.864984842  2.382892173  12.75710477  1.962660155  3.736998562  8.532992468  8.096377741  1.339049283  0.855784927  0.880287384  2.437389239  1.533072049  2.801035757  2.813049731  0.65481881  6.560738341  1.614683125  47.53575241  5.232932288  1.142047566  6.992194647  4.030480919  0.884066861  1.097230695  2.76067167  1.409399065  1.522691088  13.51980699  5.302629732  33.9146344  1.358507902  1.773448368  1.614390502  5.24090769  0.87791176  3.918722393  5.773350263  1.484290904  3.863635065  6.354922466  4.548782235  1.151253686  14.20441772  2.96730045  1.404736012  29.39152819  19.14553539  1.866710995  2.723617318  6.01440534  0.612255264  19.39826787  2.35903438  3.851841749  0.861377304  4.301195002  2.671245995  1.443470212  5.027007307  3.329573369  3.343919746  0.99024257  8.460716249  2.423027756  14.76482696  16.33914059  3.913288953  12.04741089  4.071037042  2.909109048  2.334305234  10.4305398  3.125093957  1.798208675  5.103915475  2.183990991  3.907289523  2.01448652  25.01754205  3.55356304  8.90972666  2.242475857  6.674501243  1.070827553  0.879812413  1.494066078  0.971395198  0.680312146  10.55833675  6.797327282  2.584675053  12.02341464  12.85055567  1.127040754  0.63433818  3.381490134  0.623479729  9.051322042  0.70703078  1.261946036  1.680210694  4.446586423  0.649439776  2.688023874  12.84683104  4.048912562  0.482328345  30.73505416  1.969403312  8.645892895  3.935188522  1.692668185  5.185250363  9.176233027  4.225945143  0.868185753  1.29717423  1.624861328  3.025726554  6.986349699  2.234144218  1.046263562  9.778106312  58.63619078  7.765897191  2.239062882  2.46828644  5.586420467  5.840133002  2.043938083  19.85530847  1.909541167  6.506488351  1.725513046  5.133093949  49.70249694  5.760993922  1.897338821  3.200532377  4.06245691  5.345105165  13.40361051  7.578867602  0.788965116  3.850738713  19.16880531  2.026036625  24.76306267  1.015449505  1.406960379  11.0595772  1.095144207  3.846779696  0.690264523  2.775775966  8.074000278  22.25510252  5.318327625  1.418005802  1.947667242  9.025776468  8.92469511  8.927166903  2.165835456  15.83145801  1.545661339  31.1918257  25.01802964  2.473477926  91.95768205  0.7923525  1.958918956  7.172366221  1.59414607  3.865300771  0.73861705  2.132660149  4.930405055  2.630154843  4.160539084  7.838529605  5.561145472  1.831860163  7.092701076  1.591173101  20.81564669  2.482362873  6.634954066  1.885558161  7.226193348  7.923207853  1.912404677  9.118587009  0.823711907  9.881936881  0.778828344  1.835415346  1.636757127  1.333628391  1.160133625  5.477179676  11.91005867  9.324211956  0.960851686  2.165630545  14.78863535  31.91250586  5.948934273  5.734694388  1.449400412  3.558572534  2.512716724  5.962000032  3.777081463  4.505178645  1.386333296  4.996465224  5.091697214  3.785240274  12.53245487  0.568090216  4.986719374  119.9756068  0.740426949  3.132010202  65.23834567  1.344976769  1.335126645  2.743374562  16.12030661  3.136882116  16.79006876  1.407141767  0.824225923  1.805434518  3.512909451  10.91648312  3.212795467  23.07087652  1.710971558  1.018031847  8.789245881  3.113525093  0.757911386  3.000806107  2.1902091  4.721994323  1.051148111  18.98711894  2.87486439  3.562778091  3.615347567  12.94507671  1.596740461  16.56409162  24.29470218  5.207101328  1.848127632  1.738734785  9.897218239  4.245643317  3.066592767  1.725525675  1.102655314  17.09817485  0.851850753  3.478189178  1.673374472  9.79730975  23.17942525  5.863174436  1.597945524  2.219652107  3.707109879  3.186257017  1.016712937  17.55453135  10.68872652  3.254711912  43.64999564  6.800427995  2.426559575  2.081637822  3.806079284  1.601412632  8.540555101  2.66826922  14.29947323  2.656571789  7.563917024  1.979088067  1.156327443  1.060132819  19.75593152  2.792613803  3.683863667  9.564627895  18.44592681  2.29505523  3.679535752  29.85879329  2.875383026  10.35922283  27.7084003  15.03655603  4.215136414  0.751511599  22.91011523  5.860960761  7.647209299  1.011563407  52.51993881  2.135659663  0.993682045  1.430798128  12.31764854  4.345860389  3.610872838  7.322749709  1.673173809  8.699108717  0.981042209  11.70913836  89.5588763  3.276685625  1.628016843  1.880639142  1.519917837  6.415994875  7.633546428  9.860324761  7.608897221  115.0800863  6.364447398  9.871502266  16.2305393  5.847593168  18.9222808  1.027786307  5.432165936  2.601885986  1.05798876  5.391542196  59.08255992  4.407589757  3.641432634  1.326152145  1.205977219  16.95918609  11.2943862  5.153268007  3.799724435  97.74411491  8.084550355  2.171069152  1.155825779  2.219664218  16.24796074  1.598451395  2.990133805  5.365901888  1.748422965  8.102730163  1.681428496  4.287191284  1.11666826  12.67167409  11.81167682  3.909789446  5.047218046  2.772141893  1.816878616  13.65134367  3.146672608  5.684009828  27.94585066  2.093416786  2.362313574  5.910239473  7.12192934  2.813474464  8.352351735  4.180593025  12.03926427  2.784170735  19.7894259  1.710768161  3.459457821  30.47388372  29.38430149  43.84122479  7.115173647  8.520430673  3.730463801  8.887338323  18.89268819  1.847879938  5.453115885  19.03750797  66.9170746  1.318645928  1.998450649  0.979806987  1.650353419  13.36179152  3.069199403  10.35919795  288.8844532  6.391567301  4.479704554  4.315948675  17.09997947  6.223949282  4.168647936  6.375135939  13.03891209  106.5212499  57.47748886  17.13527059  1.242732542  1.032608896  5.900605797  0.876456364  1.340068397  0.748295785  4.274948889  60.91928387  1.727649254  4.654122503  2.223329652  3.64271558  0.923353403  3.730321108  9.01062036  5.133383393  1.478787068  1.460353391  4.488118241  71.7176505  2.817742335  3.362306623  1.723862847  7.919004041  1.451152288  1.430392705  0.725677249  4.559218436  9.962254775  6.231026903  2.403460454  2.767644186  60.44611328  5.75298482  2.691496433  16.86347073  1.181593697  19.60600876  17.07399034  19.50034212  0.872603855  3.005742186  2.164703342  3.165925092  7.399090937  9.653861265  3.885072175  9.172315368  2.306623798  2.408162646  14.7593357  4.545606432  5.986733333  3.187215916  1.095561115  2.611911492  29.11212308  3.366393074  5.231715487  12.02442166  1.471742297  12.45492439  17.52492468  13.6014407  37.61730885  11.70104423  8.712770309  1.174318313  1.820201607  4.355468778  6.969310643  1.537199671  5.645133446  5.203064718  52.92314078  14.42780157  10.56234387  16.53195796  17.48098652  8.976587088  6.709655827  3.698298199  0.801384013  7.749564701  22.0524731  4.924906046  1.464441192  2.830974919  3.864145243  9.999922108  9.318636482  20.47977068  3.531622314  38.57881425  3.00945546  20.55349443  78.2945057  14.42597418  22.63298324  1.563242615  20.92269217  2.572921353  88.72908524  35.78554398  1.313680775  1.234215157  108.5103835  9.301284143  15.96468861  11.63056639  13.78516037  16.13263646  3.261087503  74.55149424  1.579080104  2.878250367  19.69973354  12.26296862  9.351603632  4.90115503  7.42444496  3.462348115  79.77645066  1.225702589  11.94450285  5.792936072  11.27044418  2.031726197  1.320618997  0.932647105  10.80057642  1.677203838  4.947762021  27.6762068  6.296555049  79.05025863  4366.97722  7.780666241  18.88722015  54.63018836  10.62341211  5.863213324  11.17271135  5.579607714  18.42171891  7.923016535  4.879874851  4.298609035  4.547780319  5.002168625  10.77422822  82.3475378  0.954331033  1.589540966  10.55624688  5.006056769  8.779554363  2.683936489  4.114170506  23.74227367  1.205833815  6.784676792  13.91459632  74.14125011  4.618481076  3.035278114  3.167137905  4.418158126  5.230364798  25.78786474  12.22919671  3.144866599  2.694745483  4.560072201  2.398795331  13.90280492  2.514013685  131.0667691  4.503607162  27.35506364  7.033548978  1.615630569  3.043601051  1.22160877  19.71177797  5.773186829  29.57113368  1.721772718  1.829525756  2.457939138  2.518249165  5.214686677  616.0463059  82.85168753  11.87869366  1.653331404  10.07918626  1.29856605  1.989359417  17.42530686  1.085744686  4.45545082  9.558894549  1.759130775  82.11327218  1.111391796  22.21036827  2.47373099  1.232745681  54.29700435  2.773085978  5.985485228  11.77569879  4.627725916  24.97942625  12.89846044  8.734527326  5.20427133  3.437737861  22.01515556  6.35938465  8.639668198  4.833983758  15.11323258  0.798128927  51.31065707  25.41737565  139.284507  55.95493496  57.2201405  10.76920458  0.894468967  15.46162583  4.986426847  4.4138757  3.934782412  73.90125411  5.726150986  3.60724417  2.504855663  3.151584816  8.600540562  2.707196098  2.585977511  8.239307886  56.85589334  12.30818467  1.746773948  1.128958799  2.211700683  1.968790237  51.45149653  1.511345576  22.63865812  69.91247987  8.175192282  3.769468017  8.313261944  0.875793819  1.047085011  9.772489655  6.84853689  4.171237125  1.30926355  5.172519354  3.599588056  9.699544155  7.927036515  26.81267239  8.099141646  2.926653406  1.225493817  56.8676562  72.09874061  3.02172331  7.881903386  4.837436148  0.977521215  1.400102372  18.26701967  5.185692418  4.151734884  26.578519  34.42963837  5.623220413  3.275136901  4.327790609  26.24751122  1.326227173  1.177715771  3.816855225  15.88402956  7.530121547  1.007569735  10.86846304  2.647955584  1.954239226  18.29212092  15.75626829  5.16149464  2.004938511  13.00716303  2.334130571  1.678689862  26.11458572  6.069869838  7.44576854  1.055683572  7.085345265  64.40318207  10.88487909  15.36532874  7.512322822  16.04462173  1.634966975  93.1180914  5.087504011  0.8519387  3.217738318  12.961603  5.53303265  7.420591218  1.658535553  7.162761777  40.71079072  2.268432017  6.168937444  7.54502649  1.140729408  139.6817444  8.132656452  23.83499284  17.89236879  0.813024942  5.518537983  1.997903953  6.822294953  1.181655384  44.756242  10.72895351  0.825067516  9.812143612  1.358867829  7.038173887  18.30337687  9.727602311  147.056279  1.984526109  7.763152372  22.63353177  12.90695545  23.63749887  2.642446373  0.942102492  172.6901587  1.5122827  6.983969833  3.786181598  13.2682095  22.31753663  50.11239064  3.35339847  13.87797193  20.89506562  23.10416449  9.226580085  16.62949376  6.691441288  133.7398252  9.273009584  3.621067877  9.010209156  2.254165847  1.905591119  248.01819  2.420627692  6.44294638  43.46505612  6.625866022  5.205774524  3.444900801  31.54987926  2.090184696  14.12937154  11.79155325  37.32754537  6.837354706  4.427155803  2.842714292  6.490063856  2.564656424  20.27126616  3.018949489  6.411099623  5.204058696  30.5334761  1.213807018  0.964282191  6.406735376  5.985825254  2.666273767  6.615852614  4.906516295  5.397898111  3.541982155  17.42669092  6.948196572  21.94443834  23.32201149  1.041841872  1.972060319  3.3534273  789.1920898  6.131756635  4.581498216  9.737881822  1.197809111  8.647249205  1.588925151  0.782927724  6.09646024  7.498174526  37.22410484  27.00208647  16.53045214  1.261071177  12.54839435  57.29778714  31.40557081  38.44553415  8.209283244  6.108027178  10.70137372  69.29830995  8.311064789  1.197132912  5.877954381  8.228244087  2.1871827  3.16989931  9.118287324  201.2473618  54.73634996  7.826326051  6.290506428  200.4140216  13.01929423  5.909925505  7.958973751  5.800361681  10.48034732  62.93860773  16.56423714  3.956381092  1.598015762  29.54755621  2.064659711  7.119229667  1.515687968  8.02539581  1.881094535  16.58961762  5.755916036  5.773096176  10.63741383  20.05058634  6.045495909  65.76288322  23.81110538  5.579116321  5.147112602  41.92230662  1.562819557  5.182938138  3.542815612  1.476198628  0.91255761  2.946624976  17.60023312  1.091105166  1.278406611  4.432950297  7.65135177  4.089763287  4.310844922  10.75979596  2.330038123  5.248008834  12.73744402  7.527862375  1.542177453  5.524986453  19.44462774  4.277617999  400.7979292  7.4411921  2.896382321  5.45216116  5.480993177  15.65210204  11.73848237  1.959587863  23.11871305  44.19050425  7.506577687  18.79084363  15.76467858  5.427573581  11.34317693  9.893873231  7.991462992  30.77427503  51.94571224  10.00057517  1.950406517  1.06689462  40.09548247  3.756294146  7.209542995  2.065084818  22.0078132  27.81417633  1.232794929  2.198043693  5.540471498  107.475125  5.793369441  2.449447929  1.014719672  4.621880872  0.775810589  10.70626658  15.15478882  1.092006645  9.462277384  3.49642652  2.336613623  1.882590669  2.613290827  3.304527409  39.42012558  2.149007878  2.535219042  17.87880877  5.18856547  7.212045611  2.515067549  63.903722  9.961435503  14.90274677  17.19697569  3.298160581  9.335241992  12.45104355  5.000747916  5.11766631  3.221235288  2.05848432  6.597530199  7.675961062  15.91690549  37.06334012  1.41938424  0.938250678  2.456732555  5.000100443  13.52311405  4.368417648  2.773371803  39.59560171  39.1773288  4.961617863  2.679325675  4.911302528  14.22220794  9.078185312  1.169667851  8.431527813  4.676977029  2.266853559  12.47403148  16.59134492  2.499797729  14.96769826  4.187621812  1.550556922  31.55277868  2.554526378  4.516240094  15.62556236  2.639673964  12.62567678  5.337582207  6.509669569  6.788542855  5.280375863  52.40449845  4.811069182  5.43414537  39.44256262  52.12932698  4.870051554  438.1290074  21.51484098  3.631438578  21.00371395  25.25595434  23.40871525  17.04487974  8.365942436  1.481169289  1.426358808  19.59322285  11.86440413  15.64152632  1.321407785  11.96081844  9.340004926  27.87168995  4.822262942  7.07228539  5.949973875  4.807148172  59.4180399  2.49997127  11.90323603  1.732074035  7.266344417  2.818657259  1.710864937  754.9333849  2.219583951  50.35953874  2.513344389  7.778707198  8.951183588  1.734627155  21.78094353  2.02109036  19.61014196  0.966336674  0.853842484  2.558452588  4.446015304  2.222958435  6.175705406  1.819963243  34.87600107  2.218886535  3.74857739  56.39207279  6.885255891  8.143579085  39.6628036  11.49647287  4.666126863  7.206396077  25.90492991  7.94648439  1.280480353  4.925178967  1.373833768  5.638149284  4.518467316  9.445583757  5.058971934  71.31571592  7.822218561  7.622980991  1.726277368  61.91612203  4.570435647  4.298874246  7.01085976  26.41985409  0.908065372  1.721948375  0.787586758  11.1452853  12.81818946  1.002110538  6.406459089  4.534491968  4.662503212  4.824858089  2706.77767  7.345582516  0.998576011  7.636522116  16.5365095  4.532853392  7.192350543  7.266696406  6.565958415  74.58857156  10.17661653  4.491396982  1.482858985  4.389358588  2.640617415  2.420203927  10.64293133  2.680474875  228.6068112  5.556067544  54.22616727  4.500724487  5.624291048  8.96093279  1.583986959  7.733404376  1.520292524  2.202849  4.394378962  10.06622943  5.62376467  5.713776229  5.948243745  579.8401182  2.397620803  1.399687299  1.728012925  88.89973204  4.478574525  22.74880379  9.166857375  11.83821496  3.907778256  4.33449117  1.734295424  34.31099913  1.171205778  2.485381177  5.750715985  20.85229624  9.012118594  5.875973951  1.826267114  9.816967784  8.714757278  17.64583912  5.265904953  38.77458863  1.112075271  6.218953206  12.01374513  1.355409996  30.33222319  47.03426455  32.20929064  4.365235347  4.273265586  1.15815646  3.69044262  18.09739688  27.83502187  8.793940077  1.341534117  14.14854307  7.387976088  2.79934955  21.13610442  4.235161734  11.46278417  5.313889447  2.069279982  5.846420729  4.537368422  34.89497378  5.436394857  3.513053015  8.74779222  14.62168806  6.952007409  37.80865876  34.55815971  24.2401847  8.953752492  2.629911351  1.096257922  103.3020962  29.46806125  3.541231475  5.666917773  3.799768506  2.340534837  11.7469662  3.192718371  7.890003206  1.210853354  0.85921777  5.257665373  6.525817715  4.224789611  0.930533786  6.106736046  13.06772374  8.51343641  1.59847578  93.39360315  6.190212972  3.977001872  4.33209661  702.4719877  4.163463723  17.40254065  2.256668838  21.31542533  90.03230121  9.824705928  4.151330393  2.787667365  4.26299309  2.153924558  21.44508969  49.09120117  37.55667605  2.763910467  15.82818956  2.716908706  14.80270128  5.552139699  7.81695017  1.187887611  4.416528958  18.32290028  6.187062597  8.786452694  38.52425733  338.7701022  14.2691888  9.756427793  4.735842103  24.51550988  429.8343344  0.733041557  1.35830163  2.077616188  3.367640221  1.045782201  25.39675572  11.61830303  4.075474626  12.40528843  6.151447825  6.757805209  4.960475692  6.652339661  63.91920973  415.0847139  18.06751194  41.65850233  5.131243136  1.15718744  22.69119566  1.903122267  61.35371339  7.732855478  25.35459314  20.3970036  6.645428622  1.438934266  21.18730888  3.817868679  1.363876309  28.11169459  1366.930795  53.41294182  5.21366707  700.9542583  21.71579165  4.039451385  16.51102041  2.80486351  0.903394151  11.93762988  2.114805434  19.3025335  5.731378061  52.19914551  31.86977426  1.855211841  1.429247241  3.889050897  3.936795066  21.25366542  2.67465699  5.518116401  7.855913338  85.65006309  1.626748746  3.87407514  172.9264672  3.856088123  43.18794241  4.650487783  18.77721721  9.059697277  10.66711282  154.4612061  10.60725472  1.767690766  1.548679854  7.712176013  1.835002211  56.82569791  2.432390951  17.37358231  80.1315275  13.94739215  3.548240836  14.15700078  3.801690092  4.073451058  38.72183634  3.815744129  18.86450067  9.83276173  3.316845987  13.26708368  5.570456744  2.41096918  3.872732592  11.81961221  1.13980257  14.33764085  3.870350792  3.92988842  1.256935208  5.061874535  3.78023644  14.88216707  77.37819153  11.87152415  5.352724761  2.650805752  3.767401416  10.09101009  31.24021904  349.949835  3.816360587  14.62505832  19.2644412  16.91941074  36.00035497  74.31786196  427.6384559  7.487375885  1.35306566  3.734821818  16.14330121  87.95334082  1.155116552  10.13123781  9.841668215  0.978957902  3.772173577  16.08485936  4.931242194  9.704768945  3.783334667  12.70924288  1.406660647  11.39058242  0.987385252  26.32370616  2.851249796  20.31015072  3.776148229  5.341747643  12.74976561  1.250897839  4.858393929  1.123701185  8.626049448  3.513595048  4.925870605  2.601436919  2.839254024  1.067667971  4.608164187  1.067800687  16.46444118  26.49857765  2.191942657  1.723678881  7.977779168  11.52032237  7.5028626  12.35594126  2.748187703  24.25677271  13.33038818  317.295368  3.131260048  8.173434505  3.636220312  11.28346985  2.639193768  35.93692687  7.307669918  0.86162632  3.687842449  95.66808426  21.14778398  17.01521181  9.710122641  3.631095469  1.92875593  0.917007061  1.084558339  55.06089907  2.618055129  20.48489573  5.307183777  7.096808317  17.17480341  1.433414937  3.647575301  20.7624445  3.458469557  9.766387638  24.5349339  4.61245276  16.14945496  3.734235654  9.66961016  9.621287623  4.590342954  1.336760409  148.8064517  9.925971648  22.55410763  3.615782154  3.057410421  14.84803639  6.563729787  19.47789737  6.268790744  1.538273793  7.572211385  4.659086743  16.55869483  8.257884715  1.820982649  17.1738809  3.270534508  2.980848479  39.73341886  2.630431226  4.24463875  3.005571248  12.1791656  29.30714015  3.026257682  3.809665095  11.56729302  3.607692101  7.462467528  7.060015731  4.777833696  5.625429579  9.939813381  25.20072065  7.052613437  8.206431831  6.988193037  3.556297699  15.1201549  4.527997121  16.03767694  2.544106668  3.265231508  1.168799815  1.735197956  9.5163859  10.47436936  3.479314278  1914.596974  3.181757341  3.539406723  31.24690405  6.356668396  8.620991567  5.932579813  12.12851544  5.037846628  16.4296135  12.39954435  10.65111608  8.155809681  3.486324411  17.58903343  6.817670033  98.6220289  1.510623433  7.987306159  2.387677397  19.15295284  16.07181055  3.514539777  309.3742126  6.478667215  11.68882017  4.457802294  8.769026464  3.459005523  4.546884581  30.51131586  4.552815236  1.936190541  35.77081086  7.766725793  14.18486299  7.962002703  13.98759744  4.054334456  55.11812756  11.53050651  11.06716097  50.76100387  167.643904  92.97367154  69.04716349  8.061575067  4.794343089  34.24091786  124.5495962  30.30687588  1024.268083  1.772896057  7.842178964  10.10064106  1.211130412  5.680827748  4.365273098  23.94100644  1.434877165  7.81281309  8.829055013  64.18952044  6.637115374  4.151132313  8.823925504  3.408059489  8.073621924  4.89641321  4.871331466  5.374525324  5.802038071  3.658854182  96.12604615  3.41288043  7.79062882  287.8921914  2.236013083  2.039674636  3.401181075  9.142767522  38.34722316  31.01154905  3.307077302  1.026889393  34.10368567  7.011095658  4.356782414  3.721870119  0.922217787  7.229804877  18.88519975  9.927045569  43.2037459  8.565516985  20.01335568  4.222961041  3.103384417  2.520574033  1.28738436  47.78824614  7.307781068  6.642086144  4.342084317  3.016079375  1.138602581  11.33773785  41.11394201  2.161616296  4.029604865  4.005942503  2.17090068  13.44275198  90.45402202  137.0618229  12.80621039  15.95218602  11.37630781  21.42205286  10.04917159  22.07545133  1.656566241  6.832355244  3.264096256  7.150007332  97.13416425  14.38530371  56.93236742  100.1884645  152.3720565  4.344813533  2.786014924  1.383863375  2.769685453  6.889888439  3.294321282  3.317941162  17.81203445  53.30322767  1.292636023  57.61864951  18.00738685  1.337244819  46.06144753  3.274248209  16.66169847  26.91926704  6.489867971  89.49836551  7.989207697  3.423125425  16.4295478  5.822934127  90.63853928  127.8817176  13.02288918  7.215735353  2.292825782  9.84222043  12.582356  1.173415751  9.062157898  3.405535086  6.962805583  3.251071454  20.44098723  15.14801907  6.203644827  3.924517702  17.91267802  16.05334619  230.7634918  22.19095151  3.343828376  3.381920566  1478.856414  12.73883389  2.936607376  3.117826865  4.217768475  6.111988856  81.15708622  2.966515607  14.81887918  6.604925263  6.163947547  3.100617462  4.561196117  1.200279127  11.47217861  6.153263913  7.091746892  4.087446124  28.5758996  5.110078428  18.02219603  3.191437328  2.601016853  5.372715705  5.008897474  42.98831383  33.86558826  44.33110814  230.0289172  10.75491996  8.143020601  9.162601665  5.233150651  6.354479283  23.66586944  13.80065176  3.185929172  18.06821069  10.49907014  20.27266476  5.928023622  41.5015243  7.089852946  7.036095224  3.141851475  9.209226046  8.072813724  4.874209036  40.41025659  8.051338714  2.326035271  11.19671673  3.585269681  22.6547118  4.784131709  34.16672536  8.088006927  2.297733734  3.158059712  3.398473661  8.756250887  8.007172134  73.80982062  3.139177342  28.91677517  8.738255989  2.29044686  5.560180401  3.934220511  12.76853941  9.298926013  15.05309039  21.78896261  1.404230514  24.52446483  3.562480083  10.31162737  6.866594744  5.933672139  4.286225514  3.326191362  43.2614314  5.970385521  12.2418573  4.747400333  2.636874804  76.11361143  3.292630561  12.56683193  13.3560991  35.65595173  9.678938615  4.880672698  3.099885892  8.955066735  403.1441194  1506.653978  10.3376155  4.068037381  1.507878156  12.79912991  5.339448926  3.342408121  18.94447019  7.02560051  1.967801942  3.49426567  15.75800998  1.467084927  7.263430222  3.049804913  3.019413838  30.08751398  3.062454266  14.87433463  9.66708456  2.500379542  4.210691418  5.43675868  6.876891668  1.958022477  3.043243651  5.332082629  36.01977688  1.755397306  3.761440792  1.268830896  1.540569307  5.504108538  11.73330384  134.7608788  7.00196889  11.90883506  1.927644039  7.736425297  3.345665798  2.240147086  5.267617153  9.576696531  21.41148114  3.945924673  10.13141349  1.09720425  1.911899736  3.508491985  56.48247144  4.973952918  45.63357257  8.151529565  15.78759463  7.593257666  5.901043483  7.906860115  5.730775467  6.529454064  6.445968677  2.944020732  20.76794037  2.954104114  3.914850492  71.29337742  2.632539908  16.84583514  6.741853383  3.968867938  9.55735779  3.122752405  3.273132134  15.40832399  2352.026899  3.907024837  5.169608468  39.76277411  47.01659808  2.319757168  27.58576757  4.765001284  5.218795537  3.281922994  28.99662949  14.45255875  7.956996824  2.392247092  23.35721779  6.554568245  7.894967085  2.170796053  31.73073299  1.360281478  2.910536102  48.41525098  5.015590723  23.33126899  195.6952629  45.77464944  7.42939264  21.2066826  37.46252882  27.3230879  2.895387891  9.612116563  3.43992774  11.57006165  8.415627154  1.008431009  4.467907577  109.1707623  7.4564914  2.893398989  7.735732901  11.27396104  3.190434539  3.963496122  2.913903594  25.79131331  16.54143671  18.22294327  39.82253855  16.48276365  9.094411289  1.744200947  15.50425102  5.550106565  14.08214109  259.6650084  17.33255909  11.1030768  2.45763688  4.548375333  11.33347414  13.33710297  3.063253462  3.712525119  122.0075158  36.13980395  17.99926806  2.745911806  4.649447562  2.214713543  12.59110608  2.722430368  1.296742704  3.132126262  7.308168197  5.352889107  2.868664775  6.920932509  2.838754711  7.232216184  22.59851378  3.126369091  12.33481821  14.42818619  1.642464972  8.139852527  9.958881688  3.441007113  2.807963901  75.67687795  1.867117927  4.210085997  2.783095178  2.884633564  2.621306529  3.218046426  2.817332327  121.9501216  22.46761567  4.181340169  8.249047845  8.600171017  52.02800786  1.872068274  2.485728625  2.827636192  2.271760784  7.455083365  7.355488816  1.523904653  1.414360733  12.31011257  2.833478961  70.12238517  3.399642634  46.91170591  4.900075441  8.227377467  2.827774816  11.29542525  3.963511163  2.828476982  1.664544647  7.373504729  23.30837952  3.99898658  14.27726864  1.430813064  2.79424004  3.353900123  1.063110898  5.077286153  7.249232029  7.718145531  321.5487536  12.52828197  2.849035483  2.740632623  111.11722  9.365063519  16.78194717  6.167997963  5.099946463  20.17093737  1.420014907  3.72838402  2.802265875  15.64559336  90.66375671  1.575262289  1.45674366  3.895945289  2.524507649  5.910354366  76.87114085  2.737594033  2.778315564  7.613521578  10.37662774  16.89532449  3.155248744  93.95057847  2.777415322  8.693688066  1.914734492  1.68954867  21.98565389  3.39414532  13.32320054  14.45813986  2.763339188  1.914100874  26.44870529  3.853587973  12.8700975  2.706037136  2.749328635  11.03809314  2.758451877  3.659010024  16.2735154  5.962223092  67.91460393  6.874015704  0.988879904  1.478674418  485.2005501  1.333308391  3.654189973  5.840548076  2.743742146  14.52110771  2.752886057  2.063241661  1.943694548  6.942039334  3.629284137  2.666048811  14.26380973  2.70954705  16.0276826  0.986269914  0.896013302  2.73717124  2.473573428  14.58055443  74.7133557  5382.239384  2.678403614  1.4786488  22.21702151  8.082446991  11.52478574  1.235551  2.565788024  2.720370316  31.7642311  4.856047168  2.710814123  4.504389474  44.46106992  2.497341118  3.261688223  129.8928545  4.056109318  8.333899879  1.947320308  15.25269789  320.3186253  9.606287734  1.728274452  153.9354756  10.50188779  15.03717245  3.538247962  2.695993107  6.816927457  3.193254074  3.298683278  2.645634936  1.774173969  3.920336259  9.551018622  2.627915853  13.45593084  13.69175322  5.872010224  36.5090057  2.156524131  2.647498788  8.27803914  2.645831513  9.377381956  14.42679891  2.675823259  6.429132191  11.53339168  21.87722756  13.37088296  46.01502892  9.178561844  37.07702565  4.209767498  13.35831056  21.05596404  92.73098564  325.6111957  1.152444484  15.20794381  15.48615264  1.035911553  8.542557209  8.042936831  183.635993  3.704310363  6.888316839  3.537002696  17.22257923  3.541134225  6.90797973  6.645861153  5.572140006  2.721843868  2.651509193  11.94957218  29.54057459  55.87834007  75.47936515  16.70376928  2.620659008  2.765554951  2.919578973  11.27848528  42.79376291  3.553474027  1.035132186  5.823698844  13.14004937  40.42605633  8.522787828  15.1406422  3.284740705  2.772301085  4.231706889  53.16963277  7.670046248  37.40102904  114.8766375  1.059506999  6.569283917  5.276620666  6.567861824  2.621275523  7.15422274  4.86500127  2.618119637  6.595679742  17.9123216  13.09337274  6.450527235  2.460195422  37.05182217  4.243677739  11.47298689  2.048524539  248.387083  4.499808447  6.082449645  1.438491816  1.179291907  3.075693358  5.809169051  10.19764695  9.107185848  17.46067423  4.024921679  5.893375569  5.981982071  3.085458447  13.33529741  18.57456244  31.77380531  4.577888612  30.48624052  4.272097932  2.544474684  57.1461556  11.07303627  2.656489157  2.579661368  8.950519633  2.577304163  15.89355359  34.48949483  9.13929105  3.410794742  1.062036609  40.57082867  7.878252707  6.433674425  5.959095238  2.828544944  3.241634772  22.45433877  1.944307987  13.25364449  24.03289852  10.49008621  6.134921553  4.836709483  17.5520441  5.163506931  8.597141363  2.564208377  3.549311179  6.449910043  12.527631  37.43466928  3.446552453  200.7770392  0.967064638  3.513473591  4.580418927  2.545105304  15.65038252  8.428054629  31.29411467  4.157050805  5.323280457  5.650203396  14.71911139  11.30658857  8.936222399  30.35583291  12.65162187  9.226764591  13.13188919  16.77850718  6.18642031  11.83110481  7.878455744  9.877963622  2.039675683  10.61153491  9.955358944  2.732585074  7.385404557  2.55643713  4.260761277  2.516109382  9.918296837  6.456047786  7.25786246  3.370655543  4.713680039  8.275725091  7.414352307  106.821284  1.368370982  25.6613425  17.31096605  2.646975485  6.250869732  8.137344272  11.71247957  2.501506305  201.7946461  2.495610594  10.66645018  12.45823738  332.4711111  3.034324155  1.000170496  0.883637884  1.772390559  2.494153982  5.202852603  2.493457073  6.90136345  9.789164148  18.18170774  26.55204652  6.725477474  2.105382524  22.40050857  4.037250453  7.265848666  4.769642035  2.762036725  14.33434185  20.43426864  2.267872324  6.341205314  2.439396315  16.69180405  34.75486425  6.32981916  2.773968104  8.930766052  4.564114676  5.461327721  8.901842733  7.120964981  12.02780828  24.87580781  2.406384852  17.80124465  15.04582637  2.434681823  1.917449898  4.647756694  6.333165131  6.329452988  31.91168304  5.704597279  11.62051137  2.461887375  2.056772192  10.41426346  11.4570889  29.68859178  193.595708  250.8384658  155.031657  16.43822525  102.6741672  2.402274126  14.39213697  3.242308954  6.615812448  4.113548175  3.998901527  2.444871285  65.89131206  2.473306227  11.88674207  1.904404165  2.027806654  5.268153162  7.689568662  7.684796742  6.879090968  27.65468573  0.941563711  15.03628891  2.530872574  13.34797172  5.243775639  21.07819772  5.465467892  13.61331218  638.3588081  2.411913648  3.990578305  10.46226273  18.05837221  6.977461186  5.290061111  10.27330092  7.513985422  2.386968217  27.82234483  7.896612743  9.862315679  14.96619177  56.02141508  3.770927796  16.20832889  14.92632687  21.35768298  6.172119572  3.821608462  27.77259888  2.837321897  1.232512929  2.411037886  2.5673646  7.191229149  2.393448263  2.392162996  2.381344579  43.47986518  10.66186519  4.613094208  1.36169541  33.35457703  12.95979415  12.77385648  3.569086866  9.262825218  2.580888558  3.391471967  2.349301334  11.05655399  2.72305233  21.50048787  8.505537276  4.556239506  21.30965273  3.561261544  30.18121848  2.376500143  23.11172742  8.98825784  4.486233535  5.714815285  33.35832478  9.100990128  6.763113447  22.15104702  3.889357219  288.4014368  2.357511374  3.434500057  55.89777638  2.312912861  24.58776476  8.100743288  5.926527302  14.6434355  2.953817071  3.191199401  142.023346  6.055076922  2.685092691  4.294873384  5.269130414  1.326521882  3.124505964  4.012141783  164.3569671  19.30559763  8.741032057  12.72959743  5.898720273  16.53705277  2.878328438  100.168558  24.54967182  17.28730053  11.43915403  4.429614637  12.79847386  0.96876455  25.17630946  19.31025196  11.75472152  18.68202062  8.847661072  25.52285404  8.983753356  2.331933958  12.61693261  10.24470815  6.078666964  3.137645634  7.710135247  4.315813723  2.98005712  7.397000371  4.275315733  2.379294753  22.7936804  2.314180882  5.855936671  3.033495907  163.8994872  51.65886144  1.349850012  86.14624957  10.39001825  2.55849384  10.88054261  19.61510476  6421.657742  211.5045207  3.130226842  5.863441153  9.310727614  5.893007794  15.55460182  283.4260855  135.313554  18.64443752  6.06932318  5.543579214  65.43616898  484.7824129  3.118282958  4.432770547  4.787948897  23.87383753  1.61737704  5.85284063  18.64739069  3.550970641  1.658574983  10.47471277  40.06840514  4.211667704  3.656280452  11.42788222  2.272889042  8.913396391  6.765546749  5.97865044  43.59590832  6.578160493  29.02508355  2.28871927  2.278851734  228.1129915  11.15949156  35.21961356  3.588463338  40.04350918  1602.387593  23.67164701  2.171838516  1.891762257  2.583266965  9.130784947  1.382882004  17.05625881  2.26673003  3.080865547  5.314873066  10.78415833  1.024328087  1.499057276  6.466762173  1.13709554  19.99261742  2.567021  6.57248571  10.84151683  2.913473712  2.216056669  194.1899755  4.882347498  28.66920049  2.258147485  41.47305529  784.7135391  7.469176198  2.229857753  5.057519021  2.357288085  11.9696302  18.55769768  3.562008958  42.59242728  10.6490306  15.28466576  1.394111873  29.90686589  504.4367608  4.250489487  2.363223163  26.80958931 | -5.324332183  -5.527895189  -3.852758773  -3.743637869  -4.767634516  -4.215313078  -3.722900037  -3.655861871  -3.841665476  -4.583752225  -4.104720941  -4.899500012  -5.555010917  -3.600552203  -3.923621336  -4.388972211  -3.7383876  -3.644440481  -4.187559469  -3.688692036  -4.113160755  -3.775845262  -5.348563472  -4.97659955  -3.358553918  -3.76133788  -3.447210375  -4.47911455  -3.897803853  -4.406454528  -4.109687038  -5.545129704  -4.20411217  -3.310635632  -3.810564194  -3.3450481  -3.423319005  -4.043838333  -3.332004994  -6.106251553  -4.189643003  -3.955527114  -5.168961743  -4.199143983  -3.876447604  -4.093072954  -5.075613647  -3.305757967  -5.147924506  -3.497383767  -4.347087985  -4.373256912  -4.104857523  -4.885012074  -5.548935192  -4.663712189  -4.7395023  -4.600114346  -3.493341354  -3.244580265  -4.307199888  -3.603518182  -3.821569328  -4.1205442  -3.64491846  -3.186294581  -3.99117525  -3.225375795  -4.377391207  -4.663865095  -4.435934852  -3.667538634  -4.192585135  -4.371757962  -4.341396042  -5.581813573  -4.829384175  -5.461843529  -4.574854992  -3.917716293  -4.854206661  -3.399229266  -4.705170131  -3.748886208  -4.209212056  -4.280257756  -4.334660859  -4.524183115  -3.870110177  -5.001308122  -3.164213124  -5.304850014  -5.090804061  -5.088402959  -3.880581458  -3.041277786  -4.317624075  -4.312168033  -5.670089556  -3.11795961  -4.602159972  -3.154177693  -3.957606508  -3.548781683  -4.653631111  -3.96398669  -4.60486832  -5.093318906  -5.318709877  -4.02464573  -3.059532294  -3.164772576  -3.176457219  -4.660296305  -3.527586342  -3.271901428  -4.266152267  -3.199346905  -3.532064041  -4.450881917  -3.12261581  -3.097362175  -3.398211482  -4.713438118  -4.499801703  -4.191470275  -3.092503594  -3.70837399  -4.800151324  -4.037170169  -3.274329713  -3.900782836  -3.664862435  -3.445515687  -3.057082512  -4.688046273  -3.902236995  -3.604648934  -4.494978387  -5.252412822  -3.164286344  -3.061700439  -3.224783362  -5.26089997  -5.197842564  -4.698071178  -3.964522958  -5.201861842  -3.357118321  -3.350726319  -4.803394094  -3.846713588  -4.388466815  -4.467646874  -3.973650382  -4.493729931  -2.994156431  -4.11054648  -5.467151854  -4.920361347  -4.015541634  -4.280180384  -3.837245305  -3.022271953  -4.662074034  -4.546781339  -5.06478707  -4.248412251  -3.722650788  -3.02544659  -4.329470279  -4.803232798  -3.657129896  -4.309636161  -3.763282901  -4.421956952  -3.036662139  -4.125792465  -2.971049882  -3.932840895  -3.879101935  -3.06598364  -2.913890157  -3.610316368  -3.488608649  -4.433227767  -4.848725223  -3.243529275  -4.068815268  -5.354233742  -5.055900681  -4.515653605  -3.87815923  -5.135680598  -3.916715004  -3.115207752  -3.08351156  -4.447186533  -2.949768639  -3.892549629  -2.92138768  -3.187079048  -3.599898454  -2.942920372  -3.018836926  -3.83670607  -3.406577742  -3.238282469  -3.447370377  -3.605559919  -2.956993159  -4.398013465  -4.073533337  -4.659436759  -2.850381536  -3.376999049  -4.854535698  -3.418314057  -3.421221938  -4.056809585  -4.237255846  -4.779912826  -4.544658755  -4.505513134  -2.901802747  -4.868230446  -3.267594147  -2.877132775  -2.891485924  -3.999693291  -4.79173532  -3.058569007  -4.567338927  -2.875255823  -4.679318922  -4.644289365  -4.26932011  -4.378393101  -4.524508633  -5.50034088  -4.334214013  -3.682309652  -6.087498881  -2.794375059  -4.063324845  -3.881239316  -2.953186275  -4.844077088  -2.874689155  -3.24394799  -2.882872756  -4.200106734  -3.808658063  -3.586936087  -3.404800557  -3.382636649  -3.716654536  -4.346909803  -2.837410059  -2.736576116  -5.188754999  -3.342490518  -3.249355927  -3.68654536  -2.846206738  -4.676742598  -2.795747337  -3.701070521  -2.83546304  -3.490893079  -2.840772842  -2.738767679  -3.270673687  -3.774098071  -3.726081424  -2.898116389  -2.845783349  -4.088247865  -2.83490617  -4.613464659  -2.906495445  -2.759754352  -3.348674321  -2.736534447  -4.349344275  -3.653378602  -2.786039064  -4.303293611  -3.572732119  -4.385821863  -3.219183731  -2.766420451  -2.730800035  -4.136029005  -4.148915255  -3.378671531  -2.780602426  -4.020326391  -2.782219976  -3.711101371  -3.128085536  -3.558069927  -2.997148705  -3.301119747  -3.382516902  -2.684900305  -4.179291629  -3.37682451  -2.762370886  -3.524700477  -3.834973621  -4.251569693  -3.272234889  -2.788526179  -3.118480555  -2.830043439  -2.713316511  -2.72336294  -3.370138542  -2.740706571  -3.482640136  -2.661992697  -3.135764933  -2.755934385  -3.319801649  -2.712233061  -2.720331615  -3.30958193  -2.699918991  -4.096003382  -2.707873375  -4.158659231  -3.369309576  -3.438477005  -3.621889782  -3.756915172  -2.705598466  -2.699062548  -2.682906114  -3.925443724  -3.605266206  -2.867859896  -2.890407089  -2.70114373  -2.63611208  -3.503147085  -2.936696789  -5.109377771  -2.688734639  -3.663450152  -2.733266182  -3.635774906  -2.689071738  -2.693911175  -3.506630182  -2.64481389  -5.257442333  -2.636584975  -2.582660893  -4.42509295  -2.897338938  -2.938257983  -3.565957675  -3.64987495  -2.971605174  -2.603203229  -2.843412089  -2.582870776  -3.509558006  -4.016240034  -3.396487457  -2.76551485  -2.633467457  -3.407161423  -2.567291004  -3.762969355  -3.803033817  -4.595801433  -2.828092424  -4.082028859  -2.862150063  -3.119706575  -4.264245931  -3.717826116  -2.557920313  -3.332872839  -2.961081432  -2.74423599  -2.840715132  -4.348404143  -2.536370234  -2.532638897  -3.697586595  -3.208283411  -3.265068448  -2.576375159  -2.884402421  -3.31762945  -3.2775066  -3.652653185  -2.534066325  -3.873286515  -2.821652271  -3.271740266  -3.064548368  -2.496920864  -3.262712373  -3.279045077  -3.029293144  -2.676621087  -3.031232042  -3.693917011  -2.439587829  -2.525423293  -2.741688026  -2.441458486  -2.735671007  -2.95072542  -3.055928213  -2.723203036  -3.259916715  -2.695777071  -2.869227759  -2.484034797  -4.433529543  -2.498046932  -3.651668189  -3.528285579  -3.629424494  -2.430971759  -2.825772094  -3.202081385  -3.279886125  -2.456530951  -2.971096896  -2.596206949  Inf  -2.780938621  -2.826810434  -2.430090446  -2.601692732  -2.544791713  -3.9513695  -2.391636498  -2.47448835  -2.466779681  -3.687984083  -3.274226898  -3.20954681  -3.6273819  -3.301276012  -2.453762612  -2.493937338  -3.087159576  -2.458908895  -3.154058671  -2.435464539  -3.710943287  -2.414314866  -2.380512659  -2.667132204  -3.167327039  -3.057792785  -3.155150027  -2.582323872  -2.393251068  -2.422438319  -2.417668134  5.499915404  -2.443539943  -2.36622304  -2.385524884  -2.43122307  -2.36270116  -3.561399717  -2.44049434  -3.050121502  -3.510447296  -2.742818415  -2.324902753  -3.31038856  -2.511704903  -3.552764924  -3.385669084  -2.504566784  -2.39607054  -3.793584172  -2.509353175  -2.965638412  -2.394402329  -2.900437844  -3.417254751  -2.865327807  -2.35144012  -3.098853316  -2.672989552  -3.879212155  -3.901450712  -2.349329982  -3.069336706  -2.428795426  -3.740651161  -3.673654467  -2.349780228  -2.466975538  -2.423159698  -2.696742611  -2.987306688  -2.506753555  -3.296748244  -2.370393088  6.753641236  -2.884977025  -2.790649323  -2.357089948  -2.346903251  -2.672803387  -2.331149483  -2.423328306  -2.328619038  -2.67319982  -2.285292918  -3.64265613  -3.13060476  -2.226893769  -2.254727348  5.440452074  -2.323516941  -2.318001505  -3.533008787  -2.310224544  Inf  -2.923605975  -2.329298836  -2.263283708  -2.237054291  -3.194345705  -2.880832802  -3.463153255  -3.021371442  -2.286667132  -2.540025359  -2.291206653  4.454746194  -2.290606236  -2.345549034  -2.370414436  -2.244814046  -2.312604386  -2.364195543  -2.293918992  -2.273374033  -2.209378155  4.816148047  Inf  -3.222632712  -3.383774801  -2.297843318  -3.552943546  -3.666725035  -3.750810996  -2.313130072  4.738763633  -3.323287585  -3.87320717  -2.720987083  -2.411591275  -4.281912676  -2.401239627  -2.310457035  -2.275076211  -3.111543019  -3.682811324  -3.379761014  -2.178812198  -4.256918036  -2.869493702  -2.892400459  -2.211546875  -3.059156155  -3.024459406  -4.727053616  -2.278730565  -2.226475434  -2.232014065  -2.641661248  -3.162411658  4.741397891  -2.2122461  -2.806692622  -2.186871213  -3.188520265  -2.163743821  -2.169339398  6.356427515  -3.481293009  -2.641161037  -2.888999222  -2.445613331  -3.287033263  -2.150592229  -2.326532655  -2.18855256  -3.025000647  -2.612102416  Inf  -2.260533303  -2.225440176  -2.43925487  -3.239159073  -2.549685853  -2.51967186  -2.399547469  -2.203680757  -2.173996942  -2.965431059  -2.169589046  -2.151368952  Inf  4.642064499  -2.151467897  -2.378537115  -3.154459878  -3.614606962  -2.233131864  -2.110984768  -2.908527245  -2.148164304  -2.197422158  4.38262624  -2.122109904  -2.153362544  -2.132609091  6.198714684  -2.14153265  -2.158888665  -2.301814152  -3.470813085  -2.144934817  -2.100062402  -2.197662182  -2.941977707  -3.220070901  -2.246135958  -2.123689222  -2.280570406  -2.08612015  -2.273682917  4.461965695  -2.603975516  5.360402515  4.087483464  -2.117200854  -2.067199313  -2.853668868  -2.061713805  -2.503001178  -2.031036013  -2.41252311  -3.00066849  -4.232122612  -2.058432041  -2.062125418  -3.103253559  Inf  -2.094144958  5.894074289  -2.329860814  4.026765434  -2.836426973  -2.483065664  -2.056679782  -2.088143104  -3.243960111  -2.132557375  -2.042874983  -2.253146141  3.977277338  -3.29460137  -2.104879571  -2.104358578  Inf  -2.630286729  -4.351061779  -3.260567443  Inf  -2.789782742  -2.452273983  -2.050644056  -2.078800234  -2.016321588  -1.908653772  -2.041415426  -2.011762244  4.039940669  Inf  -2.108022637  -2.066001756  -2.106392919  5.176830337  -2.048520404  -2.116735235  -2.915695627  -2.162070488  -2.121917358  Inf  3.891098295  -4.213895028  -2.782407914  Inf  -2.561799513  -2.020871902  -2.414284676  -2.164947782  4.555622071  -3.013700856  -2.120033605  -2.016063113  -2.249464425  -2.346543718  -2.322935369  -2.311563617  -2.09336599  -2.077660684  4.345419879  -1.992701402  -2.291314036  -2.366514829  -2.318903872  -2.466514653  5.679468039  -2.72522439  -1.96278674  -2.087840788  -1.997699436  -3.264709188  -3.596019161  -2.274426898  -2.903183732  -2.003050499  -2.023152636  -2.01324891  -2.681712061  -2.834824545  -2.442235356  -2.425938114  -2.059434103  3.496389483  4.182669149  -2.009514376  -2.680216559  -1.991735027  -2.916936041  -2.570142853  -1.987162627  -3.040260979  -2.777398705  -2.03296909  -2.658023078  3.68601794  -3.35387576  -1.963607515  -2.42742051  -2.941955102  3.73847254  -2.941556603  -1.96336496  -2.008959162  -2.061109075  -1.959023478  -2.008836331  -2.073366091  -2.054927916  -2.488769539  -1.953807015  -2.026298316  Inf  -2.055584155  -1.981532612  -3.456722456  3.757743171  -1.937442434  3.549116881  3.683453228  -1.922840908  -1.972342258  -3.155798625  -1.960959097  -2.031583791  -3.365957547  -3.057420872  3.898728994  -1.957047078  -2.115030793  -2.387124977  -2.233440354  Inf  -2.334935814  -2.361107652  Inf  3.64751967  -1.981448774  -2.611533482  -3.492778803  -2.452219289  -2.51036738  3.674841453  -2.726321319  4.157516004  3.585217534  Inf  -2.034991229  Inf  -3.173920082  -2.999396643  -3.198396073  -1.98278104  -1.999785135  -2.828727726  -2.030807975  -2.067857199  -2.421643711  Inf  -3.256870187  Inf  -2.192388916  -2.870862763  -1.875537942  3.989825031  -2.218383993  Inf  -2.987748336  -3.074620995  -2.769400051  -1.902938408  -1.987765967  -2.051357909  3.897136577  -2.133981401  -1.962656357  -2.577227494  -2.008469107  -1.869517167  -2.80691747  -2.890486676  -2.053866986  -1.915088101  Inf  -3.033549827  5.324239996  -2.300794761  -2.849491935  -1.877065287  -1.916761727  -1.947626837  -2.995021079  -1.935739706  -2.364000475  -2.506940387  -1.863058843  -2.150589206  -1.905413053  -2.954409848  -1.912968955  -1.868396527  -2.265181786  -1.900950095  Inf  -1.901797484  -2.622732459  5.723010621  -1.945849284  -3.186485713  -2.108353338  -2.530785593  -1.97336382  Inf  -2.595644613  Inf  3.526928517  -2.349157167  -1.881331602  -1.888366559  -2.891394591  3.285331639  -1.909990358  -1.827692718  4.117825457  -3.192188763  -1.952134554  -2.405503071  -1.864093613  -2.845216216  3.461185809  -1.898570461  -3.157994891  5.176732315  -2.724861017  Inf  -1.874803777  5.353084234  3.320347072  -3.355480058  -1.861727007  -1.842666072  -1.887667953  3.732881039  -2.482758017  -3.035584242  3.225160564  -2.612855408  -1.881446943  -2.022058156  -1.875809164  -1.823113214  -1.811430643  -2.058148349  -1.928877935  3.762305968  3.712102051  5.169483093  4.041908221  Inf  3.212016663  5.248422705  -2.003600021  5.242560581  -2.303804463  -2.436287577  3.166942995  -5.044096548  Inf  3.405653397  Inf  -2.037530773  -3.220304737  -1.78766856  -2.341579955  -1.854467285  -2.966815174  4.630908499  -1.893335368  -2.798630227  -2.122753981  Inf  -2.538792814  3.750104061  -2.136166062  Inf  -1.911092138  -1.786466624  -2.714866272  -2.948949705  Inf  -2.894480219  -2.210149635  -1.87256689  -1.892426013  -1.825622098  -2.021982079  3.780026206  -1.869933341  3.864523203  -1.804084608  -2.889454136  -2.376276199  -3.404425319  3.016295642  -2.014255313  -1.92728769  5.354607977  -2.733703947  -1.836442979  -3.131730936  -3.142428984  Inf  -2.02380705  3.290002212  -3.304540048  -1.814236763  -2.699729073  -1.813552991  -1.746908462  -1.787439009  -1.748323561  5.10825625  Inf  -1.808862149  -1.769572814  -1.827698102  -2.736767562  Inf  5.111584576  -2.916261528  -2.070410577  Inf  3.04028433  -2.58914176  -1.825696372  -2.026359904  3.036245247  -1.817946449  Inf  -1.821628488  Inf  -1.775334972  -1.73177642  -1.780667587  -1.925905883  -2.506284373  3.287767582  -2.308794422  -1.827154008  -2.48490599  5.039957606  -2.374334423  -2.002744767  Inf  Inf  4.42742152  3.51780132  -1.813956212  3.094915326  3.385697454  Inf  -1.79693635  -1.72781195  -2.484809122  -1.835406137  -2.360078901  -2.477156893  -2.925557557  -2.071885085  3.605111104  -2.768756737  -2.631977197  -1.825334849  -1.767229518  -1.906408224  -1.843427204  -1.782384603  -2.175048461  -1.800696971  -1.769495365  4.875927346  -2.903571914  Inf  -1.724409845  -1.847874213  2.865811675  4.844612814  -2.084668911  Inf  Inf  3.625083978  -1.772688176  -2.304084415  -1.729456042  -1.718391379  4.790319286  3.39210315  -1.679508888  Inf  -2.843603692  -1.774568258  -1.74516485  3.140792495  -1.652294345  -1.71367044  -2.301241919  -2.701309323  -1.681790456  -1.910070663  -1.743382474  -2.279690209  3.264228723  -1.685292813  -2.641540473  -2.721761191  -1.785327571  -1.68605792  -1.783770301  -2.065922813  -2.807687572  -1.816422601  -3.370972905  -1.769890581  -2.461271861  -3.689117384  -1.748006912  -1.863581347  -2.158846046  -2.28603562  -2.108565833  -1.949437472  -1.681929058  -2.224026997  -2.111437201  -1.71716912  Inf  -1.757028547  -2.116678392  4.266142233  -1.73027735  3.533706101  -1.715584446  -2.114768364  -1.749369933  -1.732482775  Inf  Inf  -1.893394271  -2.2395035  -2.162908762  -1.746469212  3.383735373  3.085399347  -2.489128228  -2.818799714  -2.121967071  Inf  -1.717471477  -1.804683655  -2.190771544  3.588648449  4.08470304  Inf  -2.028143629  Inf  3.486873013  -2.217959061  -2.616137471  4.092140054  -1.767860169  -2.111324513  3.623368202  -1.667970422  -2.113917826  -1.700663038  -1.788468831  -3.407798988  -1.625947069  -3.313930644  -3.007452266  3.343051791  -2.060552233  -1.70577836  -1.686239029  4.737969373  -1.721020074  -1.769981232  2.835813176  Inf  -1.739471855  -1.642646754  2.831664065  Inf  3.024681121  -1.65474317  -1.867840251  4.04937201  -1.668592505  3.095198811  3.246305154  4.117610696  -2.409698186  -2.371590499  -2.324496945  -3.601925182  3.376049157  -2.492497259  -1.698959757  -2.048843206  2.980317755  Inf  -1.71508534  -1.731223512  Inf  -1.57698628  -2.040364035  3.538186081  -3.382180668  -3.63342285  -2.004012596  -2.33550912  3.147509692  -2.937609211  -1.613279612  -2.065784626  -1.708514088  -1.690457025  -2.229039256  -1.603945149  -2.146066876  3.105662837  -2.766192473  -2.788509938  -2.036414597  -1.730898289  -2.133921853  4.590291028  -2.22395538  2.87301264  -2.135242069  -1.818227905  -1.605435608  -1.689231862  -1.673355189  -1.609510449  5.486806497  Inf  4.920277932  -1.629245162  4.043399362  -2.474063126  -1.71145163  -2.429722629  -1.712917324  Inf  -1.660917445  -1.710403547  2.731016842  3.941072405  -1.662739716  -2.242400731  2.714359553  Inf  -1.762364089  -1.673336744  -1.62329256  -4.751954627  -2.231191744  -4.7051469  3.464597518  3.336920057  -2.619493839  -2.163519233  Inf  Inf  -1.701789113  -1.531926779  -1.648504849  -3.129351274  -1.674982074  -3.719504236  Inf  -1.683859576  -1.668355089  -1.697616608  -1.613212357  -2.363201732  Inf  -2.37278421  Inf  -2.088802415  -2.025914368  -1.621662473  -2.010903711  2.61984534  -1.686510991  -1.597054863  Inf  -3.203743967  -2.074109943  -3.393784198  -2.006864368  -2.337518583  -2.102299464  -1.727095544  -1.651939376  -1.68470774  -1.690446056  4.607848826  2.633393925  -2.026773482  -2.408962179  -2.249611976  2.905554015  -1.721722675  2.900610351  -2.071414121  -1.630757615  -1.957152024  Inf  -2.240558587  2.75386909  -2.478905719  -4.319012644  4.472820638  2.896987752  3.569130597  -1.671119751  -2.158485425  -1.801134846  -1.750270536  -1.607213238  -1.669996992  -1.599657623  -2.558922846  -1.640466674  3.24341352  -2.396970696  -1.577429667  2.678587771  -1.570213244  Inf  Inf  -2.527357255  -1.7971929  2.98819373  -1.593177096  -1.64635373  -2.432228738  -1.620941419  -3.341350226  -1.950637545  4.058437057  Inf  -3.552233779  -1.673427306  -2.294272049  Inf  -1.685954088  4.0522349  -1.658742154  -1.811553464  3.566620915  -1.610067433  -1.651246598  3.515825166  2.713089979  3.25104469  -1.618281259  -1.968694589  -2.559214239  2.799294297  -1.577826751  -3.536127061  -1.660704091  -1.706251508  -2.026324945  3.211015768  -1.826741266  -2.947340916  -2.469775633  -3.044548497  -1.633788441  -1.632035984  Inf  -2.664616713  -1.651151216  3.116675718  -1.590373553  -2.267664518  2.559658559  -1.639469597  -1.746595924  -1.694700714  2.471823341  Inf  2.920131119  -2.013904904  2.796812707  3.451580768  3.300327485  Inf  -1.919136251  -1.705407701  -2.057344007  -2.054257442  -1.546872206  2.628961715  -1.910266267  -1.613320777  -1.922108292  -1.5964046  -1.643508259  -1.568141773  -2.968701065  Inf  -1.573634562  Inf  -1.591054826  2.614963352  2.704949643  Inf  -1.609227004  -1.641004799  -1.534563332  2.437974557  -4.097150146  -2.351741215  -2.080772802  -1.746737319  -2.542431688  -1.529031548  -1.601320485  Inf  -1.578361215  Inf  -1.601781873  -1.647468629  -1.600571176  2.522481665  2.424856691  -1.505973807  2.546068874  -1.560705638  -2.467385482  -3.418760149  -2.116493916  3.751170912  -1.592314579  2.641031786  -2.675040422  -1.57741957  -2.425188093  -1.53833191  -1.720999216  -2.32752688  2.643718235  5.999520631  3.542706225  -2.614826544  2.404323073  2.728981652  -1.696501307  2.844262458  -1.86543846  -2.569646782  -1.57451405  -2.026266554  -1.548680909  -1.567734597  -3.082425777  2.748384866  -2.58081548  -2.279526806  Inf  Inf  2.708703455  -1.891341625  -3.276698429  3.34299925  2.480618001  -2.179176566  Inf  -2.85499855  Inf  -1.480299488  -1.607097451  2.739373374  Inf  3.071897305  3.445796469  3.063778876  -2.313429803  -3.099341504  3.344402277  -2.096861715  -1.491900579  -2.164759556  2.791020994  2.421406029  -1.558022098  -1.738040992  -1.562876432  -1.687957403  -1.683753486  2.513278843  -3.095179466  Inf  -1.526835881  -1.763346581  2.884818011  -1.599723087  -1.930076108  Inf  2.954358196  -2.436847227  -1.529018398  Inf  -1.669207693  -2.348850343  -1.595542931  Inf  -1.551899851  2.414358415  2.960680663  Inf  -3.085194751  Inf  2.99179815  -1.459779432  2.334499991  Inf  2.878690271  -1.526197395  2.737406881  2.513792153  -1.469244969  2.347031419  3.313328822  -2.303705969  Inf  3.167902365  -1.669840612  -2.423066855  2.997538008  2.994058142  -2.529523575  -1.79344482  -1.465006824  4.265638205  -1.511997642  Inf  2.832753947  -2.201258516  2.883734199  -2.445429041  2.532436471  -1.746355017  2.618932061  -1.66060735  4.45269688  2.869538349  -2.365505816  -4.400799995  -2.398246901  3.091212288  -1.702076688  -2.896077774  -1.862759236  -1.822185838  -2.468342174  -1.570688097  -2.463701469  6.004968735  -1.447570618  -2.425036626  -2.108601824  -1.515435535  -1.549034319  -1.522846459  -1.529652064  -1.777531769  2.502134651  -1.54001321  2.291513579  -1.720379973  -1.527065714  Inf  2.887386282  -1.844261971  2.604459792  3.238631706  -2.644719023  Inf  2.333375026  2.6907436  2.616197891  2.93628898  Inf  -2.029376113  -2.544036931  -2.443034299  2.383052716  -1.830790593  -1.462671808  -1.550415303  -1.519186099  -1.496859147  -2.219731128  Inf  2.758891176  -1.770867206  2.965677914  -1.461970867  -1.582367749  -1.490708443  -1.652077263  -2.869360175  2.923029446  4.162289038  -2.270866149  2.293994061  -1.505817568  2.485905035  Inf  -1.957064989  2.733577342  -1.530117632  2.554725033  -1.528249243  -5.185196483  -1.524654451  -1.561277588  -2.736261415  3.00232524  -2.462000845  2.629588873  -1.701769161  -2.249444904  -1.454725408  -1.926296912  -1.590964589  -1.773857821  2.746696147  -1.45676026  -1.759282946  Inf  3.488532341  Inf  -1.501812385  -1.515860371  -1.563263175  3.545045894  -1.518610163  2.47727287  -1.510365525  Inf  3.242621423  Inf  -1.663475041  4.249873026  -1.469934988  -2.032229435  -1.634190191  -2.336357129  -1.972995185  -1.479388026  -1.502045232  Inf  -1.38950227  -1.719766436  Inf  2.356508058  -1.501489259  -1.438506718  Inf  -1.503718478  4.403807186  2.556148763  -1.465932334  -1.491022972  -1.759585419  Inf  3.313765713  3.154534265  3.246433324  -2.143508579  5.068708999  -1.849805368  2.92678269  -1.442884318  Inf  2.199590377  -1.51009798  2.716932966  4.227332619  -1.479210716  Inf  4.255878368  -1.423160482  -1.525124144  -1.999261155  2.524742384  -1.502996892  2.625185389  -1.431609304  5.76976337  -1.567949429  -1.419654905  2.709405022  -1.446094365  2.747834558  2.16688624  2.521835564  -1.419657369  -1.48058812  4.143497119  3.896554549  2.235210519  2.917821483  2.153460005  -2.005088528  2.957490441  -1.482662659  -3.597782619  -1.501971782  -2.013946087  -1.349475797  -2.161217833  -1.452459845  -1.453206864  2.232341052  -1.493639378  -1.542459662  -1.483136135  Inf  -1.468033535  -1.523701726  Inf  3.442483321  -1.43799879  -1.624521262  -2.266585326  Inf  -1.409322721  2.462555429  -1.869749796  -1.910073869  Inf  -1.454920795  3.914471213  2.29435581  Inf  -2.38797249  -1.396436669  -1.454729641  4.086950158  -1.59067669  -2.467659035  -1.460780023  -1.386377178  Inf  2.210438188  2.776393197  3.47792982  -1.545429008  -1.651304244  -1.709941351  -2.201459716  2.237170579  -1.469761495  -1.47223974  -1.526280213  -1.701730749  -2.30691407  -1.546342036  -1.383213013  -1.870570064  -1.568636273  -1.555915552  -1.858911418  2.574157485  -1.362585122  2.515361068  -2.401595854  -1.421764729  -1.435659829  3.072628968  2.985804134  Inf  -1.959901518  -1.431764933  Inf  Inf  2.153880787  -1.43275811  -1.388378555  -1.382766085  2.164469857  -1.555274612  -1.726588793  -2.124638104  -1.720343656  -1.465909268  Inf  Inf  -1.454821203  2.188972912  -2.185963289  3.002978779  4.174430194  -2.141516443  -1.367996041  Inf  -1.412635707  2.285816092  Inf  2.35629515  -1.487009244  -3.502130893  -1.410366889  -1.464521243  2.397423773  5.264715417  2.501882725  -1.748640892  -1.850944603  Inf  Inf  -2.611668999  -1.443893207  -2.024050103  -1.426490523  Inf  2.345367872  2.476994051  3.04198473  -1.552770633  4.106979132  -1.414410441  2.099549128  2.295676886  -1.61668033  -1.778164784  -1.341833511  3.062408713  -1.688884989  -1.633226899  3.958643869  -1.440718421  2.140036544  -1.610256274  2.397826437  -1.381362449  3.032723224  -1.655605326  -1.487374094  -2.233320905  -1.404648993  3.03022051  -1.424441394  -1.526841068  -1.381156354  3.406428058  -1.368461125  Inf  -1.72572203  -1.462559967  -1.442454904  2.165114183  -1.351529537  2.177554089  2.083247819  -1.407305769  2.682363515  2.5870047  3.302913377  -1.424517825  4.962000319  2.433051359  Inf  -1.378198347  -1.396081764  -1.354053705  3.005253159  2.144200034  -1.399939654  2.77132797  Inf  2.591861049  -1.406320938  -1.471230433  2.138346961  -1.409302189  -1.689993849  -1.40515655  -1.550476602  2.249678047  -1.449159156  2.900804975  -2.603873114  -1.791007734  Inf  -1.588604147  -1.411852779  2.658092284  2.101403293  Inf  3.124908542  -1.370972019  -1.791801876  -1.357283316  -1.500363809  -1.436824984  -1.380163687  3.876891942  -1.369911777  -2.056069004  2.345846531  -1.545848132  2.543361544  2.736147782  -1.417026926  -1.471939833  -1.593826597  -1.365761977  -1.365329394  2.462836328  -1.462867347  -1.723536405  2.548532858  -1.584851349  -1.370303427  -1.379568737  -1.340264881  -1.344575033  3.209429779  Inf  -1.384856437  2.026923522  -1.849258285  -1.348288479  4.095332921  -2.289328235  -1.368400281  -1.42528306  -1.570624001  -1.345521732  -1.657630552  -1.878208655  -1.543796448  -1.362338502  -2.124467373  -1.391020213  Inf  Inf  -1.320772187  Inf  3.061812565  2.929880324  -1.673431107  -1.469221688  -1.426224974  2.738309581  -1.877605215  Inf  -1.429311304  2.11839762  -1.93331275  -1.513172209  -2.345476214  -1.997740101  -1.431025741  -1.311459932  2.036323684  -2.452130719  -1.359229752  -1.825750839  2.608466569  -1.573329906  -1.75474437  4.360837589  -1.38154652  2.135748953  4.015748474  -1.360932194  -2.186847669  -2.745093379  -1.537934683  2.051169575  4.385392837  2.070292649  -1.614532986  -1.348204222  2.581518427  -1.400409661  -1.338741798  2.927599011  2.671866517  -1.390063922  Inf  2.180968885  Inf  -1.489088916  2.163531892  -1.678216507  2.250255562  -1.388085637  -1.474477171  2.438632866  -1.593448845  -1.566719988  -1.352322465  -1.242417797  4.037070412  Inf  -1.340497135  2.780381268  -1.720245515  -1.310679539  -1.393046055  -1.396562976  -1.479322783  2.079984908  -1.351387058  2.948776985  -2.19394823  2.133134119  -1.950067823  3.972520019  -1.777579602  -1.309961738  -2.054305356  Inf  -1.278527192  -1.405695654  2.140576562  1.98169678  -1.29990024  -1.337975657  -1.29838734  -1.29153628  2.115137498  Inf  -1.349921286  -1.512036566  2.336609475  2.464318188  -2.251821731  -1.421239027  -1.279654617  2.555296403  Inf  3.37766315  3.451475543  -1.498988548  4.022158624  Inf  2.086540133  -1.330443721  -1.313213763  2.028221441  2.190563263  -1.355127338  -1.883793279  2.222906105  4.354679437  -1.335517715  -1.258570235  2.179315672  -1.356559425  -1.693474123  Inf  -1.345196627  -1.334471606  -1.677511494  3.774564752  1.988484898  -1.27694157  2.145538176  -1.566379566  -1.402811451  -1.727345744  -1.324949702  -1.632920375  -2.046688872  -1.537715877  2.526309301  -1.363816371  Inf  -1.352038954  Inf  2.511237266  2.091154661  -1.556606812  2.288549234  2.255117709  -1.907471052  -1.323934795  -2.142845874  -1.455992765  Inf  1.973631823  -1.843463824  -1.404401913  Inf  Inf  -1.581055803  -1.521865776  Inf  1.954573647  2.756956369  -1.429389487  2.433008737  2.501757502  -1.284122254  -1.83723755  -2.002129976  Inf  -1.705526899  -1.492910242  2.518769597  -1.948376038  -1.987336922  2.294198638  Inf  2.165814167  Inf  1.94596203  4.363803946  -1.32971103  Inf  -1.323471956  -1.391014896  Inf  -1.84007587  2.539149978  2.076507084  -1.345532108  -1.300198898  -1.973589372  Inf  Inf  -2.201663583  -1.389348713  2.514627629  -1.329798763  1.976028582  -1.310584229  Inf  Inf  1.901904823  -1.329002708  2.141664589  2.628715549  -1.858640541  -1.293616799  -1.983746184  -1.468863193  Inf  -1.793015893  -1.266470713  -1.856968524  -1.950094495  -2.339782302  -1.619752627  -1.352028429  -1.238693731  Inf  Inf  -1.335003271  -1.26522143  -1.293842218  Inf  1.924644667  Inf  -1.303384543  -1.790298489  -1.859871347  2.043474935  -2.033220067  3.119042914  -1.296039381  Inf  -1.798760644  -1.263370031  -1.439260113  2.210306733  Inf  Inf  2.276553823  Inf  3.942453353  -1.275627485  2.532403321  2.394340553  2.45900602  -2.221896243  -1.89319834  1.815545105  -2.017469698  3.940551623  -1.316624697  Inf  2.859409566  Inf  -1.739899894  -2.036304656  2.452159002  -1.459758926  -1.600971933  -1.302866761  Inf  2.973812021  -2.208679292  -2.84687773  Inf  -1.558939945  -1.255813494  1.858425172  -1.167740352  Inf  -1.937067274  2.021613734  -1.300709262  -1.306852462  -2.055065937  -1.622413724  Inf  2.498753726  -1.370647321  Inf  -1.377955433  -1.242501217  -1.728098957  -1.48268066  1.847491087  4.055481594  2.462046569  -1.765952636  2.098402099  2.405594026  2.263308982  -1.829079066  1.858066943  2.202513336  -1.558504766  3.894035141  Inf  2.425921093  Inf  Inf  Inf  -1.782712842  -2.37281702  2.23118519  Inf  2.127385875  2.183975052  2.540090356  1.927180445  -1.678184271  Inf  3.035524945  Inf  -1.278326203  -1.27194891  Inf  -1.311056462  2.177569949  2.029884764  -1.30029126  5.480586192  -1.292799624  2.415006797  2.988974142  2.123880087  -1.188799663  1.837354081  2.561749306  -2.061924437  -1.287492187  -1.284647349  -2.161411087  2.264543559  -1.280107267  1.84590261  -1.399902618  2.440950966  3.786214412  -1.255267518  -1.450074273  -1.301283393  2.389255615  -1.336723834  Inf  Inf  -1.267374075  1.907579278  -1.220950201  -1.230477786  2.033066579  Inf  -1.565761944  -1.547469062  2.164284394  1.872696068  3.792917248  -2.162496905  4.577320811  -1.2381239  -1.199347665  2.283554216  3.90659037  -1.484298339  -1.554855221  Inf  2.801192756  -1.27140592  -1.217674609  -1.222830823  -2.123456322  2.372535581  -1.332515619  2.372223238  Inf  -1.288560929  2.662787739  Inf  2.378320812  -1.231839056  2.119504629  3.688507808  -1.58847994  1.867901197  3.084405827  -1.270090138  -1.709963624  1.81409171  -1.342334078  -1.30599263  -1.900331133  -2.056504066  -1.472657044  2.524567689  2.170106349  2.180178424  -1.240222252  -1.381861856  -1.287176233  -1.306512604  -1.488941714  -1.277214596  -1.493761017  -1.215241076  Inf  1.877766627  Inf  -1.598897924  1.876852349  -1.278746024  -1.583503632  Inf  -1.25616353  Inf  -1.245215044  1.872882105  2.161556502  -1.426145995  -2.118972058  -1.138151802  -1.231119027  2.342442393  -1.852194837  -1.531762092  -1.460215242  -1.167626434  -1.720963433  Inf  1.906748843  -1.275252838  -1.295919043  2.643246121  -1.236951694  -1.310409384  2.512596791  Inf  -1.409506031  2.346078502  -1.265656676  -1.213424655  3.820539031  1.797323364  -2.173602034  -1.427732491  -1.74293335  Inf  -1.205080218  3.444419901  1.848912784  3.054651103  -1.23856036  2.480815105  2.059207851  -1.278275952  2.356804621  -1.212775694  2.038466021  2.181355433  -1.866739872  2.714929318  -1.278966962  2.08071151  -1.26251854  2.103118735  -1.661267284  -1.638564016  2.148840205  -1.536082058  2.293416714  Inf  -1.32580001  Inf  2.122227166  2.34745072  -1.259323227  3.788414261  2.617201391  2.226047135  -1.260918843  1.75762186  -1.86822037  1.85754141  2.094581511  -1.530605156  2.3008564  2.199593044  -1.268780905  Inf  2.112458841  Inf  -1.247910738  3.047767825  2.109647144  -1.478984692  -2.111831355  -2.733161634  -1.724472527  Inf  3.34298993  Inf  -1.261789737  -1.246454054  1.995724673  1.845505596  2.406436158  -2.4148057  1.905184506  2.986867646  2.238346901  -1.299485155  -1.515703994  -1.230266469  1.860264332  -1.60159722  2.321556552  Inf  1.944428455  1.819920529  2.318963746  -1.515005058  2.119506442  -1.292399036  -1.238927404  -1.249640521  -1.260524145  -1.237495012  -1.193453483  -1.579584907  -1.209807682  1.978424179  Inf  -2.266109995  2.596882105  2.319726159  2.318880285  1.809882024  -1.266919243  2.058318937  Inf  -1.61851607  2.147622802  2.036090741  2.805800005  2.251137393  2.563416431  1.754035723  2.446347533  -1.175814577  Inf  -1.213334184  -1.430318298  -1.522585952  -1.311530761  2.973098313  Inf  -1.170429088  Inf  -1.222858103  -1.697681338  -1.653349049  -1.27636465  2.140042203  -1.228229721  -1.253519358  1.818541258  -2.054345407  2.18947124  -1.839412212  -1.219878212  -1.29527493  1.853301153  -1.259357549  2.029929362  1.969624969  Inf  -1.319790415  -1.680333382  1.905192381  2.193949593  -1.277838632  2.010652923  -1.229935657  Inf  1.811361239  -1.222929895  -1.226567589  -1.201028021  1.744592557  -1.326352541  2.574600262  -1.229038574  -1.180424714  2.282565474  -1.30972101  1.819658574  -1.487864988  -1.875452546  Inf  -1.543126122  -1.200047922  Inf  Inf  Inf  1.898575799  -2.035052171  2.643436717  -3.357732899  -1.169251639  -1.212839107  -1.205126283  -1.36183355  2.060538068  -1.522146223  -1.408582516  Inf  1.98475203  -1.496642819  -1.10806526  2.087732331  2.625545548  1.792106538  -1.36488533  1.765990415  Inf  1.80752981  2.161214609  2.534724959  -1.236315961  1.740975533  1.984928589  -1.228137324  1.794629515  3.01261437  1.695641832  Inf  -1.376836688  1.739963354  Inf  1.800850054  -1.174185946  2.223986456  -1.207245653  Inf  3.556262723  1.712629866  -1.246435597  Inf  -1.286974647  2.383800763  -1.857288319  3.714628079  -1.29838186  1.701121613  3.937967948  -1.198995894  1.946056846  -1.487576213  1.848131161  -1.464908478  2.888789609  -1.169445769  -1.185206858  2.512735918  2.544445493  -1.197534107  -2.046140286  -1.171381723  2.782881438  -1.209762137  1.830698506  -1.180931125  1.793087116  -1.175882217  Inf  1.940133968  -1.149542658  2.260554442  3.685067482  2.113815504  -1.221384523  -1.408268073  2.278187747  -1.28643021  -1.531155219  1.785669968  Inf  2.206699414  -1.419490726  1.689635922  1.744953721  -1.844800663  1.68740765  -1.183372044  Inf  -1.200927243  1.835513549  -1.071596278  1.6704359  3.68165227  2.208547069  2.596109499  -1.225808529  -1.156245103  -1.129993975  2.300392047  1.827076757  Inf  -1.219797684  1.682601531  -1.088955903  3.589830897  2.585910648  -1.421046087  1.768252604  -1.722967751  2.205936458  -1.086352585  -1.302983463  -1.63771284  -1.179828744  -1.13827359  -1.286739152  -1.329784748  -1.177935496  Inf  -1.186341519  2.415005991  2.236619324  1.699150864  2.094899791  1.756954755  Inf  Inf  1.661465339  -1.167698474  -1.13231514  Inf  1.704419503  1.612071083  1.753425851  -1.548156893  -1.63203385  -1.383430547  -1.195788726  -1.825979354  1.804760575  Inf  3.694335708  -1.230400509  -1.189013605  -1.994051624  -1.765099665  -1.164499663  -1.903588111  2.220333887  -1.489343704  2.107686499  -1.549750383  -1.322796379  Inf  1.65164519  -1.60964708  1.689908227  Inf  1.697798223  1.617406296  Inf  Inf  2.294982803  Inf  -1.188559921  -1.12502032  3.71485511  1.670344756  1.940094886  -1.230996132  -1.806245669  1.72094333  2.168886432  -1.265862097  Inf  1.707136266 |
