# Supplementary material for: CircularLRRC7 is a Potential Tumor Suppressor Associated With miR-1281 and PDXP Expression in Glioblastoma
Source: Front Mol Biosci. 2021 Nov 29;8:743417. doi: 10.3389/fmolb.2021.743417 (PMC8667166; doi:10.3389/fmolb.2021.743417)
Supplement: Supplementary file 4 [file Table3.DOCX]

**Supplementary Table 3. Identification of differentially expressed miRNAs (DEmiRNAs) between GBM and normal brain tissues.**

| miRNA_ID | .P.Val | t | B | Log_2_FC |
| --- | --- | --- | --- | --- |
| hsa-miR-21  hsa-miR-139-3p  hsa-miR-1825  hsv1-miR-H6  hsa-miR-155  hsa-miR-193a-3p  hsa-miR-551b  hsa-miR-136  hsa-miR-1305  hsa-miR-21  hsa-miR-630  hsa-miR-532-5p  hsa-miR-892b  hsa-miR-210  hsa-miR-148a  hsa-miR-765  hsa-miR-500  hsa-miR-548c-3p  hsa-miR-296-5p  hsa-miR-590-5p  hsa-miR-18b  hsa-miR-1915  hsa-miR-505  hsa-miR-638  hsa-miR-574-5p  hsa-miR-572  hsa-miR-664  hsa-miR-181a-2  hsa-miR-125b-2  hsa-miR-1288  hsa-miR-34a  hsa-miR-575  kshv-miR-K12-3  hsa-miR-139-5p  hsa-miR-483-5p  hsa-miR-30e  hsa-miR-1914  hsa-miR-142-3p  hsa-miR-199a-3p  hsa-miR-660  hsa-miR-144  hsa-miR-27a  hsa-miR-497  hsa-miR-92b  hsa-miR-150  hsa-miR-130a  hsa-miR-601  hsa-miR-1268  hsa-miR-23a  hsa-miR-1281  hsa-miR-1207-5p  hsa-miR-886-3p  hsa-miR-652  hsa-miR-940  hsa-miR-324-5p  hsa-let-7a  hsa-miR-1275  hsa-miR-513a-5p  hsa-miR-381  hsa-miR-425  hsa-miR-671-5p  hsa-miR-939  hsa-miR-106b  hsa-miR-1249  hsa-miR-1246  hsa-miR-376a  hsa-miR-195  hsa-miR-1225-5p  ebv-miR-BART13  hsa-miR-1202  hsa-miR-1308 | 9.39E-08  1.00E-07  1.03E-07  2.70E-06  3.44E-06  6.38E-06  8.41E-06  3.71E-05  6.42E-05  1.24E-04  1.60E-04  2.06E-04  2.10E-04  2.11E-04  5.52E-04  6.51E-04  6.66E-04  6.73E-04  6.91E-04  7.37E-04  8.17E-04  9.91E-04  1.17E-03  1.34E-03  1.54E-03  2.16E-03  2.34E-03  2.79E-03  2.99E-03  3.03E-03  3.48E-03  3.74E-03  3.95E-03  4.06E-03  4.22E-03  4.45E-03  4.57E-03  4.62E-03  5.46E-03  5.73E-03  5.99E-03  7.37E-03  9.24E-03  9.26E-03  9.89E-03  9.98E-03  1.03E-02  1.34E-02  1.36E-02  1.41E-02  1.41E-02  1.47E-02  1.54E-02  1.65E-02  1.70E-02  2.02E-02  2.10E-02  2.14E-02  2.17E-02  2.31E-02  2.35E-02  2.45E-02  2.50E-02  3.11E-02  3.41E-02  3.45E-02  3.68E-02  3.94E-02  3.97E-02  4.17E-02  4.86E-02 | 6.57291  -6.55137  6.54192  5.50799  5.4306  5.23456  5.14657  4.66971  -4.49148  4.27536  -4.18972  4.10612  -4.09897  4.09846  3.77194  -3.71544  3.7073  3.70381  3.69455  3.67226  3.63656  -3.56917  3.50989  -3.46387  -3.41201  -3.29113  3.26253  3.19836  -3.17209  -3.16786  3.11639  -3.08986  -3.06951  -3.05867  -3.04404  3.02444  -3.01446  3.01017  2.94721  2.92825  2.91125  2.83157  2.74311  2.74199  -2.71596  2.71231  -2.70069  -2.59262  2.58732  2.57327  -2.57195  2.5565  2.53708  -2.5083  2.49635  2.42507  -2.40792  -2.40108  2.39386  2.36854  -2.36083  -2.34238  2.33323  2.23856  -2.19764  2.19328  2.16379  -2.13361  -2.12986  -2.10811  -2.03782 | 7.578  7.566  7.434  4.531  4.322  3.771  3.545  2.192  1.699  1.04  0.822  0.646  0.626  0.625  -0.244  -0.397  -0.404  -0.411  -0.434  -0.492  -0.585  -0.859  -0.903  -1.146  -1.213  -1.496  -1.513  -1.67  -1.758  -1.756  -1.877  -2.003  -2.084  -2.005  -2.094  -2.135  -2.149  -2.13  -2.261  -2.299  -2.353  -2.586  -2.736  -2.705  -2.832  -2.838  -2.812  -3.142  -3.181  -3.064  -3.275  -3.174  -3.152  -3.288  -3.233  -3.393  -3.523  -3.465  -3.434  -3.499  -3.545  -3.674  -3.656  -3.73  -3.969  -3.842  -3.957  -4.122  -4.033  -4.243  -4.258 | 7.30741  -5.55593  6.01296  5.62757  5.95639  6.44905  6.07661  4.86903  -3.4792  3.05635  -4.37082  4.64763  -4.23516  3.62193  5.06715  -2.9093  3.88739  4.34566  3.88065  4.5079  3.93007  -2.98637  3.83601  -2.97391  -2.65256  -2.75475  3.55199  3.42231  -2.72276  -3.27679  3.73726  -2.47076  -2.66051  -3.91257  -2.51055  2.11587  -2.31868  3.23802  3.83709  3.65148  4.79053  3.09725  3.25864  2.50983  -2.15392  2.90549  -3.14086  -2.1049  1.38968  2.49826  -2.34458  2.05675  2.93369  -1.83352  3.35236  2.50769  -2.17585  -2.39029  2.90334  2.53598  -2.51511  -1.90918  2.45009  2.56943  -2.19042  2.42457  2.65589  -1.89194  -1.63698  -1.85355  -1.0537 |
