# Supplementary material for: CircularLRRC7 is a Potential Tumor Suppressor Associated With miR-1281 and PDXP Expression in Glioblastoma
Source: Front Mol Biosci. 2021 Nov 29;8:743417. doi: 10.3389/fmolb.2021.743417 (PMC8667166; doi:10.3389/fmolb.2021.743417)
Supplement: Supplementary file 5 [file Table4.DOCX]

**Supplementary Table 4. The target genes of hsa-miR-1281 predicted by 3 miRNet.**

| miRNA ID | Target gene |
| --- | --- |
| hsa-mir-1281  hsa-mir-1281  hsa-mir-1281  hsa-mir-1281  hsa-mir-1281  hsa-mir-1281  hsa-mir-1281  hsa-mir-1281  hsa-mir-1281  hsa-mir-1281  hsa-mir-1281  hsa-mir-1281  hsa-mir-1281  hsa-mir-1281  hsa-mir-1281  hsa-mir-1281  hsa-mir-1281  hsa-mir-1281  hsa-mir-1281  hsa-mir-1281  hsa-mir-1281  hsa-mir-1281  hsa-mir-1281  hsa-mir-1281  hsa-mir-1281  hsa-mir-1281  hsa-mir-1281  hsa-mir-1281  hsa-mir-1281  hsa-mir-1281  hsa-mir-1281  hsa-mir-1281  hsa-mir-1281  hsa-mir-1281  hsa-mir-1281  hsa-mir-1281  hsa-mir-1281  hsa-mir-1281  hsa-mir-1281  hsa-mir-1281  hsa-mir-1281  hsa-mir-1281  hsa-mir-1281  hsa-mir-1281  hsa-mir-1281  hsa-mir-1281  hsa-mir-1281  hsa-mir-1281  hsa-mir-1281  hsa-mir-1281  hsa-mir-1281  hsa-mir-1281  hsa-mir-1281  hsa-mir-1281  hsa-mir-1281  hsa-mir-1281  hsa-mir-1281  hsa-mir-1281  hsa-mir-1281  hsa-mir-1281  hsa-mir-1281  hsa-mir-1281  hsa-mir-1281  hsa-mir-1281  hsa-mir-1281  hsa-mir-1281  hsa-mir-1281  hsa-mir-1281  hsa-mir-1281  hsa-mir-1281  hsa-mir-1281  hsa-mir-1281  hsa-mir-1281  hsa-mir-1281  hsa-mir-1281  hsa-mir-1281  hsa-mir-1281  hsa-mir-1281  hsa-mir-1281  hsa-mir-1281  hsa-mir-1281  hsa-mir-1281  hsa-mir-1281  hsa-mir-1281  hsa-mir-1281  hsa-mir-1281  hsa-mir-1281  hsa-mir-1281  hsa-mir-1281  hsa-mir-1281  hsa-mir-1281  hsa-mir-1281  hsa-mir-1281  hsa-mir-1281  hsa-mir-1281  hsa-mir-1281 | EPO  PPIF  CXorf66  PDXP  GHRL  CCDC142  RORA  GCLM  PDE1C  LOR  FETUB  CACNA1G  TRMT10B  DTNA  C6orf106  TLDC2  DAG1  SYNCRIP  TIMP3  LMX1B  TLE1  SLC22A11  CRYBA4  SLC39A5  NANOS1  DHDDS  WDFY1  C5orf38  CHST5  FBF1  6-Mar  KCNK10  DUSP8  SLC1A4  PLCXD1  ZNF597  RHBDL2  NYX  RIPK4  CLPTM1  PDXDC1  POLH  GPR183  INO80D  CYP20A1  GPA33  HNF1B  VSTM2A  SCRT2  RTN2  ST3GAL2  CCDC57  HRH3  MXD4  SPIB  GYPC  DLAT  BIN2  DDX11  NAGS  ITPKB  TNFAIP8L1  MAFG  KIF1C  SPTB  CYTH2  TAF8  PPP2R2D  RNF122  UVSSA  XPOT  ZNF107  PDE6A  EGR3  DUXA  FAM20B  TOR4A  STOX2  SFT2D3  ARHGEF15  LAIR1  PAX7  PRELP  VHL  SCN4B  DLX1  TNNC2  PRNP  VENTX  DGKE  FAM20C  PCID2  GP6  SURF6  RINL  BHLHE41  DBNDD1 |
